# 14-Substituted Diquinothiazines as a New Group of Anticancer Agents

**DOI:** 10.3390/molecules28073248

**Published:** 2023-04-05

**Authors:** Małgorzata Jeleń, Krystian Pluta, Małgorzata Szmielew, Beata Morak-Młodawska, Kinga Herman, Klaudia Giercuszkiewicz, Anna Kasprzycka, Magdalena Skonieczna

**Affiliations:** 1 Department of Organic Chemistry, Faculty of Pharmaceutical Sciences in Sosnowiec, The Medical University of Silesia, Jagiellońska 4, 41-200 Sosnowiec, Polandbmlodawska@sum.edu.pl (B.M.-M.); 2 Department of Organic Chemistry, Bioorganic Chemistry and Biotechnology, Faculty of Chemistry, Silesian University of Technology, Krzywoustego Street 4, 44-100 Gliwice, Poland; kingher651@student.polsl.pl (K.H.); anna.kasprzycka@polsl.pl (A.K.); 3 Department of Systems Biology and Engineering, The Silesian University of Technology, Akademicka Street 16, 44–100 Gliwice, Poland; klaudia.giercuszkiewicz@polsl.pl; 4 Centre of Biotechnology, Silesian University of Technology, Krzywoustego Street 8, 44-100 Gliwice, Poland

**Keywords:** phenothiazines, diquinothiazines, diazaphenothiazines, anticancer activity, cytotoxicity, apoptosis, cell cycle arrest

## Abstract

A series of novel double-angularly condensed diquinothiazines with aminoalkyl, amidoalkyl, sulfonamidoalkyl, and substituted phenyl groups was designed, synthesized, and evaluated for their anticancer activity against four selected human tumor cell lines (HTC116, SH-SY5Y, A549, and H1299). The cytotoxicity of the novel diquinothiazines was investigated against BEAS-2B cells. The activities of the compounds were compared to etoposide. Among them, compounds with aminoalkyl and phenyl groups showed excellent broad-spectrum anticancer activity. The most active 14-(methylthiophenyl)diquinothiazine, **3c**, showed low cytotoxicity against BEAS-2B cells and high activity against tumor cell lines HTC116, SH-SY5Y, A549, and H1299, with IC_50_ values of 2.3 µM, 2.7 µM, 17.2 µM, and 2.7 µM, respectively (etopiside 8.6 µM, 3.9 µM, 44.8 µM, and 0.6, respectively). Live long-term microscopic observations of cell survival using the starting molecule **M0** were also performed. Flow cytometry showed the proapoptotic effects of the studied diquinothiazines. Inhibition of the cell cycle in the S phase was observed, which is associated with damage to nucleic acids and connected to DNA replication arrest.

## 1. Introduction

Heterocyclic ring systems perform essential roles in the searching of novel cores in medicinal chemistry to find new candidates for drugs [1,2]. One of the most biologically active cores is a six-membered ring containing nitrogen and sulfur atoms in positions 1 and 4 [3]. Among the types of compounds possessing a 1,4-thiazine ring, a dibenzothiazine ring system is most widely used in medicinal chemistry as a phenothiazine. Phenothiazines have been known as the largest group of antipsychotic drugs for decades [3,4]. 

For some time, a considerable amount of interest has been paid towards studying phenothiazines for qualities other than their antipsychotic activity. They were found to be good targets for drug repurposing because of their proven safety, inexpensiveness, quality, and well-known ways of synthesis [4,5,6]. Among neuroleptic phenothiazines, thioridazine is considered most promising in the treatment of multidrug-resistant tuberculosis [7,8,9] and cancer cells [10,11].

As the classical phenothiazines represent dibenzothiazines with dialkylaminoalkyl substituents at the thiazine nitrogen atom, modification of the structure led to finding new substituents and replacing one or two benzene rings with an azine ring. New phenothiazines exhibit a wide range of biological activities such as anticancer, antiviral, anti-inflammatory, immunosuppressive, and antibacterial activities, as well as the reversal of multidrug resistance discussed in the review papers and chapters [12,13,14,15,16,17,18,19], and are regarded as potential candidates in the treatment of Creutzfeldt–Jakob, Alzheimer’s, Parkinson’s, and Huntington’s diseases [20,21].

Contrary to the dibenzothiazine core, the quinoline scaffold has been known for almost 4 centuries in natural compounds such as quinine and quinidine (alkaloids from the bark of cinchona trees), which exhibit antimalarial activity [22]. Other antimalarial quinoline compounds are of synthetic origin and are known as very effective drugs such as chloroquine, amodiaquine, and primaquine [23,24]. The most effective and widely used drugs in present-day anti-infective treatment are fluoroquinolone drugs such as ciprofloxacine and ofloxacine [25,26]. The quinoline scaffold has been also found in compounds showing anticancer, antimycobacterial, anticonvulsant, anti-inflammatory, cardiovascular, and antileishmanial activities [24,27].

Replacing the benzene rings in the phenothiazine core with the quinoline ring leads to various types of quinobenzothiazines and diquinothiazines. Linearly and angularly condensed quinobenzothiazines showed promising biological activities, mainly antiproliferative, anticancer [28,29,30,31], antimicrobial [32,33,34,35,36,37], and antioxidant [38,39] activity, as well as inhibitory activity of the mitogen-induced proliferation of human peripheral blood mononuclear cells, tumor necrosis factor alpha (TNFα) production in human whole blood cultures [29,30,40], and butyrylcholinesterase inhibitory activity [41,42]. One of the linearly condensed quinobenzothiazines, 9-chloro-6-acetylaminobutylquinobenzothiazine, demonstrated therapeutic potential in the amelioration in mouse models of inflammatory symptoms of carrageenan-induced foot pads [43], imiquimod-induced psoriasis [44], contact sensitivity to oxazolone [45], and pathological changes associated with chemically induced colitis [46].

Diquinothiazines have been less explored biologically, probably because of their multistep synthesis. N-substituted diquinothiazines exhibit strong action against tens of cancer cells derived from leukemia as well as CNS, melanoma, colon, ovarian, breast, renal, prostate, skin, and non-small cell lung cancers [29,30,40,47,48], whereas NH-diquinothiazines show strong antioxidant activity [38]. The most promising diquinothiazine, 6-chloroethylureidoethyldiquinothiazine, exerted suppressive and anti-inflammatory activities in the abovementioned in vivo models [43,44,45,46], and showed inhibitory activity of IFNβ expression and IFNβ-dependent downstream genes and proteins involved in the pathogenesis of autoimmune diseases [49].

Those diquinothiazines are linearly or one-sidedly angularly condensed azaphenothiazines. The aim of this study is to report the synthesis of double-angularly condensed diquinothiazines with aminoalkyl, amidoalkyl, sulfonamidoalkyl, and substituted phenyl groups and the evaluation of their anticancer activity. 

## 2. Results

### 2.1. Chemistry

**Scheme 1:**
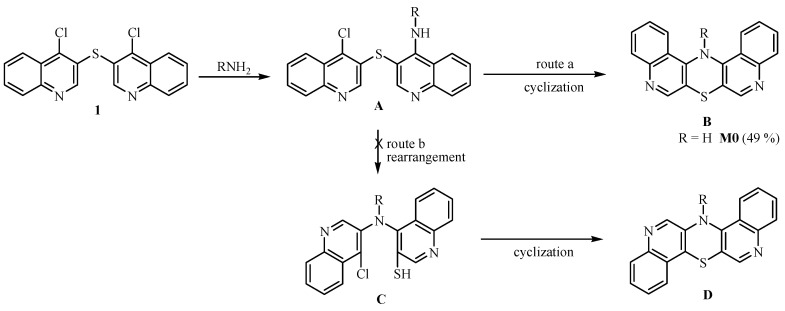
Possible reaction routes of 4,4′-dichloro-3,3′-diquinolinyl sulfide **1** with amines.

Most syntheses of phenothiazine and azaphenothiazine ring systems proceeded through the Smiles rearrangement of appropriate aryl or azinyl sulfides [3,50,51,52,53]. Reaction of 4,4′-dichloro-3,3′-diquinolinyl sulfide **1** with amines is considered to run via nucleophilic monosubstitution of one chlorine atom to form sulfide **A** (Scheme 1). This sulfide can undergo 1,4-thiazine ring closure to diquinothiazine **B** (route a), or the Smiles rearrangement to amine **C** (route b) and further ring closure to diquinothiazine **D**. Reactions of sulfide **1** with selected primary arylamines, dialkylaminoalkylamines, and alkylamines in monomethyl ether of diethylene glycol (MEDG) or in phenol led mainly to 14-substituted diquinothiazines **B.** The yields of these reactions depended mainly on the basicity and structural features of the amines. In no cases did we observe a stage of the S-N type of the Smiles rearrangement to substituted 3,4’-diquinolinylamines **C** or further to diquinothiazine **D** [54]. Both diquinothiazines **B** and **D** represent a double-angularly condensed ring system. Their discrimination is quite easy, because structure **B** shows only five aromatic proton signals in the ^1^H NMR spectrum. Contrary to the cyclization of 2,2′- [55,56,57,58,59], 2,4′- [60,61,62], and 4,4′-dipyridinyl sulfides [63], the cyclization of 3,3′-diquinolinyl sulfide **1** runs without the Smiles rearrangement.

**Scheme 2:**
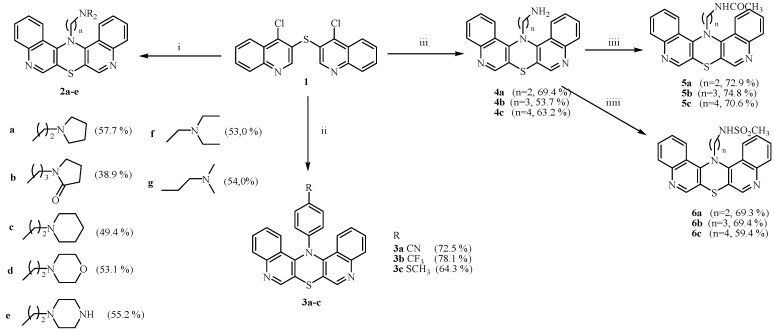
Synthesis of diquinothiazines **2–6**: (i) H_2_N(CH_2_)_n_NR_2_, phenol, 180 °C, 0.5 h; (ii) 4-substituted anilines, MEDG, reflux, 3 h; (iii) H_2_N(CH_2_)_n_NH_2_, n = 2–4, MEDG, reflux, 1 h; (iiii) (CH_3_CO)_2_O, pyridine, r. t. 24 h; (iiiii) CH_3_SO_2_Cl, CH_2_Cl_2_/10 % aqueous Na_2_CO_3_, r. t., 24 h.

Sulfide **1** reacted with dialkylaminoalkylamines, diaminoalkanes, and substituted anilines in hot phenol (180 °C) or in boiling monomethyl ether of diethylene glycol (194 °C) to form 14-dialkylaminoalkyl-, 14-phenyl-, and 14-aminoalkyldiquinotiazines **2a–e**, **3a–c,** and **4a–c** (Scheme 2). The last compounds were acetylated and methanesulfonylated to give 14-acetamido- and 14-methanesulfonamidodiquinothiazines **5a–c** and **6a–c**. It was worth noting that those annulation reactions of sulfide **1** with amines are very rare in the phenothiazine and azaphenothiazine chemistry, and they allow the steps of formation of 14*H*-diquinothiazine and its alkylation and arylation to be avoided.

### 2.2. Anticancer Activity

#### 2.2.1. Cytotoxicity and Tissue-Specific Anticancer Activities

The in vitro 72-h MTT assay, performed on cancer HCT116 and SH-5YSY cell lines as well as on lung normal epithelial BEAS-2B, cancer H1299, and A549 cells, enabled the cell viability and IC_50_ parameters for the tested compound assessment. The IC_50_ for cell viability was described with a dose of the drug, with a 50% reduction in the whole population, when compared to the untreated controls. The obtained results allow for a comparison of different compounds, using the same or different targeted cell lines. Here, the calculated IC_50_ parameters for tested compounds are presented in Table 1. Cell survival fractions (SF), followed by the MTT 72-h viability assay for the HCT116 and SH-5YSY cells, are presented in the Supplementary File (Figure S1).

Based on the obtained results, the cytotoxicity after chemical modifications did not always improve the drug activity against cancer cell lines. The obtained results showed that the tested compounds are cytotoxic in quite low doses; the colorectal cancer HCT116 cell line did not exceed a dose of 30 µM to reach decreasing cell viability and proliferation at 50% (calculated IC_50_; Table 1).

It was found that most of the tested compounds caused a significant reduction in the viability of colorectal HCT116 and neuroblastoma SH-SY5Y cancer cell lines. Comparing the tissue-dependent responses, the results of the calculated IC_50_ value for HCT116 is lower than that of the SH-SY5Y cell line, which proves that human colorectal cancer is more sensitive to the tested compounds. The compound **3c,** with the strongest anticancer activity, is characterized by a benzene ring with a -SCH_3_ substituent attached to the main structure present in each derivative. On the other hand, compounds **5a** and **5c** showed the lowest anticancer activity, and did not have an additional benzene ring in their structure, but were characterized by a straight chain containing nitrogen, sulfur, and carbon (Table 1). More detailed cytotoxic effects are presented as particular HCT116 and SH-5YSY cell viabilities charts in the Supplementary File (Figure S1).

#### 2.2.2. Live Long-Term Microscopic Observations

**Figure 1:**
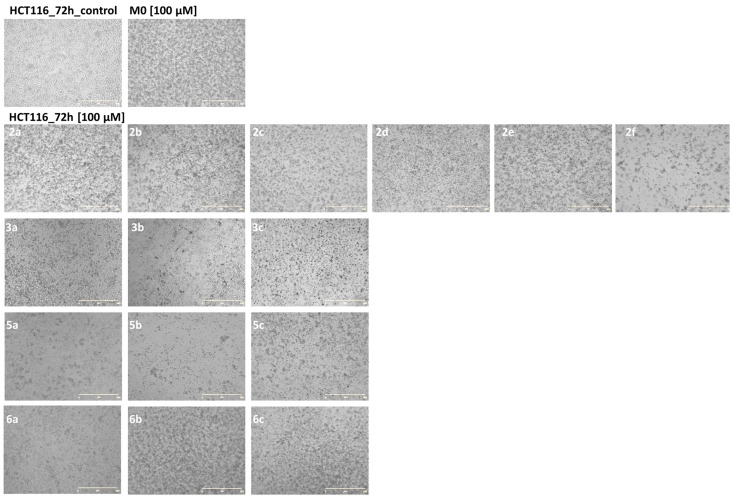
Typical images taken from HCT116 cells after 72 h of exposure to 100 µM of tested compounds. Juli Fl apparatus (NanoEntek); magnification 4× *g*; scale bar 100 µm.

**Figure 2:**
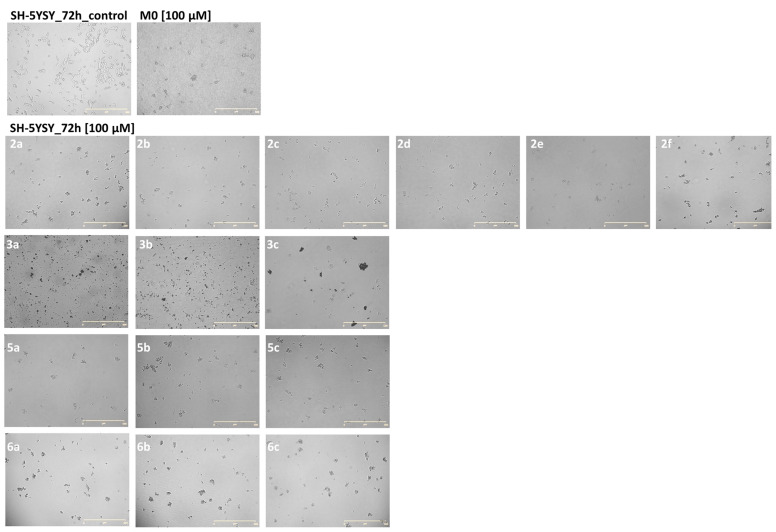
Typical images taken from SH-5YSY cells after 72 h of exposure to 100 µM of tested compounds. Juli Fl apparatus (NanoEntek); magnification 4× *g*; scale bar 100 µm.

The microscopic observations showed cell damage and typically morphological changes in comparison to the untreated controls for HCT116 cells. When tested drugs added to the medium, were more concentrated than the IC_50_ value—cells were always exposed to the dose of 100 µM for each compound (Figure 1). The parent molecule **M0** was also tested for more than IC_50_ effective dose, as well as with the viability and microscopic observation assays it was 100 µM. The neuroblastoma SH-5YSY cells were surprisingly not sensitive to the tested compounds when resuming the viability MTT assay (Table 1 and Supplementary Figure S1); in fact, they seemed to be more chemosensitive to the tested compounds. Rather, the microscopic observations confirm the cytostatic action, with cell proliferation significantly reduced and cellular death induced in SH-5YSH (Figure 2).

#### 2.2.3. Proapoptotic and Cell Cycle Cytostatic Action

**Figure 3:**
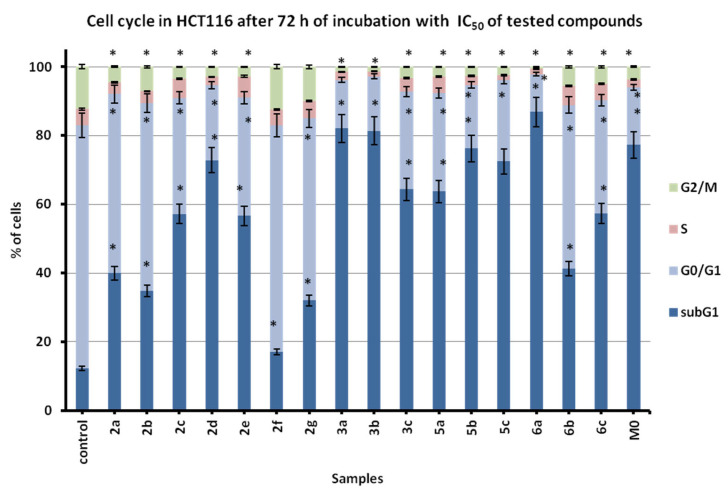
Cell cycle distribution in HCT116 cells after 72 h of incubation with doses of IC_50_ or 100 µM, respectively. SubG1 fraction represents apoptotic and dead cells. Results presented as mean from 3 experiments, +/− SD. Statistical significance indicated by *; evaluated by *t*-test, where *p* < 0.05.

**Figure 4:**
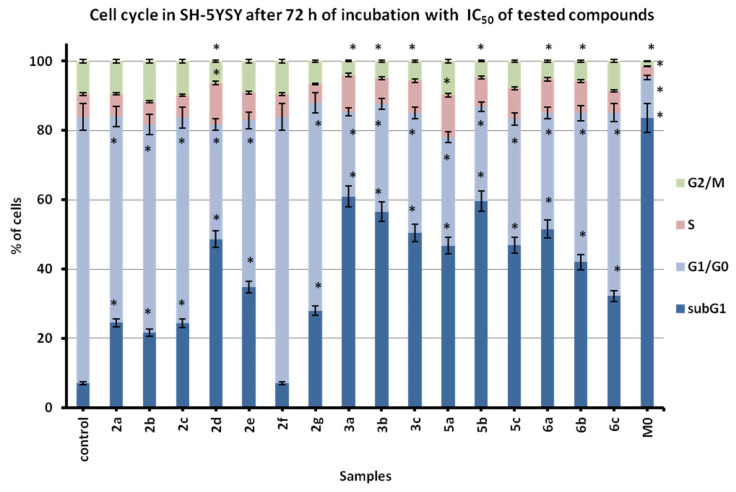
Cell cycle distribution in SH-5YSY cells after 72 h of incubation with doses of IC_50_ or 100 µM, respectively. SubG1 fraction represents apoptotic and dead cells. Results presented as mean from 3 experiments, +/− SD. Statistical significance indicated by * ; evaluated by *t*-test, where *p* < 0.05.

The losing of action against neuroblastoma cells was also confirmed during cytometric cell cycle evaluation, where the proapoptotic action was more visible in the colorectal HCT116 (Figure 3) than in the neuroblastoma SH-5YSY cell line (Figure 4). All findings assume the mode of action of modified derivatives, with special focus on the origin of cancer cells, where specific membrane transporters are required for a full spectrum of action development. It is possible that the cellular death, observed on microscopic live and long-term observations, is caused by the derivative’s lower solubility and the modification of the parameters of the cellular environment.

The subG1 fraction, presented on typical flow cytometric histograms (Supplementary Figures S2 and S3), resulted in damage and death in apoptotic and necrotic cells, both in colorectal HCT116 and neuroblastoma SH-5YSY cell lines. Cell cycle distribution in HCT116 cells indicates proapoptotic action (Figure 3). In addition, in SH-5HSY, cytostatic activity with some inhibition of the cell cycle in the S phase (connected to the DNA replication arrest and nucleic acid damage) was observed. Proapoptotic action in both cancer cell lines is obvious, but cytostatic action against neuroblastoma cells resulted in lethal effects being visible on live microscopic observations.

## 3. Materials and Methods

### 3.1. Chemistry

Melting points were determined in open capillary tubes on a Boetius melting point apparatus, and were uncorrected. The NMR spectra were recorded on Bruker Fourier 300 and Bruker Avance spectrometers (^1^H at 300 and 600 MHz) in CDCl_3_. HR MS mass spectra were run on an Impact II spectrometer. 4,4′-Dichloro-3,3′-diquinolinyl sulfide was obtained from diquinodithiin according to the reported procedure [64,65].

Synthesis of 14-dialkylaminoalkyldiquinothiazines **2**

To a melted phenol (1.0 g), 4,4′-dichloro-3,3′-diquinolinyl sulfide **1** (180 mg, 0.5 mmol) was added and heated to 180 °C with stirring. When the temperature reached 180 °C N,N-dialkylaminoalkylamine was added (1.25 mmol, 1-(2-aminoethyl)pyrrolidine, 1-(3-aminopropyl)-2-pyrrolidinone, 1-(2-aminoethyl)piperidine, 4-(2-(aminoethyl)morpholine, 4-(2-aminoethyl)piperazine), and the mixture was stirred for 0.5 h. After that, an additional portion of N,N-dialkylaminoalkylamine was added (1.25 mmol) and the mixture was stirred for another 0.5 h. After cooling to room temperature, phenol was extracted with 5% aqueous NaOH (3 × 10 mL). The resulting residue was washed with water (10 mL), dried, and purified using column chromatography (SiO_2_, CHCl_3_, and CHCl_3_-EtOH 100:1–100:8) to give the following:

14-(pyrrolidinylethyl)diquinothiazine **2a** (115 mg, 57.7%), an oil. ^1^H NMR, CDCl_3_ δ: 1.51 (s, 4H, 2CH_2_), 2.22 (s, 4H, 2NCH_2_), 2.72 (t, 2H, NCH_2_), 4.20 (t, 2H, NCH_2_), 7.61 (t, 2H, H-2, H-12), 7.66 (t, 2H, H-3, H-11), 8.07 (d, 2H, H-4, H-10), 8.30 (d, 2H, H-1, H-13), 8.60 (s, 2H, H-6, H-8). ^13^C NMR (CDCl_3_) δ: 23.25 (2CH_2_), 54.02 (4C-NCH_2_, 2C-CH_2_N), 122.04 (C-1, C-13), 125.74 and 127.49 (9a and 4a; 16a and 17a), 129.49 (C-2, C-12), 129.54 (C-3, C-11), 130.30 (C-4, C-10), 148.31 (C-6, C-8), 149.09 (C-13b, C-14a). HR MS: Calcd. for C_24_H_23_N_4_S^+^: 399.1643, found: 399.1641.

14-(pyrrolidinonylpropyl)diquinothiazine **2b** (83 mg, 38.9%), an oil. ^1^H NMR, CDCl_3_ δ: 1.69 (m, 2H, CCH_2_C), 1.89 (m, 2H, CCH_2_C), 2.16 (t, 2H, COCH_2_), 2.93 (t, 2H, NCH_2_), 3.10 (t, 2H, NCH_2_), 4.07 (t, 2H, NCH_2_), 7.65 (t, 2H, H-2, H-12), 7.72 (t, 2H, H-3, H-11), 8.10 (d, 2H, H-4, H-10), 8.28 (d, 2H, H-1, H-13), 8.64 (s, 2H, H-6, H-8). ^13^C NMR (CDCl_3_) δ: 17.58, 27.13, 30.70, 39.68, 46.60, 55.78, 122.13 (C-1, C-13), 123.86 (C-13a, C-14b), 125.40 (C-6a, C-7a), 127.84 (C-4a, C-9a), 129.68 (C-2, C-12), 130.00 (C-4, C-10), 130.06 (C-3, C-11), 147.51 (C-6, C-8), 148.20 (C-13b, C-14a). HR MS: Calcd. for C_25_H_23_N_4_OS^+^: 427.1593, found: 427.1593.

14-(piperidinylethyl)diquinothiazine **2c** (102 mg, 49.4%), an oil. ^1^H NMR, CDCl_3_ δ: 1.20 (s, 6H, 3CH_2_), 2.11 (s, 4H, 2NCH_2_), 2.56 (t, 2H, NCH_2_), 4.23 (t, 2H, NCH_2_), 7.65 (t, 2H, H-2, H-12), 7.70 (t, 2H, H-3, H-11), 8.10 (d, 2H, H-4, H-10), 8.33 (d, 2H, H-1, H-13), 8.64 (s, 2H, H-6, H-8). ^13^C NMR (CDCl_3_) δ: 54.49 (7C-CH_2_), 121.95 (C-1, C-13), 123.94 (C-4a, C-9a, C-13a, C-14b), 125.77 (C-6a, C-7a), 127.73 (C-2, C-12), 129.64 (C-3, C-11), 130.35 (C-4, C-10), 148.26 (C-6, C-8), 148.20 (C-13b, C-14a). HR MS: Calcd. for C_25_H_25_N_4_S^+^: 413.1800, found: 413.1800. 

14-(morpholinylethyl)diquinothiazine **2d** (110 mg, 53.1%), an oil. ^1^H NMR, CDCl_3_ δ: 2.13 (s, 4H, 2CH_2_), 2.57 (t, 2H, NCH_2_), 3.24 (s, 2H, NCH_2_), 4.20 (t, 2H, NCH_2_), 7.64 (t, 2H, H-2, H-12), 7.70 (t, 2H, H-3, H-11), 8.10 (d, 2H, H-4, H-10), 8.30 (d, 2H, H-1, H-13), 8.63 (s, 2H, H-6, H-8). ^13^C NMR (CDCl_3_) δ: 53.30 (6C-CH_2_), 121.90 (C-1, C-13), 123.91 (C-13a, C-14b), 125.77 (C-6a, C-7a), 127.39 (C-4a, C-9a), 128.00 (C-2, C-12), 129.55 (C-4, C-10), 130.06 (C-3, C-11), 148.26 (C-6, C-8), 148.20 (C-13b, C-14a). HR MS: Calcd. for C_24_H_23_N_4_OS^+^: 415.1593, found: 415.1598.

14-(piperazinyloethyl)dichinotiazyny **2e** (114 mg, 55.2%), an oil. ^1^H NMR, CDCl_3_: 2.39 (broad s, 1H, NH), 2.53 (t, 4H, 2CH_2_), 4.17 (t, 4H, 2CH_2_), 7.61 (t, 2H, H-2, H-12), 7.67 (t, 2H, H-3, H-11), 8.07 (d, 2H, H-4, H-10), 8.28 (d, 2H, H-1, H-13), 8.61 (s, 2H, H-6, H-8). ^13^C NMR (CDCl_3_) δ: 52.02 (6C-CH2), 122.04 (C-1, C-13), 124.01 (C-13a, C-14b), 125.7 (C-6a, C-7a), 128.49 (C-4a, C-9a), 129.20 (C-2, C-12), 129.59 (C-4, C-10), 130.24 (C-3, C-11), 147.60 (C-6, C-8), 148.51 (C-13b, C-14a). HR MS: Calcd. for C_24_H_24_N_5_S^+^: 414.1752, found: 414.1752.

Synthesis of 14-(4-substituted phenyl)diquinothiazines **3a–c**

To a solution of 4,4′-dichloro-3,3′-diquinolinyl sulfide (90 mg, 0.25 mmol) in monomethyl ether of diethylene glycol (MEDG, 3 mL), 4-substituted anilines (0.75 mmol, 4-cyanoaliniline, 4-trifluoromethylaniline, 4-methylthioaniline) were added, and the reaction mixture was boiled for 3 h. After cooling to room temperature, the reaction mixture was poured in water (7.5 mL) and left out overnight. The resulting solid was filtered off and crystallized from MEDG to give the following: 

14-(4-cyanophenyl)diquinothiazine **3a** (73 mg, 72.5%), mp >300 °C. ^1^H NMR, CDCl_3_ δ: 6.52 (d, 2H_arom_), 7.37 (d, 2H_arom_), 7.76 (t, 2H, H-2, H-12), 7.86 (t, 2H, H-3, H-11), 8.28 (d, 2H, H-4, H-10), 8.29 (d, 2H, H-1, H-13), 9.11 (s, 2H, H-6, H-8). ^13^C NMR (CDCl_3_) δ: 104.69, 115.07, 118.89, 122.38, 126.16, 128.98, 130.27, 130.59, 133.43, 143.93, 147.53, 147.88, 149.46. HR MS: Calcd. for C_25_H_15_N_4_S^+^: 403.1017, found: 403.1009.

14-(4-trifluoromethylphenyl)diquinothiazine **3b** (87 mg, 78.1%), mp 292–293 °C. ^1^H NMR, CDCl_3_ δ: 6.54 (d, 2H_arom_), 7.33 (d, 2H_arom_), 7.74 (t, 2H, H-2, H-12), 7.84 (t, 2H, H-3, H-11), 8.28 (d, 2H, H-4, H-10), 8.33 (d, 2H, H-1, H-13), 9.10 (s, 2H, H-6, H-8). ^13^C NMR (CDCl_3_) δ: 114.62, 115.09, 122.65, 126.44, 126.51, 128.80, 129.93, 130.21, 130.49, 143.93, 147.76, 148.55, 149.40. HR MS: Calcd. for C_25_H_15_F_3_N_3_S^+^: 446.0939, found: 446.0933.

14-(methylthiophenyl)diquinothiazine **3c** (68 mg, 64.3%), mp 262–263 °C. ^1^H NMR, CDCl_3_ δ: 2.37 (s, 3H, SCH_3_), 6.43 (d, 2H_arom_), 7.02 (d, 2H_arom_), 7.72 (t, 2H, H-2, H-12), 7.82 (t, 2H, H-3, H-11), 8.26 (d, 2H, H-4, H-10), 8.38 (d, 2H, H-1, H-13), 9.06 (s, 2H, H-6, H-8). ^13^C NMR (CDCl_3_) δ: 16.57, 115.60, 116.03, 122.79, 123.19, 125.42, 126.72, 128.45, 129.99, 132.50, 140.97, 143.99, 147.19, 147.99, 149.48. HR MS: Calcd. for C_25_H_18_N_3_S_2_^+^: 424.0942, found: 424.0930.

Synthesis of 14-aminoalkyldiquinothiazines **4a–c**

To a solution of 4,4′-dichloro-3,3′-diquinolinyl sulfide (100 mg, 0.28 mmol) in monomethyl ether of diethylene glycol (MEDG, 3 mL), 1,ω-diaminoalkane (1.5 mmol, 1,2-diaminoethane, 1,3-diaminopropane, 1,4-diaminobutane) was added, and the reaction mixture was boiled for 1 h. After cooling to room temperature, the reaction mixture was poured in water (50 mL) and left out overnight. A resulting solid was filtered off, washed with water, and purified by preparative thin-layer chromatography (silica gel, CHCl_3_-EtOH 5:1) to obtain the following:

14-aminoethyldiquinothiazine **4a** (67 mg, 69.4%), mp 196–197 °C. ^1^H NMR, CDCl_3_ δ: 2.88 (t, 2H, CH_2_), 4.26 (t, 2H, CH_2_), 7.69 (t, 2H, H-2, H-12), 7.74 (t, 2H, H-3, H-11), 8.13 (d, 2H, H-4, H-10), 8.33 (d, 2H, H-1, H-13), 8.68 (s, 2H, H-6, H-8). HR MS: Calcd. for C_20_H_16_N_4_S^+^: 345.1174, found: 345.1171. 

14-aminopropyldiquinothiazine **4b** (54 mg, 53.7%), mp 134–136 °C. ^1^H NMR, CDCl_3_ δ: 1.94 (m, 2H, C-CH_2_-C), 3.38 (t, NCH_2_), 4.41 (t, NCH_2_), 7.82 (t, 2H, H-2, H-12), 7.89 (t, 2H, H-3, H-11), 8.34 (d, 2H, H-4, H-10), 8.41 (d, 2H, H-1, H-13), 8.74 (s, 2H, H-6, H-8). HR MS: Calcd. for C_21_H_18_N_4_S^+^: 359.1330, found: 359.1314. 

14-aminobutyldiquinothiazine **4c** (66 mg, 63.2%), mp 77–78 °C. ^1^H NMR, CDCl_3_ δ: 1.43 (m, 2H, C-CH_2_-C), 1.62 (m, 2H, C-CH_2_-C), 3.12 (t, 2H, NCH_2_), 4.22 (t, 2H, NCH_2_), 7.69 (t, 2H, H-2, H-12), 7.72 (t, 2H, H-3, H-11), 8.09 (d, 2H, H-4, H-10), 8.24 (d, 2H, H-1, H-13), 8.76 (s, 2H, H-6, H-8). HR MS: Calcd. for C_22_H_20_N_4_S^+^: 373.1487, found: 373.1470. 

Synthesis of 14-acetylaminoalkyldiquinothiazines **5**

To a solution of 14-aminoalkyldiquinothiazine (14-aminoethyldiquinothiazine **4a**, 96 mg, 0.28 mmol; 14-aminopropyldiquinothiazine **4b**, 107 mg, 0.3 mmol; 14-aminobutyldiquinothiazine **4c**, 108 mg, 0.29 mmol) in pyridine (1.5 mL), acetyl anhydride (0.9 mL, 9.5 mmol) was added drop-by-drop and the mixture was stirred for 24 h. After that, the mixture was poured into water (20 mL) and extracted with methylene chloride (20 mL). The solvent was distilled off and the residue was purified using column chromatography (Al_2_O_3_, CHCl_3_ and EtOH, 5:1) to give the following:

14-acetylaminoethyldiquinothiazine **5a** (79 mg, 72.9%), mp 89–90 °C. ^1^H NMR (CDCl_3_) δ: 1.63 (s, 3H, CH_3_), 3.33 (q, 2H, NHCH_2_), 4.46 (t, 2H, CH_2_), 6.27 (broad s, 1H, NH), 7.73 (t, 2H, H-2, H-12), 7.79 (t, 2H, H-3, H-11), 8.20 (d, 2H, H-4, H-10), 8.27 (d, 2H, H-1, H-13), 8.72 (s, 2H, H-6, H-8). ^13^C NMR (CDCl_3_) δ: 22.71, 43,09, 56.18, 122.35, 123.79, 124.01, 127.55, 129.87, 130.12, 131.32, 147.87, 148.79, 169.61. HR MS: Calcd. for C_22_H_18_N_4_OS^+^: 387.1280, found: 387.1280.

14-acetylaminopropyldiquinothiazine **5b** (90 mg, 74.8%), mp 171–172 °C. ^1^H NMR, CDCl_3_ δ: 1.79 (s, 3H, CH_3_), 1.90 (m, 2H, C-CH_2_-C), 3.14 (q, 2H, NHCH_2_), 4.16 (t, 2H, NCH_2_), 5.34 (broad s, 1H, NH), 7.70 (t, 2H, H-2, H-12), 7.76 (t, 2H, H-3, H-11), 8.16 (d, 2H, H-4, H-10), 8.32 (d, 2H, H-1, H-13), 8.67 (s, 2H, H-6, H-8). ^13^C NMR (CDCl_3_) δ: 13.04, 21.96,27.89, 43.09, 122.41, 123.51, 123.67, 127.10, 127.88, 130.02, 131.34, 148.78, 149.68, 169.43. HR MS: Calcd. for C_23_H_20_N_4_OS^+^: 401.1436, found: 401.1438. 

14-acetylaminobutyldiquinothiazine **5c** (85 mg, 70.6%), mp 68–69 °C. ^1^H NMR (CDCl_3_) δ: 1.36 (m, 2H, C-CH_2_-C), 1.69 (m, 2H, C-CH_2_-C), 1.84 (s, 3H, CH_3_), 3.06 (q, 2H, NHCH_2_), 4.10 (t, 2H, NCH_2_), 5.34 (broad s, 1H, NH), 7.69 (t, 2H, H-2, H-12), 7.74 (t, 2H, H-3, H-11), 8.13 (d, 2H, H-4, H-10), 8.32 (d, 2H, H-1, H-13), 8.68 (s, 2H, H-6, H-8). ^13^C NMR (CDCl_3_) δ:22.79, 29.32, 38.11, 43.11, 56.32, 122.25, 122.59, 124.00, 127.25, 129.91, 130.13, 131.10, 147.83, 148.80, 170.37. HR MS: Calcd. for C_24_H_22_N_4_OS^+^: 415.1593, found: 415.1591. 

Synthesis of 14-methanesulfonylaminoalkyldiquinothiazines **6**

To a stirring mixture of 14-aminoalkyldiquinothiazine (14-aminoethyldiquinothiazine **4a**, 103 mg, 0.3 mmol; 14-aminopropyldiquinothiazine **4b**, 107 mg, 0.3 mmol; 14-aminobutyldiquinothiazine **4c**, 93 mg, 0.25 mmol), methylene chloride (3 mL) and 10% aqueous Na_2_CO_3_ (4 mL) at room-temperature methanesulfonyl chloride (0.36 mL, 4.7 mmol or 0.3 mL, 3.9 mmol in the case of the last substrate) were added-drop by-drop, and the mixture was stirred for 24 h. After that, the mixture was separated and the aqueous layer was washed with chloride methylene (2 × 5 mL). The combined organic extracts were washed with water (10 mL), dried over Na_2_SO_4_, and distilled off. The residue was purified using column chromatography (Al_2_O_3_, CHCl_3_ and EtOH, 5:1) to give the following:

14-methanesulfonylaminoethyldiquinothiazine **6a** (88 mg, 69.3%), mp 229–230 °C. ^1^H NMR CDCl_3_ δ: 2.70 (s, 3H, CH_3_), 3.29 (q, 2H, NHCH_2_), 4.52 (t, 2H, NCH_2_), 4.52 (broad s, 1H, NH), 7.56 (t, 2H, H-2, H-12), 7.81 (t, 2H, H-3, H-11), 8.22 (d, 2H, H-4, H-10), 8.30 (d, 2H, H-1, H-13), 8.72 (s, 2H, H-6, H-8). ^13^C NMR (CDCl_3_) δ: 39.21, 43.09, 55.77, 121.21, 122.52, 123.68, 124.22, 127.06, 129.87, 130.14, 147.68, 148.80. HR MS: Calcd. for C_21_H_18_N_4_O_2_S_2_^+^: 423.0949, found: 423.0946. 

14-methanesulfonylaminopropyldiquinothiazine **6b** (91 mg, 69.4%), mp 199–200 °C. ^1^H NMR, CDCl_3_ δ: 1.96 (m, 2H, C-CH2-C), 2.75 (s, 3H, CH_3_), 3.09 (q, 2H, NHCH_2_), 4.29 (t, 2H, NCH_2_), 4.55 (broad s, 1H, NH), 7.73 (t, 2H, H-2, H-12), 7.76 (t, 2H, H-3, H-11), 8.16 (d, 2H, H-4, H-10), 8.33 (d, 2H, H-1, H-13), 8.66 (s, 2H, H-6, H-8). ^13^C NMR (CDCl_3_) δ: 28.68, 38.60, 54.97, 121.29, 122.57, 123.78, 124.32, 126.96, 127.50, 130.08, 147.79, 148.67. HR MS: Calcd. for C_22_H_20_N_4_O_2_S_2_^+^: 437.1106, found: 437.1105. 

14-methanesulfonylaminobutyldiquinothiazine **6c** (67 mg, 59.4%), mp 184–186 °C. ^1^H NMR, CDCl_3_ δ: 1.46 (m, 2H, C-CH_2_-C), 1.74 (m, 2H, C-CH_2_-C), 2.81 (s, 3H, CH_3_), 2.96 (q, 2H, NHCH_2_), 4.16 (t, 2H, NCH_2_), 4.44 (broad s, 1H, NH), 7.71 (t, 2H, H-2, H-12), 7.77 (t, 2H, H-3, H-11), 8.19 (d, 2H, H-4, H-10), 8.32 (d, 2H, H-1, H-13), 8.69 (s, 2H, H-6, H-8). ^13^C NMR (CDCl_3_) δ: 26.78, 38.69, 39.62, 49.18, 55.01, 121.18, 122.76, 123.77, 124.49, 126.92, 127.58, 130.01, 148.89, 149.87. HR MS: Calcd. for C_23_H_22_N_4_O_2_S_2_^+^: 451.1262, found: 451.1260. 

### 3.2. Biological Evaluation

#### 3.2.1. Cell Culturing

For cytological studies, human cancer cell lines were chosen, with special emphasis on the colorectal, HCT116, and neuroblastoma SH-5YSY cell lines, as well as lung normal epithelial BEAS-2B and cancer H1299 and A549 cancer cell lines (all obtained from the ATTC collection; Manassas, VA, USA). The cells were harvested under the standard conditions in incubator (5% CO_2_, 37 °C and 60% humidity; Panasonic model MCO-19 AIC). Cells were cultured in T75 mL sterile bottles (Sartedt), using DMEM-F12 medium (Merck, Poznań, Poland) supplemented with 10% of Fetal Bovine Serum (EURx, Gdańsk, Poland). During the experimental procedure, each cell line wasn’t more than 60–80% confluenced, no older than maximal number of passages 30. For adherent cell passages, the enzyme trypsin working solution, prepared in sodium phosphate buffer saline (PBS, pH = 7.4; PAN-Biotech Gmbh, Aidenbach, Germany), was used. The tested compounds were prepared as 1 mM stocks in 100% DMSO (Merck, Poznań, Poland), and were stored at −20 °C. Before use in cytological studies, the working solution of each compound was prepared directly in complete DMEM-F12 medium. A positive-control, anticancer drug, etoposide (Merck, Poznań, Poland), was dissolved and prepared as solutions with doses of 20, 10, 5, 2.5, 1.25, 0.63 µM (0 µM for control, untreated cells in DMEM-F12 without any tested compound additions), respectively.

#### 3.2.2. Viability MTT Assay

HCT116 and SH-5YSY cells collected after trypsinization were counted under the Bürker’s chamber, and 2000 or 5000 cells were seeded on each well of the 96-well-format sterile plate (Sarstedt, Nümbrecht, Germany), in 100 µL of complete DMEM-F12 medium, respectively. Twenty-four hours before compound addition, the cells were monitored directly on plates; only well-attached cells were used for treatments with background controls (DMSO with final maximal concentration of 1%) and untreated controls. The normal lung (BEAS-2B) and cancer (H1299 and A449) cell lines were cultured and prepared for viability MTT assay in similar way. To treat wells, 100 µL of tested compounds in fresh medium were added at concentration range of 100, 50, 25, 12.5, 6.25, 3.13 µM (0 µM for control, untreated cells in DMEM-F12 without any tested compound additions). An etoposide as a positive control, as anticancer drug, was prepared in similar way. Followed by 72 h of incubation, the viability assay was performed according to the producer protocol (Promega, Madison, WI, USA), where the medium and compounds were removed and cells were washed with PBS solution. A yellow tetrazolium salt (3-(4,5-dimethylthiazol-2-yl)-2,5-diphenyltetrazolium bromide solution (0.5 mg/mL) in serum and phenol red-free DMEM (Merck, Poznań, Poland) was added to each well on plates at a volume of 50 µL for 2–3 h of darkness, at 37 °C incubation. The purple formazan crystals were produced only by the viable cells, containing NAD (P)H-dependent oxidoreductase enzymes, which reduce the MTT. The crystals were dissolved in acidic (0.04 M HCl) 2-propanolol solution (Merck, Poznań, Poland) after addition of 75 µL of dissolving mixture to each well on plates for 5–10 min in darkness and at room-temperature (RT) incubation. The absorption at 570 nm was measured directly using multiplate reader (Epoch, BioTek, Winooski, VT, USA). Each time, the biological experiments were repeated three times.

#### 3.2.3. Long-Term Life Microscopic Observations

Live observations were performed on colorectal HCT116, and neuroblastoma SH-5YSY cell lines, using JuLI_FL™ apparatus (NanoEntek, Waltham, MA, USA). The cells were cultured on 3 cm diameter Petri dishes (Beckton Dickinson, Franklin Lakes, NJ, USA), with an initial 10,000 cells in 2 mL of fresh DMEM-F12 medium as well as untreated control, and exposed to 100 µM solution of tested compounds. The control and tested cells were observed within 72 h directly in the transit channel, to monitor cell proliferation. Finally, the images were recorded by the automated analyzer in the transit channel for cell morphology and confluence detection. Image processing was conducted according to the procedure based on automatic live observations tutorial, with JuliTmSTAT Cell analysis software, version 2.0.1.0 by NanoEnTek Inc. (Seoul, Republic of Korea).

#### 3.2.4. Flow Cytometry Cell Cycle and Apoptosis Evaluation

For cellular apoptotic dead cells and cell cycle estimations, the cell cultures were plated in 6-well plates at a confluence of 3 × 10^5^ cells in 2 mL of complete fresh DMEM-F12 medium. After 24 h, the medium was replaced with prepared sample solutions, at doses calculated from MTT assay IC_50_ values, or 100 μM for ineffective derivatives, for next long-term 72 h incubations, respectively. After incubation, the collected cells were centrifuged, and the pellet was dissolved in 250 μL of hypotonic buffer (hypotonic buffer comprised from PI 100 μg/mL in PBS (BD Biosciences, San Jose, CA, USA); 5 mg/L of citric acid; 1:9 Triton-X solution; RNase 100 μg/mL in PBS (Sigma, Poznań, Poland)). The samples were incubated for 15 min at room temperature and in darkness. The cellular DNA contents were determined by fluorescence measurements using BD FACS Aria™ III sorter (Becton, Dickinson and Company, Franklin Lakes, NJ, USA), using PE configuration (547 nm excitation laser line; emission: 585 nm). At least 1 × 10^4^ cells were analyzed for each sample, recording the DNA content as a differentiating parameter for mononuclear cells (G0/G1 phase); S (DNA replication phase); G2/M (binucleated and mitotic fraction) or dead, necrotic, and apoptotic cells (subG1 fraction), respectively. The results were analyzed using the free software Flowing Software 2.5.1 (Perttu Terho, Turku Centre for Biotechnology, University of Turku, Finland) and are presented as mean fluorescence.

#### 3.2.5. Statistical Analysis

Obtained absorbance was analyzed for proliferation and survival fraction (SF) assessments, where tested samples were compared to the untreated controls (100%). Using Excel software (Microsoft Office 2010), an IC_50_ index was calculated for tested compounds/drugs, where used concentration reduced viability of treated populations in 50% in comparison to the untreated controls. The results from MTT and cell cycle are presented as mean from three experiments and +/− SD was added. The statistical significance was calculated with *t*-test and indicated on charts by a star (*p* value < 0.05).

## 4. Conclusions

In this study, we report the synthesis of double-angularly condensed diquinothiazines with aminoalkyl, amidoalkyl, sulfonamidoalkyl, and substituted phenyl groups and the evaluation of their anticancer activity. The titular diquinothiazines were obtained via annulation reactions of 4,4′-dichloro-3,3′-diquinolinyl sulfide **1** with dialkylaminoalkylamines (**2a–g**), diaminoalkanes (**4a–c**), and substituted anilines (**3a–c**). The compounds **4a–c** were acylated and methanesulfonylated to give 14-acetamido- and 14-methanesulfonamidodiquinothiazines **5a–c** and **6a–c**. The analysis of ^1^H and ^13^C NMR spectra allowed us to clearly state that the reaction of the 1,4-thiazine ring formation in 14-substituted diquinothiazines does not proceed with the Smiles rearrangement. Among them, compounds with aminoalkyl and phenyl groups showed excellent broad-spectrum anticancer activity. Three of the tested diquinothiazines (**2c**, **3a**, **and 3c**) showed better activity against the HCT116 (IC_50_ < 8.6 µM) cell line than etoposide used as a reference drug. However, against the SH-SY5Y cell line, diquinothiazines **M0**, **2f**, **2g**, **3a**, **3b**, **6a**, and **6b** turned out to be inactive in the concentration range studied. The lowest activity of the new diquinothiazymes was found against the H1299 cell line. The **3c** derivative with a methylthiophenyl substituent turned out to be the most active against all cell lines used in the study with IC_50_ values 1.6–17.2 µM. Furthermore, some of the diquinothiazines showed greater activity against the cell lines used in the study than etoposide and lower cytotoxicity against normal cells than this reference drug (Table 1).

Microscopic observations showed cell damage and typically morphological changes compared to the untreated controls for HCT116 cells, and rather confirm the cytostatic action, with a significant induction of cell death in SH-5YSH. The flow cytometry showed the proapoptotic effect of the compounds tested. Inhibition of the cell cycle in the S phase was observed, which is associated with nucleic acid damage and is associated with an arrest of DNA replication. Analyzing the structure–activity relationship (SAR), it can be seen that the anticancer activity depends on the type of substituent at the thiazine nitrogen atom and on the type of tested cancer lines and their sensitivity. Further advanced studies on the mechanism of anticancer activity for this group of diquinothiazines are planned.

## Figures and Tables

**Table 1:** IC_50_ calculated for HCT116, SH-SY5Y, A549, H1299, and BEAS-2B cell lines after 72 h of incubation with tested compounds (mean value from 3 experiments +/−SD).

Compound No.		IC_50_ [μM] Cell Line							
	HCT116	SI	SH-SY5Y	SI	A549	SI	H1299	SI	BEAS-2B
**M0**	5.8 ± 0.63	-	no effect	-	18.0 ± 3.09	-	67.2 ± 9.64	-	no effect
**2a**	11.1 ± 3.39	7.6	6.5 ± 1.22	13	13.3 ± 2.23	6.3	8.1 ± 1.83	10.4	84.5 ± 11.05
**2b**	9.4 ± 4.41	8.9	7.7 ± 2.59	10.8	12.4 ± 2.25	6.7	5.3 ± 1.04	15.8	83.5 ± 12.26
**2c**	2.8 ± 0.45	-	14.3 ± 1.49	-	14.7 ± 2.81	-	7.5 ± 1.97	-	no effect
**2d**	23.1 ± 1.37	5	13.5 ± 2.69	8.7	20.7 ± 2.25	5.7	8.9 ± 1.02	13.2	117.8 ± 18.45
**2e**	10.3 ± 1.82	2.5	4.9 ± 0.87	5.4	10.1 ± 2.26	2.6	7.0 ± 1.85	3.8	26.5 ± 1.85
**2f**	16.9 ± 1.73	7.2	no effect	-	27.9 ± 2.07	4.4	22.2 ± 3.83	5.5	121.5 ± 12.04
**2g**	13.1 ± 1.82	-	no effect	-	31.5 ± 2.36	-	24.5 ± 1.91	-	no effect
**3a**	4.9 ± 1.97	-	no effect	-	84.9 ± 12.09	-	3.7 ± 0.91	-	no effect
**3b**	17.1 ± 4.81	5.0	no effect	-	42.5 ± 16.53	2.0	14.6 ± 1.92	5.9	86.1 ± 10.96
**3c**	2.3 ± 0.51	30	2.7 ± 0.21	31.9	17.2 ± 1.02	4.0	1.6 ± 0.86	43.1	69.0 ± 4.89
**5a**	25.9 ± 4.87	4.1	24.7 ± 5.44	4.4	71.1 ± 14.71	1.5	26.2 ± 1.99	4.1	107.7 ± 9.83
**5b**	17.7 ± 1.53	4.9	21.4 ± 4.02	4.1	56.5 ± 15.62	1.5	23.5 ± 1.97	3.7	86.9 ± 9.67
**5c**	26.5 ± 1.15	-	21.9 ± 4.31	-	49.3 ± 15.91	-	30.3 ± 1.57	-	no effect
**6a**	20.2 ± 1.01	-	no effect	-	65.4 ± 14.14	-	36.9 ± 6.06	-	no effect
**6b**	15.6 ± 1.16	-	no effect	-	45.2 ± 17.16	-	25.9 ± 4.14	-	no effect
**6c**	17.6 ± 1.85	-	21.6 ± 7.64	-	36.9 ± 16.77	-	13.4 ± 1.68	-	no effect
**etoposide**	8.6 ± 1.42	0.0004	3.9 ± 0.76	0.001	4.48 ± 0.21	0.001	0.6 ± 0.16	0.005	0.00311 ± 0.0002

No effect—viability of cells (SF) similar to the control’s, about 100%.

## Data Availability

Data is contained within the article.

## Supplementary Materials

The following supporting information can be downloaded at: https://www.mdpi.com/article/10.3390/molecules28073248/s1, Figure S1. Survival fraction of HCT116 (left) and SH-5YSY (right) cell lines after 72 h of incubation with tested compounds and positive control, anticancer drug: etoposide, respectively, evaluated by MTT assay. Results presented as mean from 3 experiments, +/- SD. Statistical significance indicated by star; evaluated by *t*-test, where *p* < 0.05 (TRUE bolded under the charts). Figure S2. Typical histograms of cell cycle distribution in control and treated HCT116 cells after 72 h of incubation with tested compounds, at dose of 100 µM. DNA gating during cytometry analyses after iodium propide staining (PI; 100 µg/mL; [a.u.]) showed the cells in cell cycle phases: subG1; G0/G1; S and G2/M, respectively.
