# Supplementary material for: 14-Substituted Diquinothiazines as a New Group of Anticancer Agents
Source: Molecules. 2023 Apr 5;28(7):3248. doi: 10.3390/molecules28073248 (PMC10096123; doi:10.3390/molecules28073248)
Supplement: Supplementary file 1 [file molecules-28-03248-s001.zip › molecules-2305256-supplementary.pdf]

Supplementary Material

14-Substituted Diquinothiazines as a New Group of Anticancer Agents

Małgorzata Jeleń [1,*], Krystian Pluta [1], Małgorzata Szmielew [1], Beata Morak-Młodawska [1], Kinga Herman [2], Klaudia Giercuszkiewicz [3], Anna Kasprzycka [2,4] and Magdalena Skonieczna [3,4,*]

[1] Department of Organic Chemistry, Faculty of Pharmaceutical Sciences in Sosnowiec, The Medical University of Silesia, Jagiellońska 4, 41-200 Sosnowiec, Poland; bmlodawska@sum.edu.pl (B.M.-M.)
[2] Department of Organic Chemistry, Bioorganic Chemistry and Biotechnology, Faculty of Chemistry, Silesian University of Technology, Krzywoustego Street 4, 44-100 Gliwice, Poland; kingher651@student.polsl.pl (K.H.); anna.kasprzycka@polsl.pl (A.K.)
[3] Department of Systems Biology and Engineering, The Silesian University of Technology, Akademicka Street 16, 44–100 Gliwice, Poland; klaudia.giercuszkiewicz@polsl.pl
[4] Centre of Biotechnology, Silesian University of Technology, Krzywoustego Street 8, 44-100 Gliwice, Poland
* Correspondence: manowak@sum.edu.pl (M.J.); magdalena.skonieczna@polsl.pl (M.S.); Tel.: +48-32-364-16-04 (M.J.); +48-32-237-11-68 (M.S.)

Content **1**

1. **Figure S1.** Survival fraction of HCT116 (left) and SH-5YSY (right) cell lines after 72 h of incubation with tested compounds and positive controls, anticancer drug - etoposide evaluated by MTT assay. Results presented as mean form 3 experiments, +/- SD. Statistical significance indicated by star; evaluated by T-test, where $p<0.05$ (TRUE bolded under the charts). **2**
2. **Figure S2.** Typical histograms of cell cycle distribution in control and treated HCT116 cells after 72 h of incubation with tested compounds, at dose of 100 μM. DNA gating during cytometry analyses after iodium propide staining (PI; 100 μg/ml; [a.u.]) showed the cells in cell cycle phases: sub G1; G0/G1; S and G2/M, respectively. **9**
3. Spectra of compounds **2-6**. **10**

Figure S1. Survival fraction of HCT116 (left) and SH-5YSY (right) cell lines after 72 h of incubation with tested compounds and positive controls, anticancer drug-etoposide, respectively, evaluated by MTT assay. Results presented as mean form 3 experiments, +/- SD. Statistical significance indicated by star; evaluated by T-test, where p<0.05 (TRUE bolded under the charts).

M0

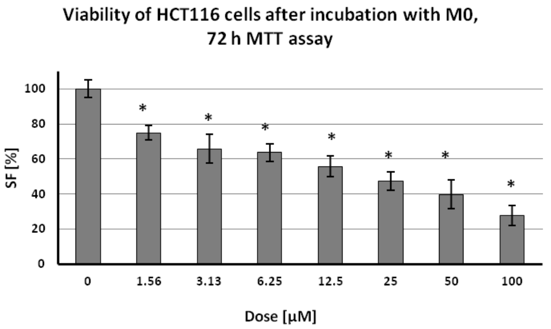

P value	0.0005	0.0186	0.0061	0.0062	0.0032	0.0059	0.0021
P<0.05	TRUE	TRUE	TRUE	TRUE	TRUE	TRUE	TRUE

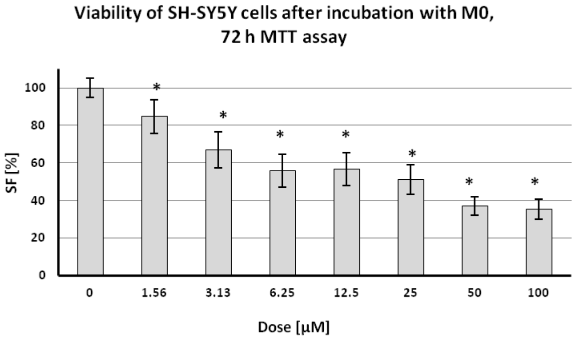

P value	0.0026	0.0274	0.0126	0.0133	0.0086	0.0020	0.0023
P<0.05	TRUE	TRUE	TRUE	TRUE	TRUE	TRUE	TRUE

2a

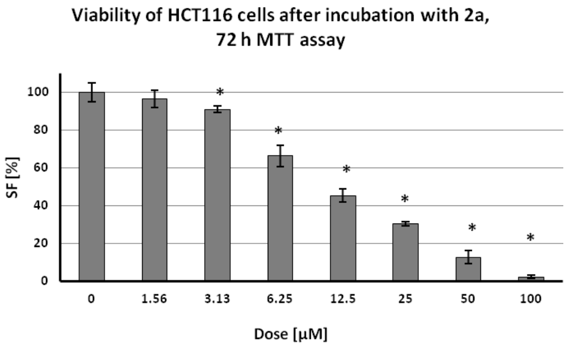

P value	0.0505	0.0142	0.0093	0.0014	0.0001	0.0006	3.04^{E-05}
P<0.05	FALSE	TRUE	TRUE	TRUE	TRUE	TRUE	TRUE

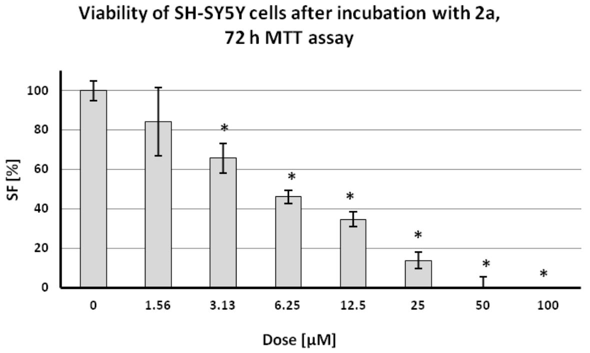

P value	0.0529	0.0154	0.0012	0.0011	0.0008	0.0011	8.08^{E-05}
P<0.05	FALSE	TRUE	TRUE	TRUE	TRUE	TRUE	TRUE

2b

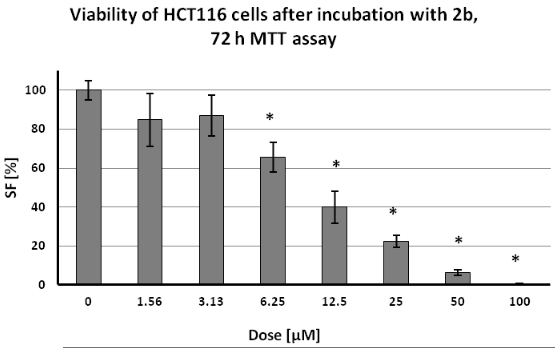

P value	0,0884	0,1673	0,0152	0,0061	0,0004	1.53^{E-05}	2.06^{E-09}
P<0.05	FALSE	FALSE	TRUE	TRUE	TRUE	TRUE	TRUE

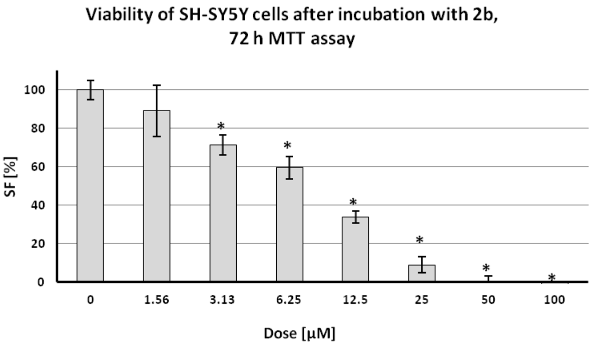

P value	0.0541	0.0011	0.0069	0.0008	0.0007	0.0004	0.0002
P<0.05	FALSE	TRUE	TRUE	TRUE	TRUE	TRUE	TRUE

2c

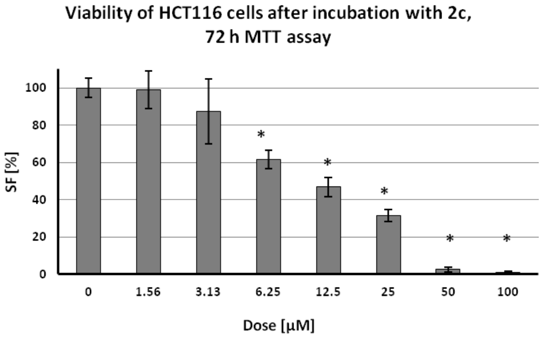

P value	0,3356	0.2315	**0.0007**	**0.0029**	**0.0051**	**4.65^{E-06}**	**0.0032**
P<0.05	FALSE	FALSE	TRUE	TRUE	TRUE	TRUE	TRUE

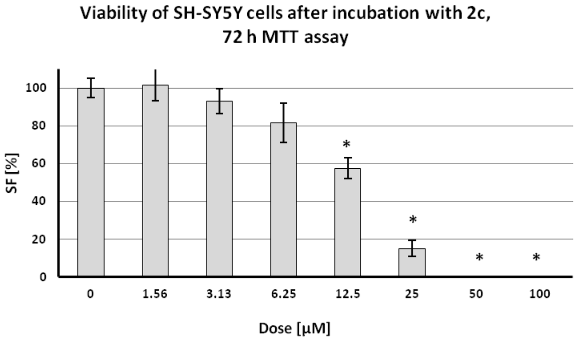

P value	0.0511	0.2196	0.0929	**0.0055**	**0.0008**	**0.0001**	**4.72^{E-05}**
P<0.05	FALSE	FALSE	FALSE	TRUE	TRUE	TRUE	TRUE

2d

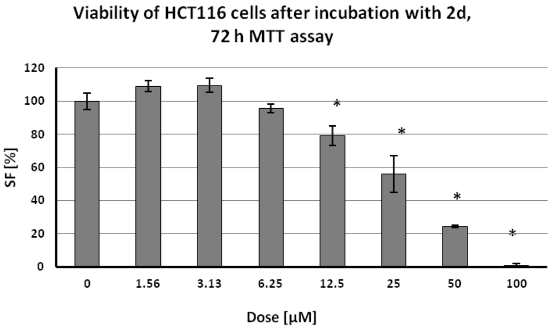

P value	0,1146	0,5698	0,7935	**0.0261**	**0.0205**	**6.51^{E-06}**	**1.55^{E-05}**
P<0.05	FALSE	FALSE	FALSE	TRUE	TRUE	TRUE	TRUE

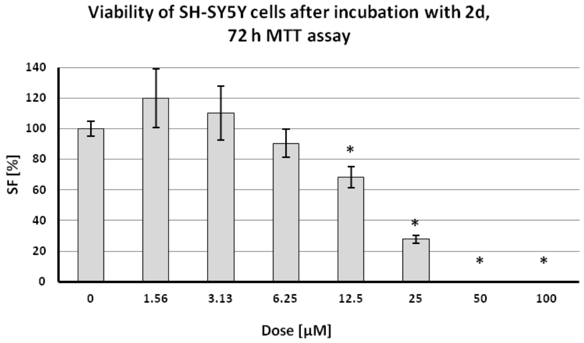

P value	0.0071	0.4226	0.2096	**0.0149**	**0.0005**	**0.0002**	**0.0002**
P<0.05	FALSE	FALSE	FALSE	TRUE	TRUE	TRUE	TRUE

2e

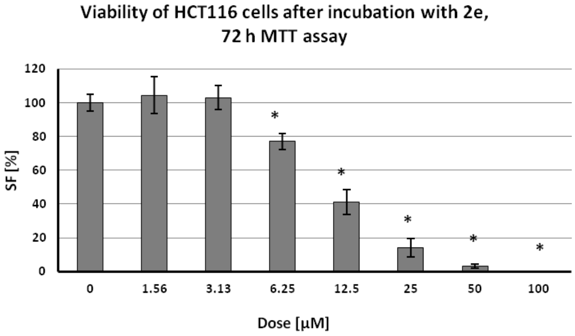

P value	0.,0507	0.5505	**0.0013**	**0.0051**	**0.0139**	**0.0248**	**0.0035**
P<0.05	FALSE	FALSE	TRUE	TRUE	TRUE	TRUE	TRUE

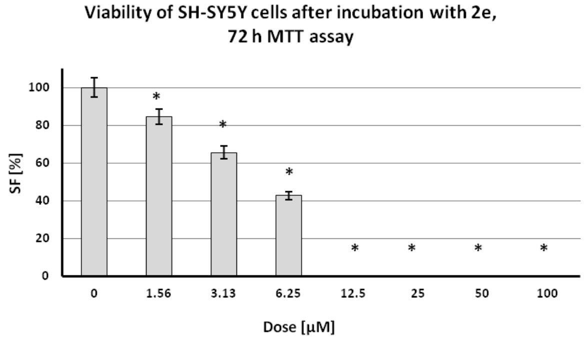

P value	**4.73^{E-09}**	**2.44^{E-08}**	**1.75^{E-07}**	**3.53^{E-08}**	**6.31^{E-08}**	**6.77^{E-08}**	**6.55^{E-08}**
P<0.05	TRUE	TRUE	TRUE	TRUE	TRUE	TRUE	TRUE

2f

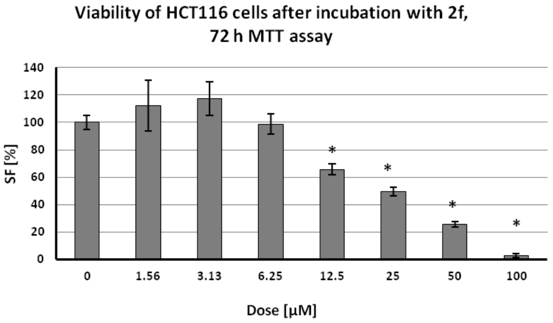

P value	0.0934	0.1359	0.8056	0.0482	0.0013	0.0002	7.77^{E-05}
P<0.05	FALSE	FALSE	FALSE	TRUE	TRUE	TRUE	TRUE

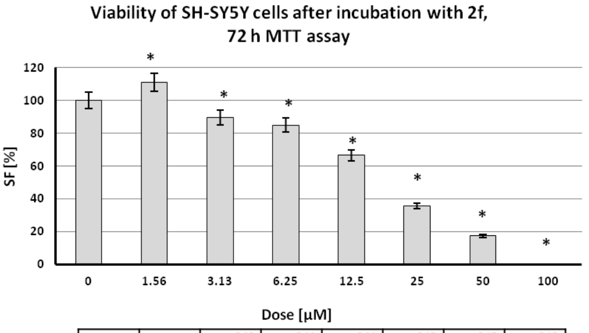

P value	5.3^{E-08}	1.36^{E-05}	8.63^{E-06}	1.65^{E-06}	6.44^{E-07}	3.61^{E-07}	2.87^{E-07}
P<0.05	TRUE	TRUE	TRUE	TRUE	TRUE	TRUE	TRUE

2g

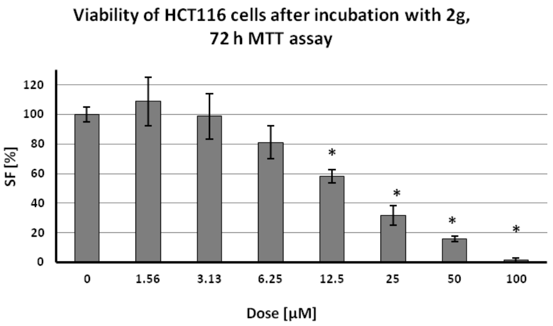

P value	0,0751	0,8962	0,0993	0,0037	0,0029	0,0001	1.33^{E-05}
P<0.05	FALSE	FALSE	FALSE	TRUE	TRUE	TRUE	TRUE

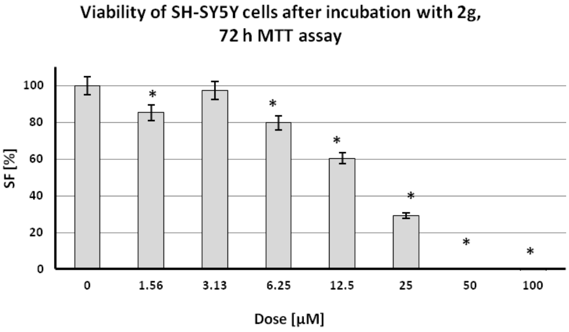

P value	4.5^{E-09}	0.0911	1.95^{E-07}	2.02^{E-08}	2.72^{E-09}	7.09^{E-10}	5.27^{E-10}
P<0.05	TRUE	FALSE	TRUE	TRUE	TRUE	TRUE	TRUE

3a

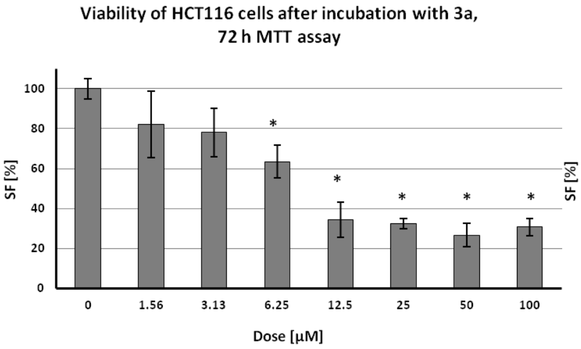

P value	0,0559	0,0885	0,0162	0,0059	0,0003	0,0021	0,0137
P<0.05	FALSE	FALSE	TRUE	TRUE	TRUE	TRUE	TRUE

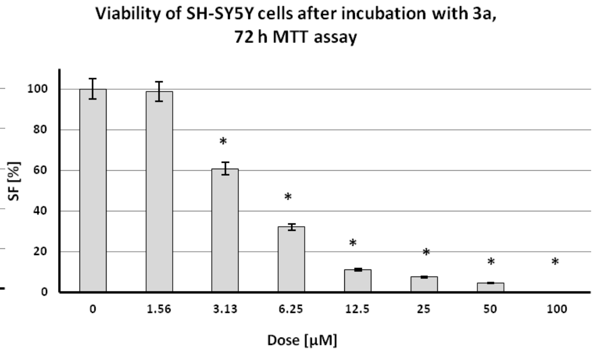

P value	0.0607	9.78^{E-08}	3.26^{E-08}	1.42^{E-09}	2.18^{E-10}	5.61^{E-10}	5.09^{E-10}
P<0.05	FALSE	TRUE	TRUE	TRUE	TRUE	TRUE	TRUE

3b

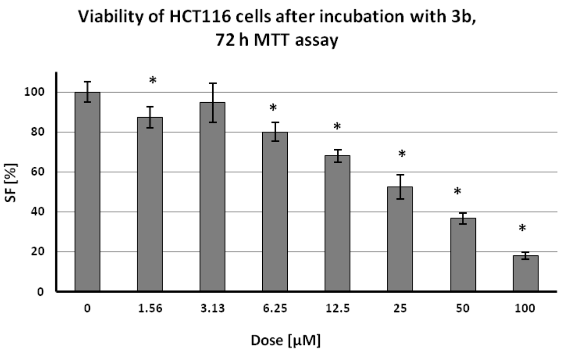

P value	0.0007	0.4456	0.0187	0.0032	0.0056	0.0006	0.0002
P<0.05	TRUE	FALSE	TRUE	TRUE	TRUE	TRUE	TRUE

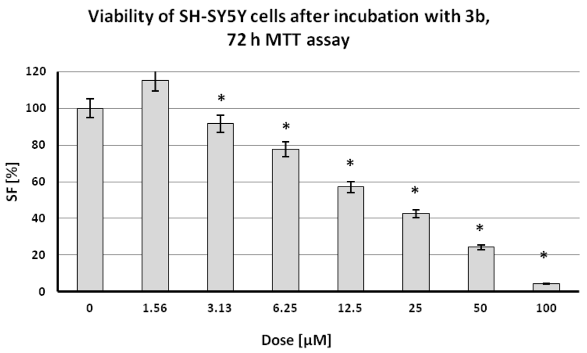

P value	0.0638	2.6^{E-06}	6.7^{E-07}	1.02^{E-07}	1.16^{E-08}	3.41^{E-09}	1.83^{E-10}
P<0.05	FALSE	TRUE	TRUE	TRUE	TRUE	TRUE	TRUE

3c

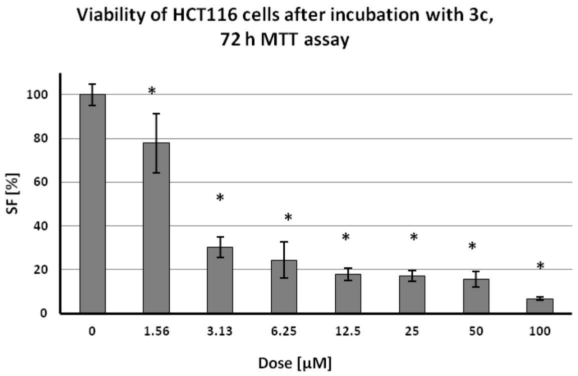

P value	0.0115	0.0015	0.0041	0.0004	0.0003	0.0006	2.6^{E-05}
P<0.05	TRUE	TRUE	TRUE	TRUE	TRUE	TRUE	TRUE

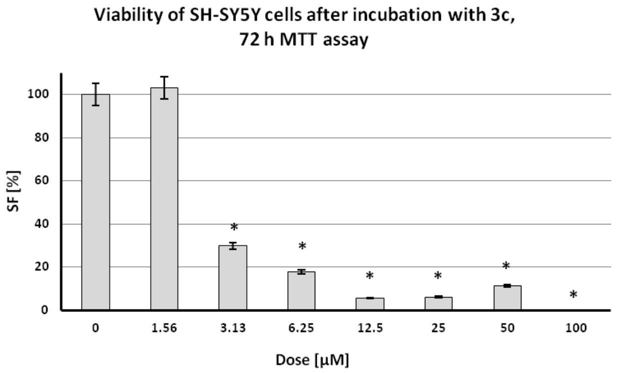

P value	0.0417	3.67^{E-09}	9.15^{E-09}	6.36^{E-10}	1.41^{E-09}	3.35^{E-09}	5.45^{E-10}
P<0.05	FALSE	TRUE	TRUE	TRUE	TRUE	TRUE	TRUE

5a

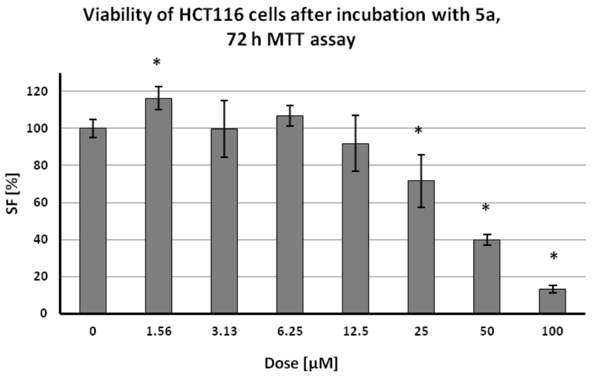

P value	0,0005	0,9881	0,1627	0,4481	0,0444	0,0007	0,0002
P<0.05	TRUE	FALSE	FALSE	FALSE	TRUE	TRUE	TRUE

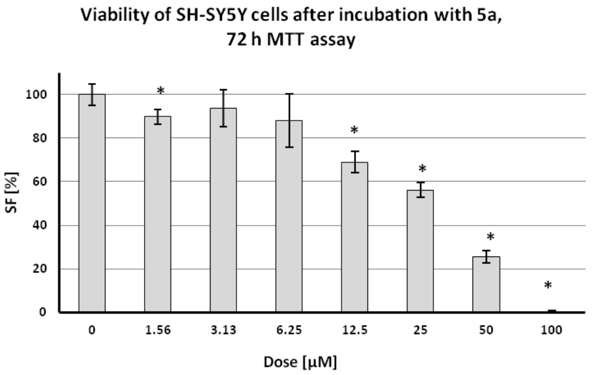

P value	4.58^{E-06}	0.3311	0.2369	0.0081	0.0019	0.0005	0.0003
P<0.05	TRUE	FALSE	FALSE	TRUE	TRUE	TRUE	TRUE

5b

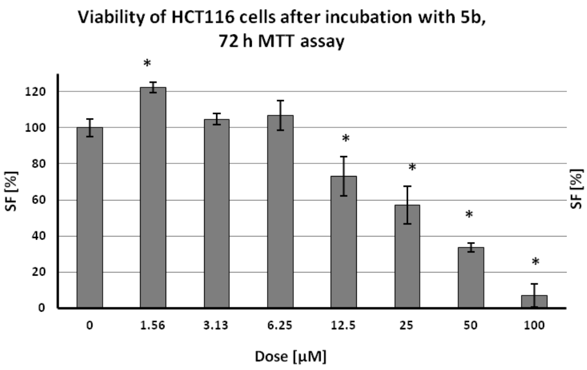

P value	0.0179	0.8115	0.2937	0.0143	0.0194	0.0005	0.0016
P<0.05	TRUE	FALSE	FALSE	TRUE	TRUE	TRUE	TRUE

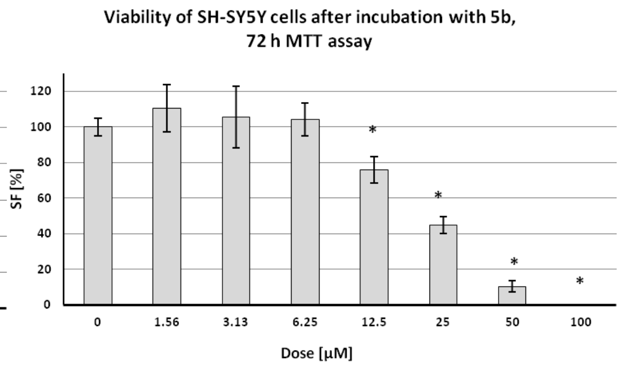

P value	0.0538	0.6326	0.4984	0.0291	0.0025	0.0004	4.3^{E-05}
P<0.05	FALSE	FALSE	FALSE	TRUE	TRUE	TRUE	TRUE

5c

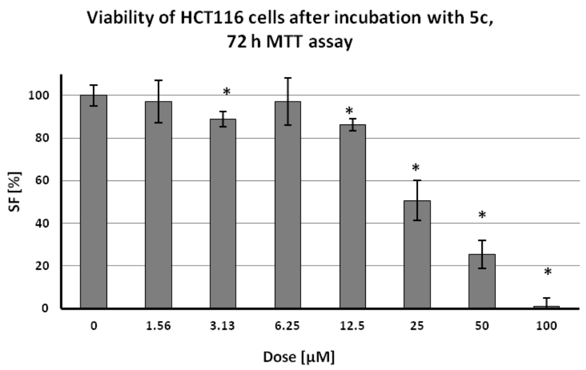

P value	0,1011	0,0327	0,6919	0,0125	0,0117	0,0023	0,0006
P<0.05	FALSE	TRUE	FALSE	TRUE	TRUE	TRUE	TRUE

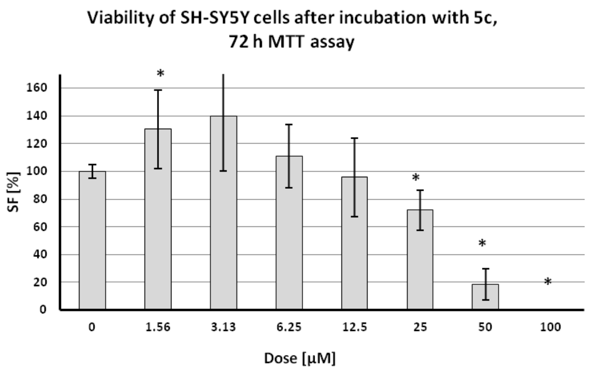

P value	0.0133	0.2226	0.4891	0.81854	0.0774	0.0065	5.49^{E-05}
P<0.05	TRUE	FALSE	FALSE	FALSE	TRUE	TRUE	TRUE

6a

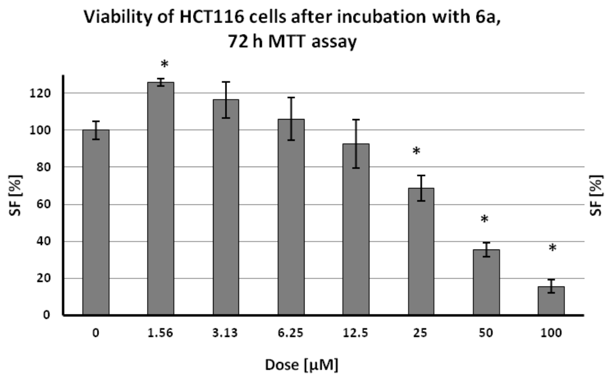

P value	0.0109	0.0999	0.4652	0.4274	0.0156	0.0011	9.2^{E-06}
P<0.05	TRUE	FALSE	FALSE	FALSE	TRUE	TRUE	TRUE

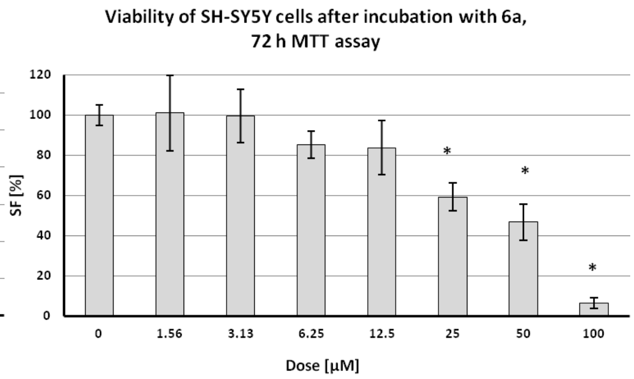

P value	0.0512	0.9512	0.0632	0.1689	0.0094	0.0092	0.0003
P<0.05	FALSE	FALSE	FALSE	FALSE	TRUE	TRUE	TRUE

6b

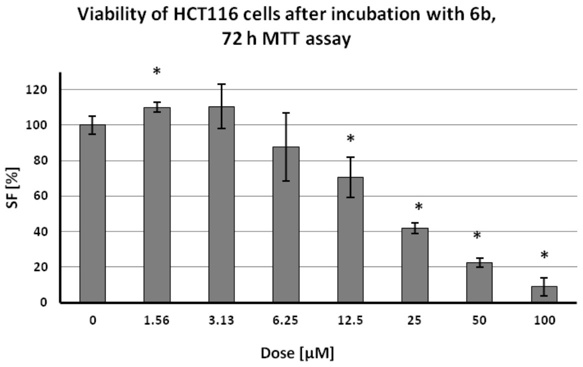

P value	0.019	0.6445	0.3781	0.0457	0.0009	0.0003	0.0011
P<0.05	TRUE	FALSE	FALSE	TRUE	TRUE	TRUE	TRUE

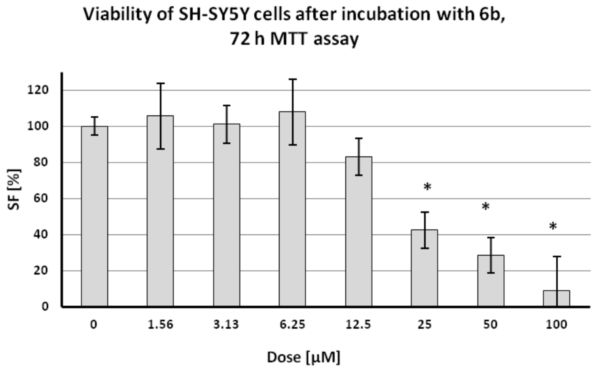

P value	0.0531	0.8693	0.5284	0.1019	0.0099	0.0061	0.0136
P<0.05	FALSE	FALSE	FALSE	FALSE	TRUE	TRUE	TRUE

6c

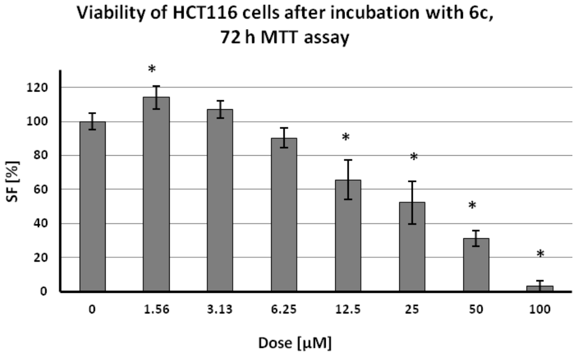

P value	0.0002	0.1305	0.1067	0.0363	0.0228	0.0015	0.0003
P<0.05	TRUE	FALSE	FALSE	TRUE	TRUE	TRUE	TRUE

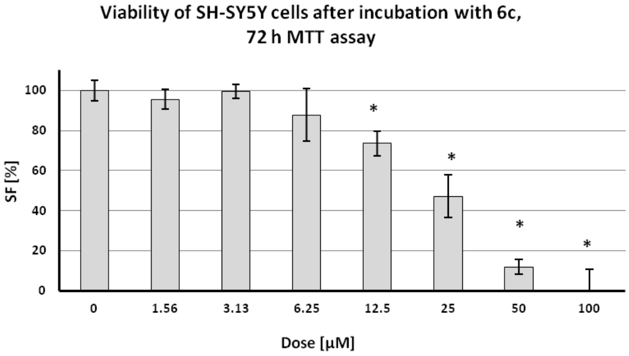

P value	0.0016	0.8235	0.2452	0.0178	0.0133	0.0006	0.0044
P<0.05	FALSE	FALSE	FALSE	TRUE	TRUE	TRUE	TRUE

Etoposide

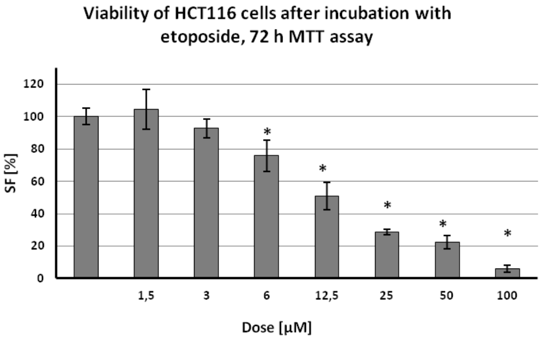

P value	0.1556	0.8089	0.0107	0.0316	0.0272	0.0471	0.0478
P<0.05	FALSE	FALSE	TRUE	TRUE	TRUE	TRUE	TRUE

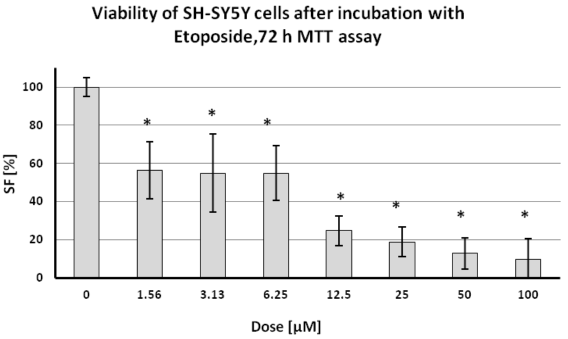

P value	0.0149	0.0427	0.0321	0.0047	0.0031	0.0031	0.0049
P<0.05	TRUE	TRUE	TRUE	TRUE	TRUE	TRUE	TRUE

Figure S2. Typical histograms of cell cycle distribution in control and treated HCT116 cells after 72 h of incubation with tested compounds, at dose of 100 µM. DNA gating during cytometry analyses after iodium propide staining (PI; 100 µg/ml; [a.u.]) showed the cells in cell cycle phases: subG1; G0/G1; S and G2/M, respectively.

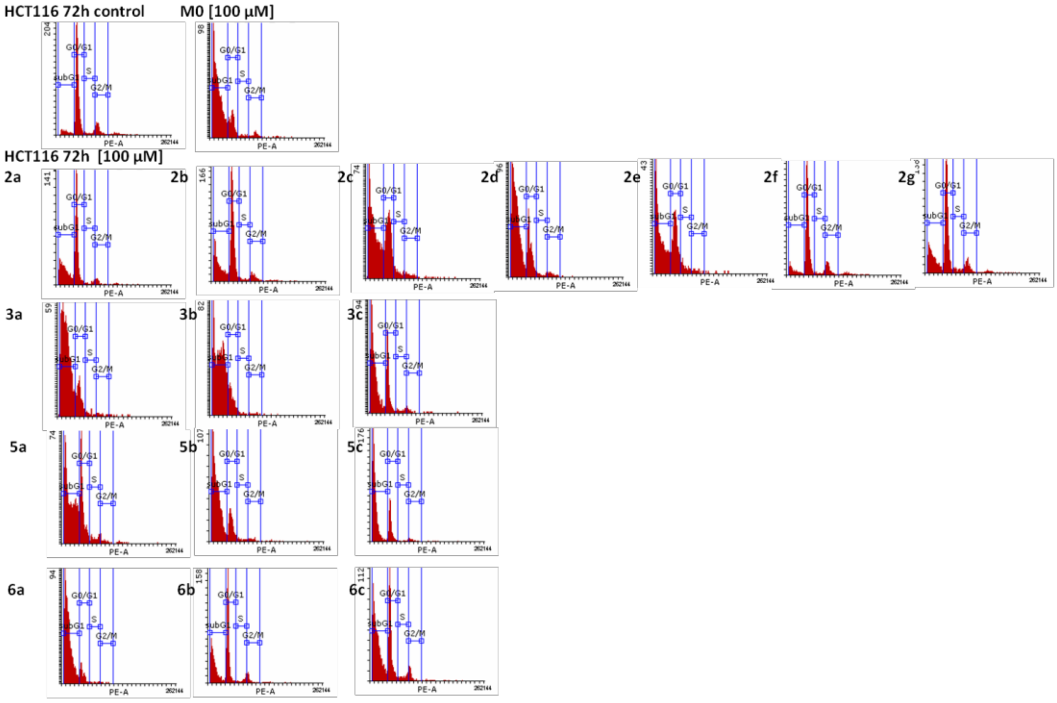

Figure S3. Typical histograms of cell cycle distribution in control and treated SH-5YSY cells after 72 h of incubation with tested compounds, at dose of 100 µM. DNA gating during cytometry analyses after iodium propide staining (PI; 100 µg/ml; [a.u.]) showed the cells in cell cycle phases: subG1; G0/G1; S and G2/M, respectively.

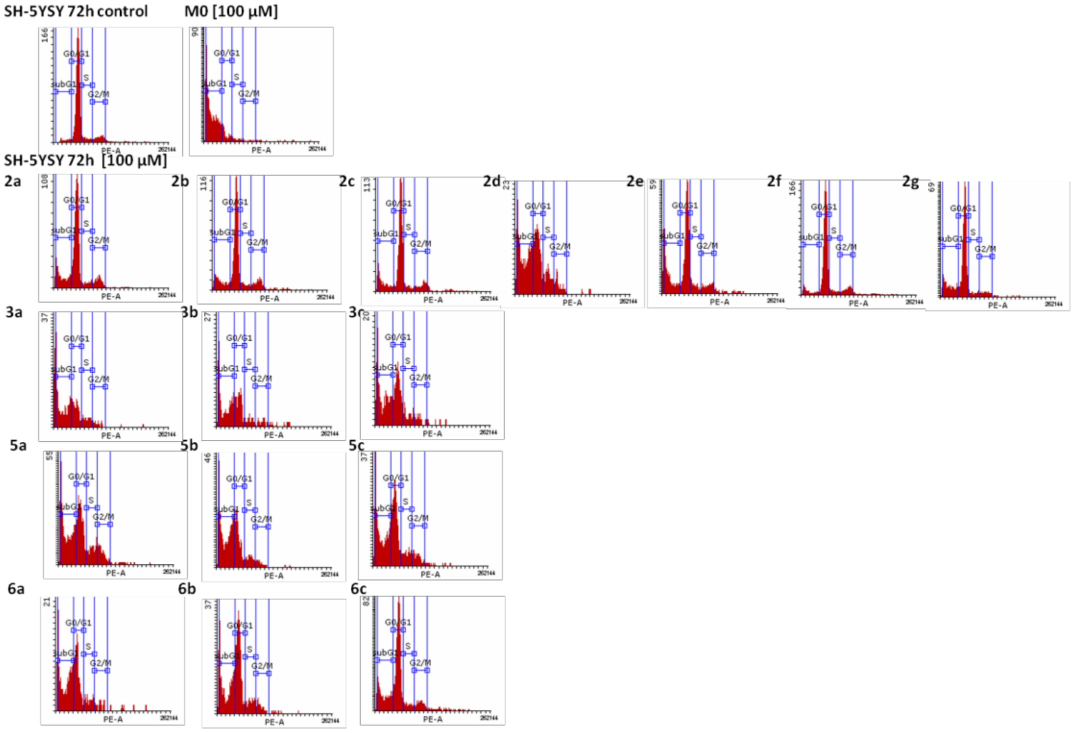

Spectra of compounds **2-6**.

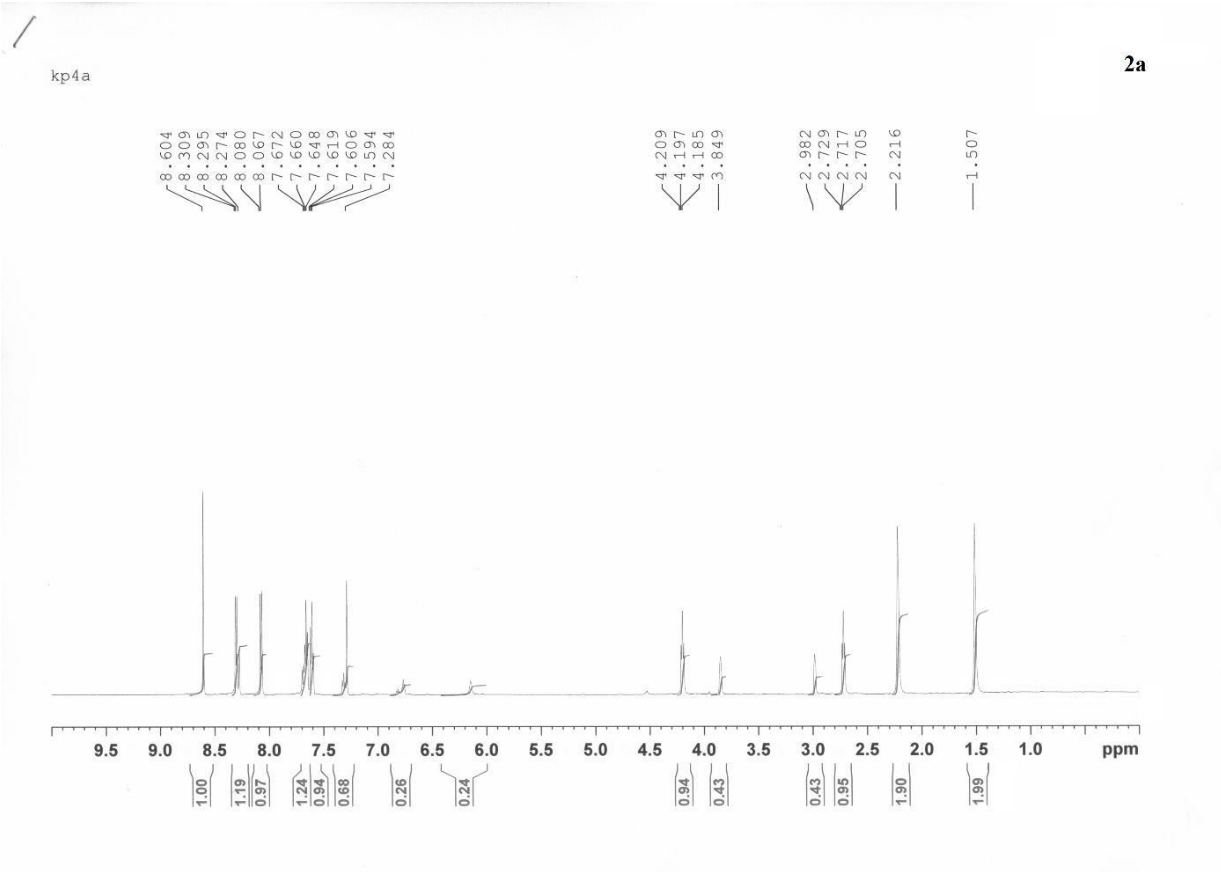

^{1}H NMR of 14-(pyrrolidinylethyl)diquinothiazine (**2a**)

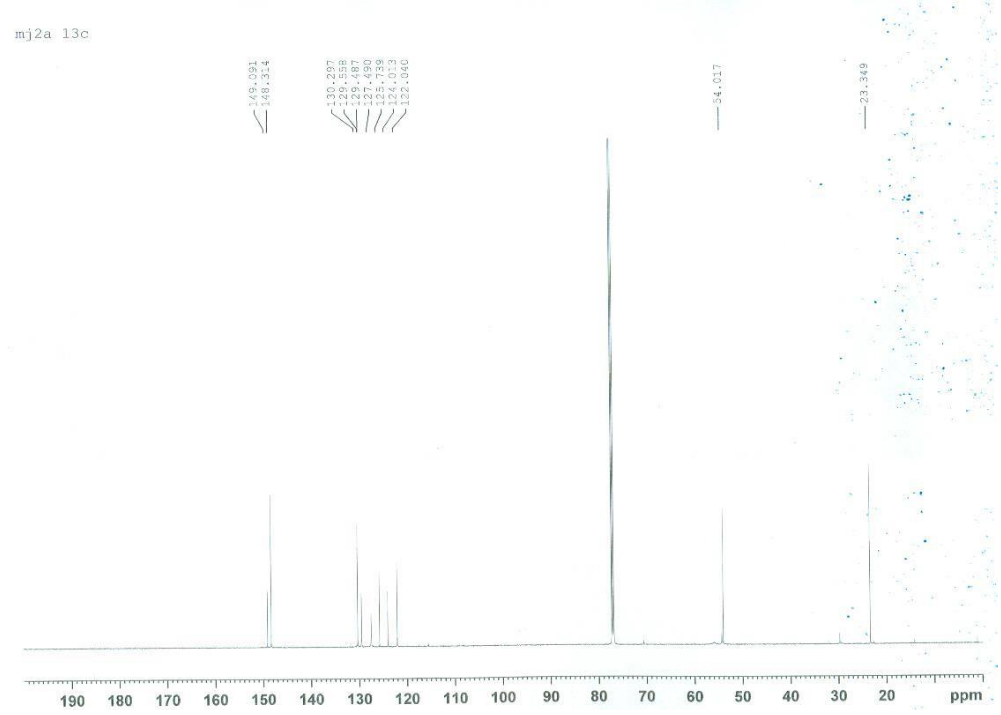

^{13}C NMR of 14-(pyrrolidinylethyl)diquinothiazine (**2a**)

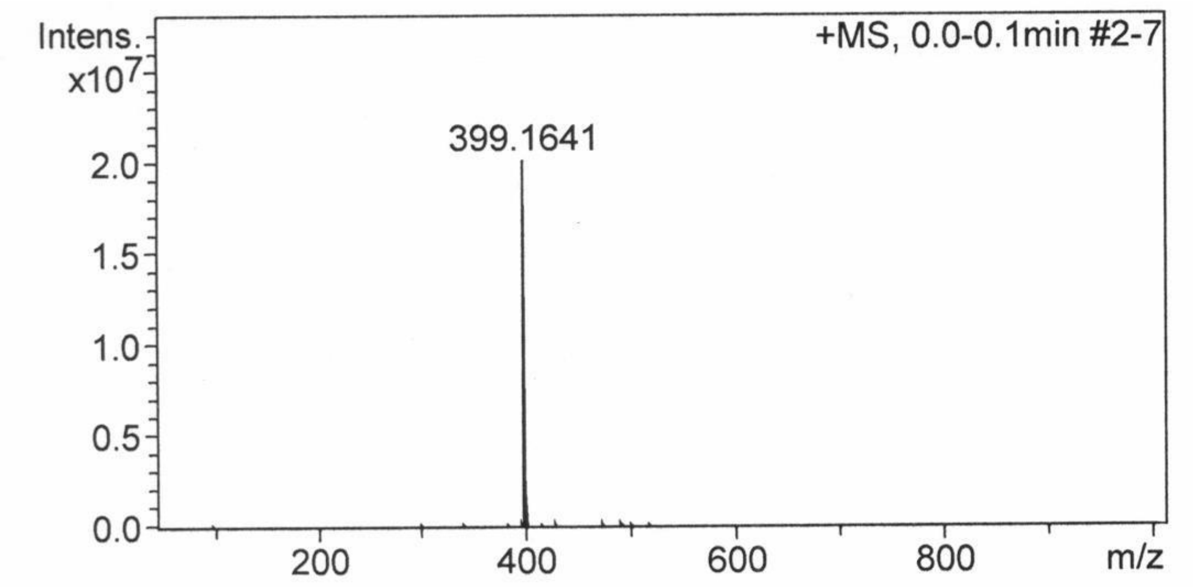

#	m/z	Res.	S/N	I	I %	FWHM
1	399.1641	14893	230314.8	20151970	100.0	0.0268

HR MS of 14-(pyrrolidinylethyl)diquinothiazine (**2a**) (calcd m/z = 399.1643)

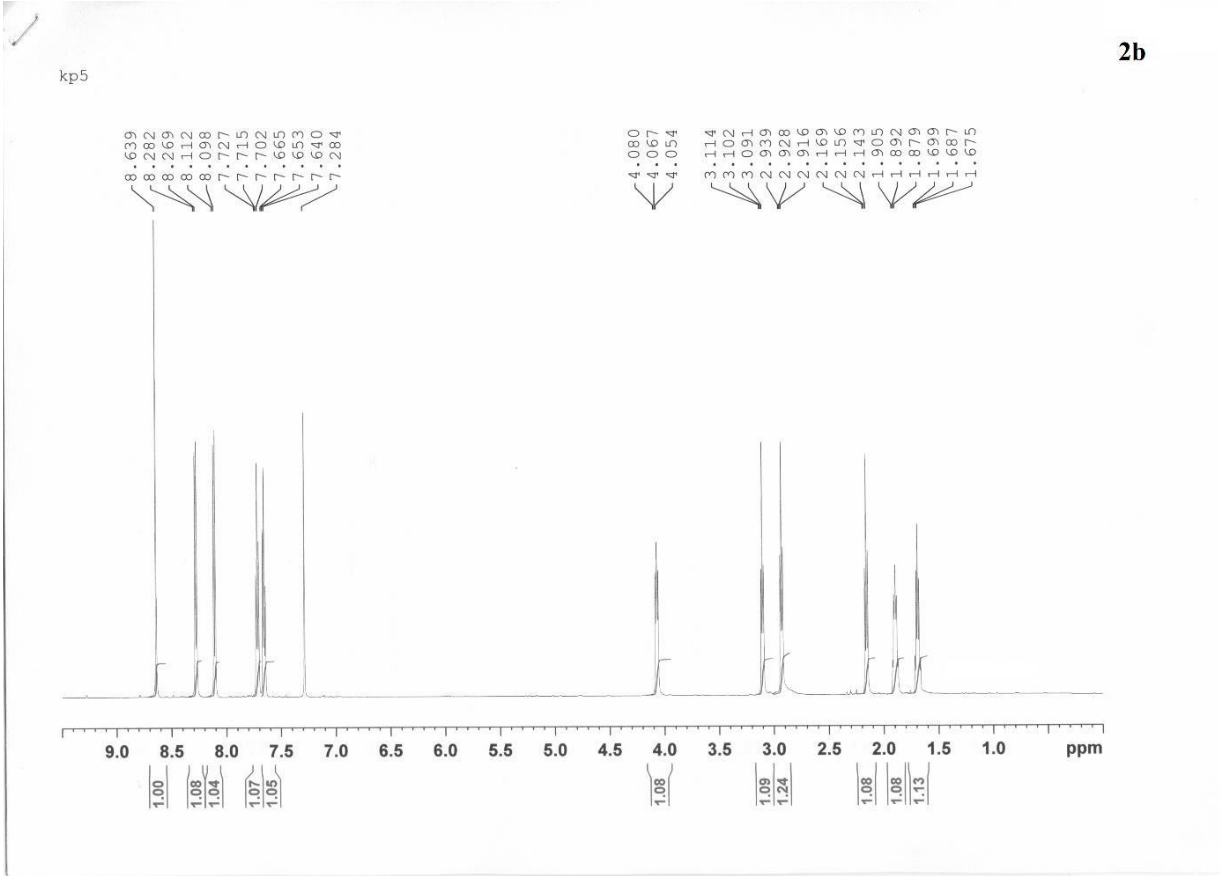

^{1}H NMR of 14-(pyrrolidinonylpropyl)diquinothiazine (**2b**)

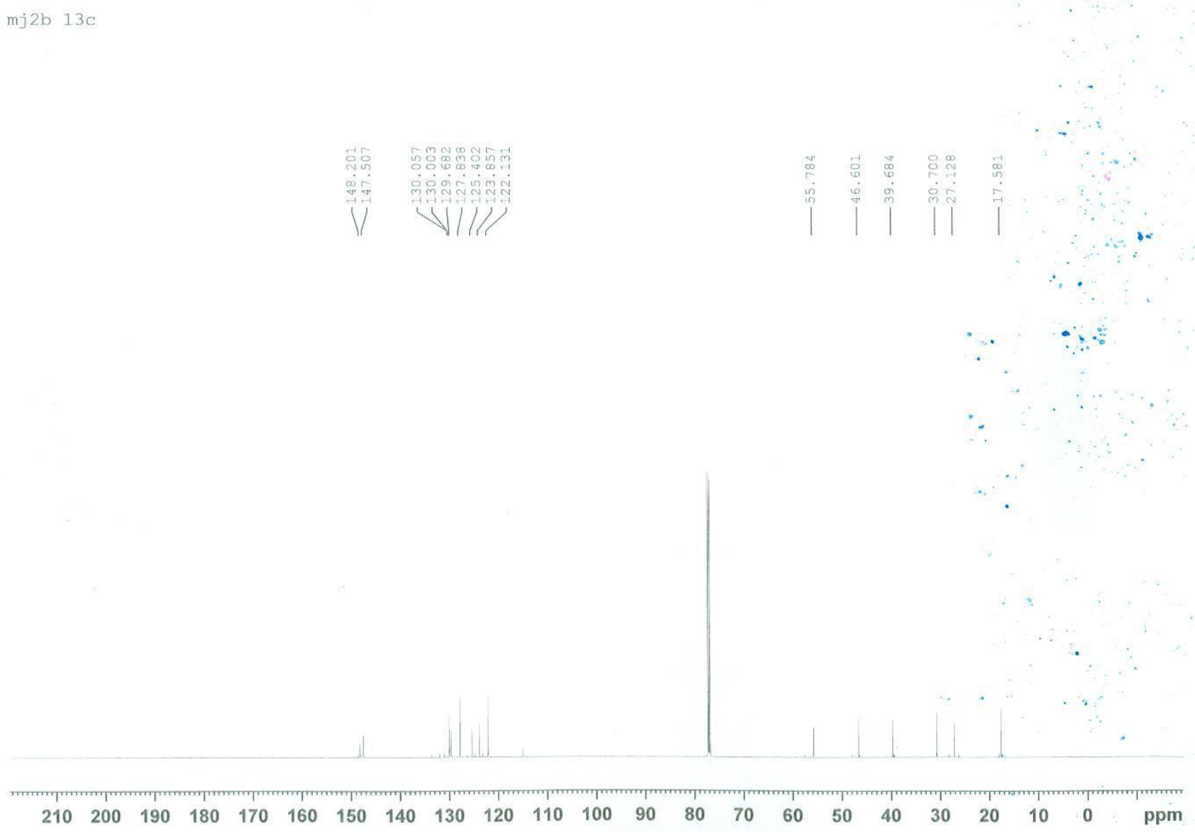

^{13}C NMR of 14-(pyrrolidinonylpropyl)diquinothiazine (**2b**)

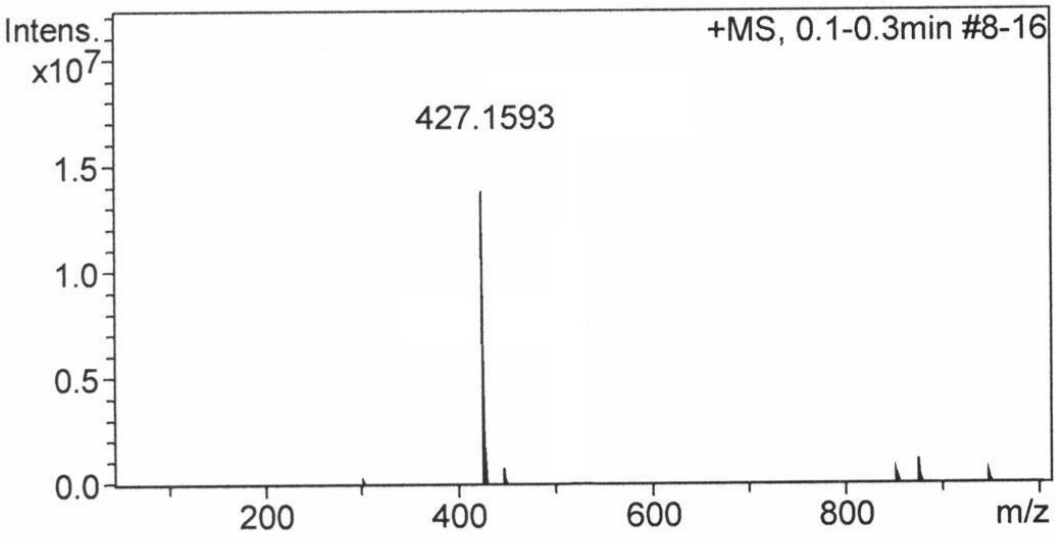

#	m/z	Res.	S/N	I	I %	FWHM
1	427.1593	39005	87911.5	6631967	41.5	0.0110

HR MS of 14-(pyrrolidinonylpropyl)diquinothiazine (**2b**) (calcd m/z = 427.1593)

2c

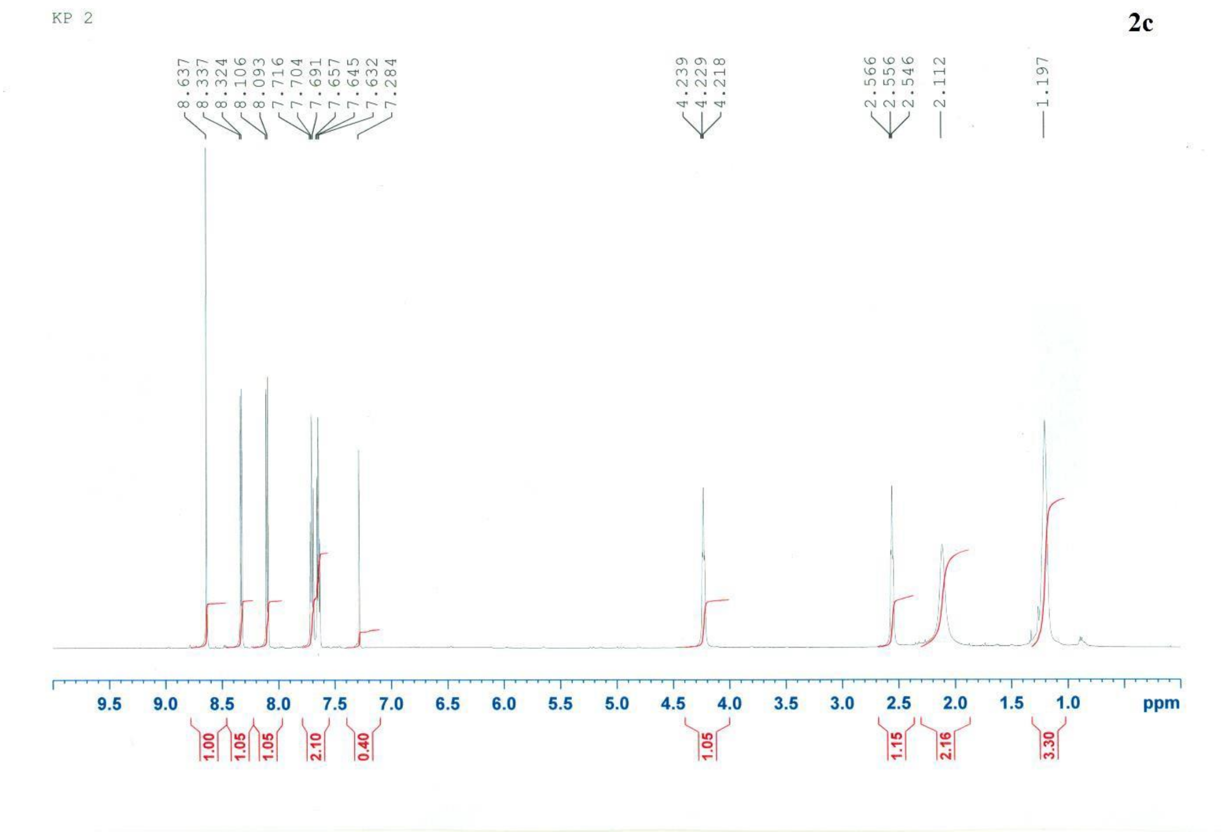

^{1}H NMR of 14-(piperidinylethyl)diquinothiazine (**2c**)

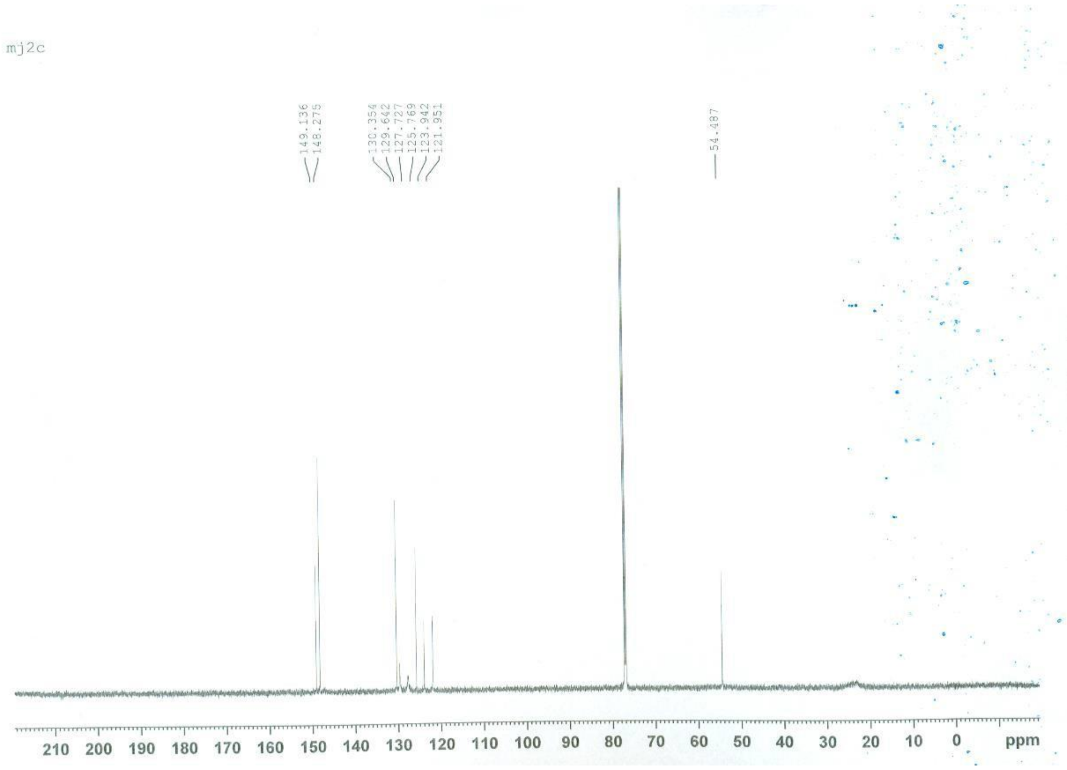

^{13}C NMR of 14-(piperidinylethyl)diquinothiazine (**2c**)

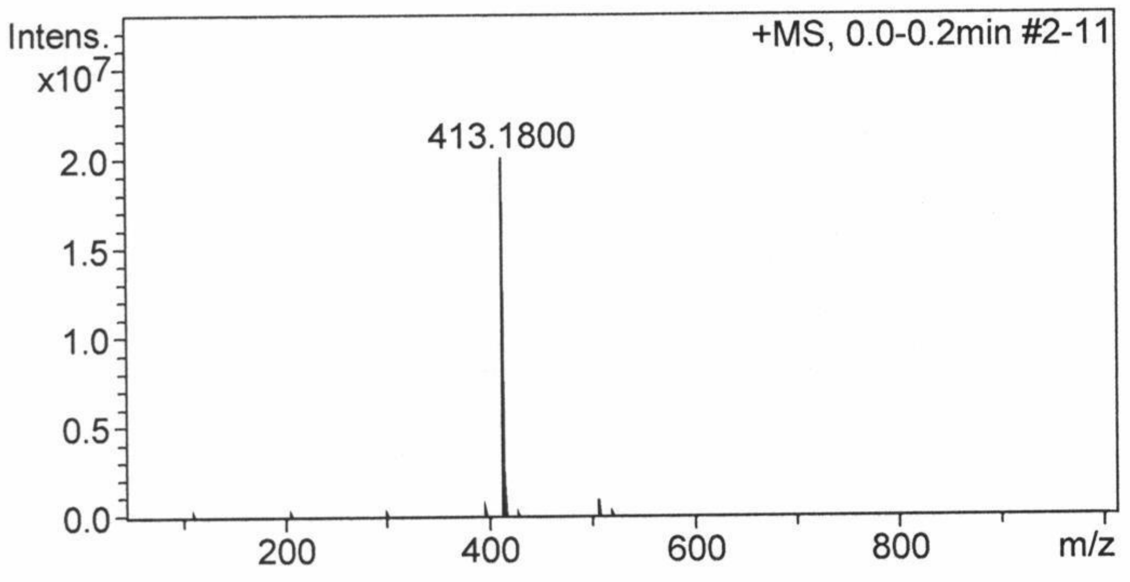

#	m/z	Res.	S/N	I	I %	FWHM
1	413.1800	15330	334518.1	20084228	100.0	0.0270

HR MS of 14-(piperidinylethyl)diquinothiazine (**2c**) (calcd m/z = 413.1800)

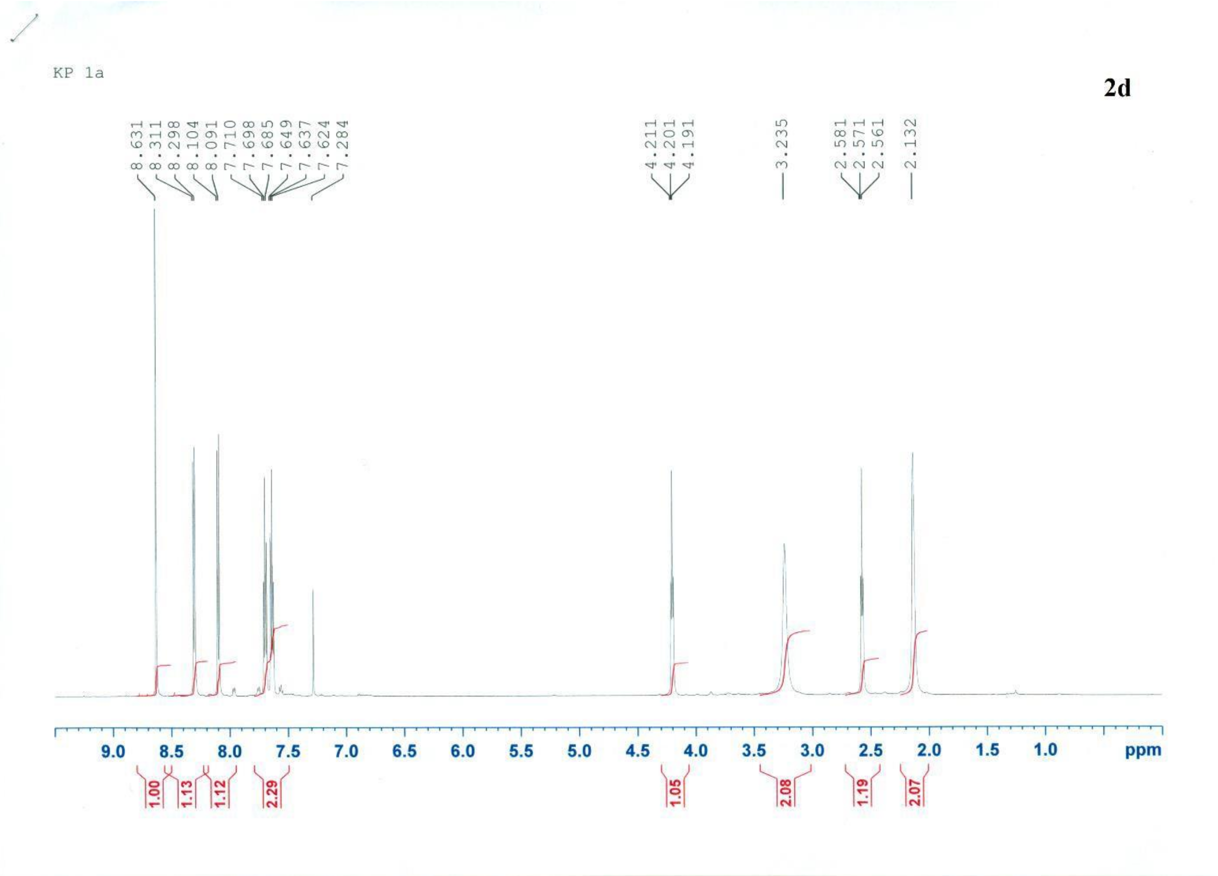

^{1}H NMR of 14-(morpholinylethyl)diquinothiazine (**2d**)

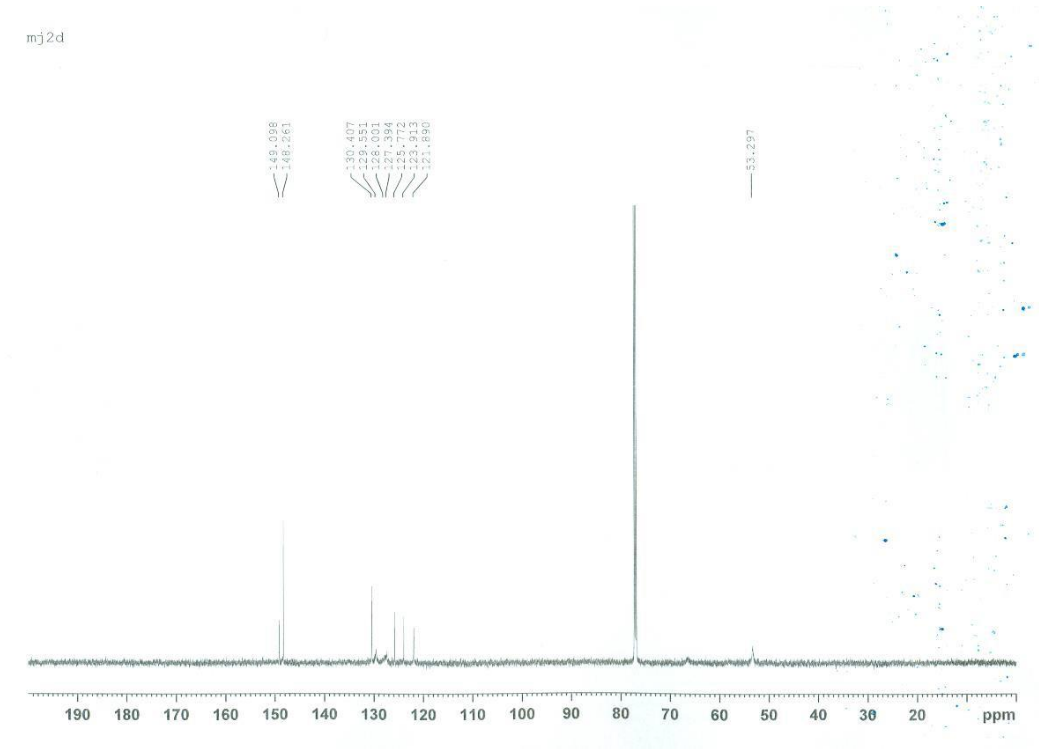

^{13}C NMR of 14-(morpholinylethyl)diquinothiazine (**2d**)

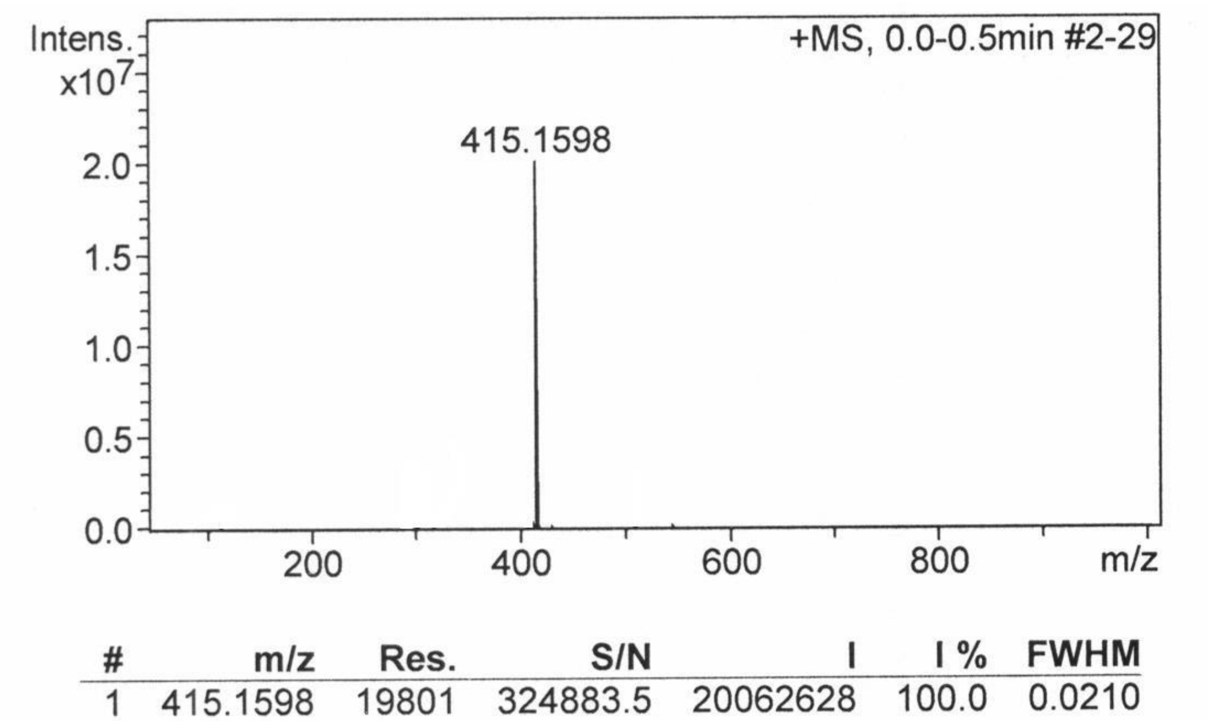

#	m/z	Res.	S/N	I	I %	FWHM
1	415.1598	19801	324883.5	20062628	100.0	0.0210

HR MS of 14-(morpholinylethyl)diquinothiazine (**2d**) (calcd m/z = 415.1593)

2e

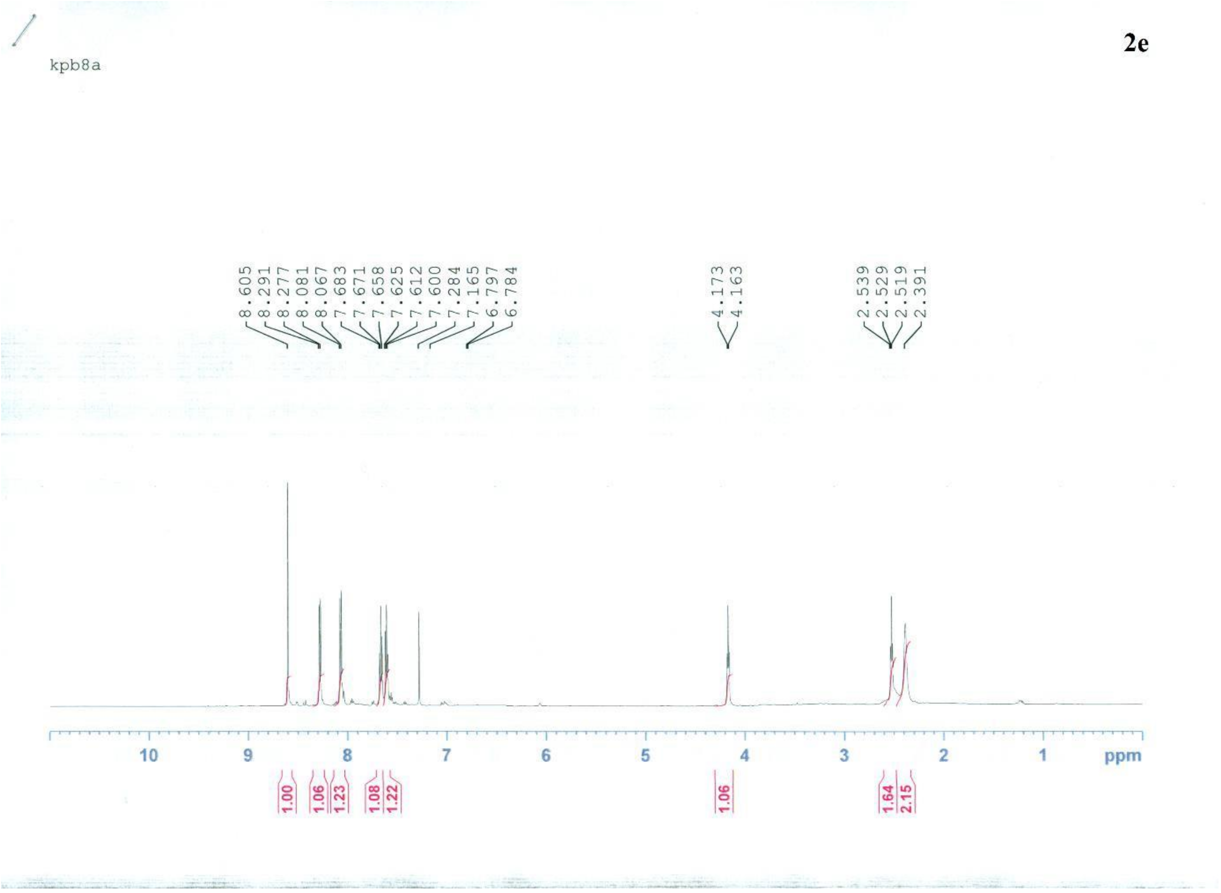

^{1}H NMR of 14-(piperazinyloethyl)dichinotiazyny (**2e**)

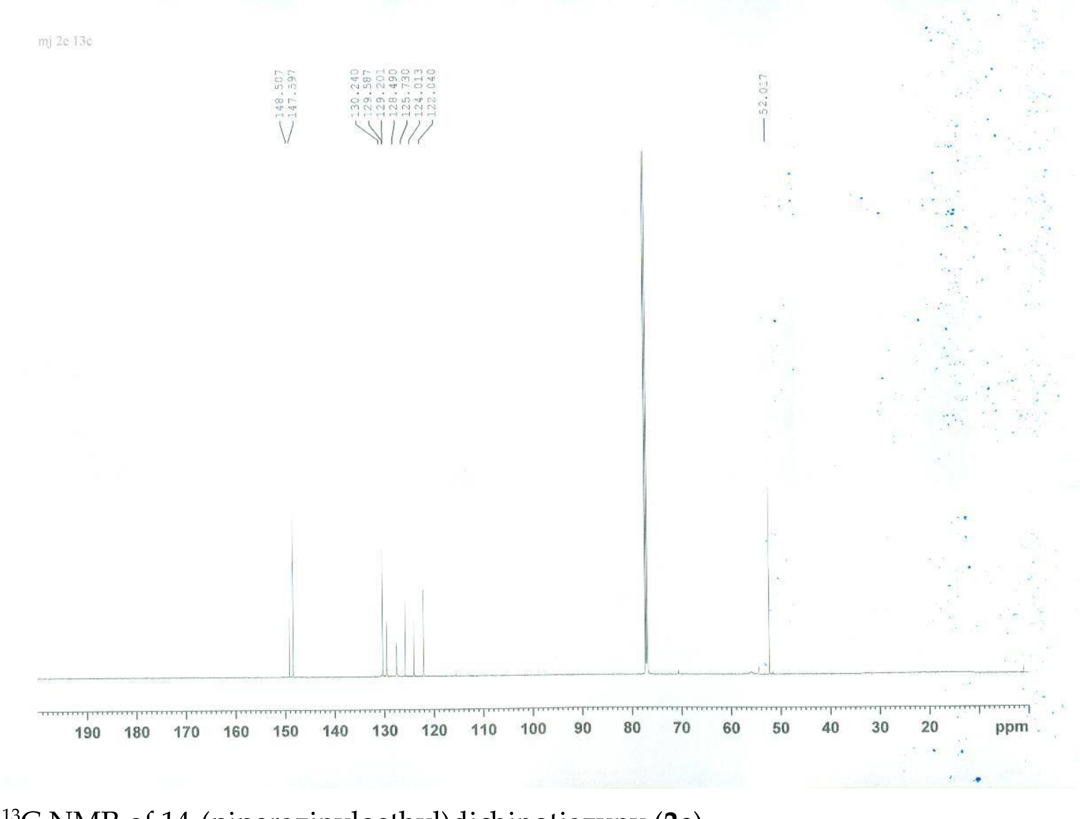

^{13}C NMR of 14-(piperazinyloethyl)dichinotiazyny (**2e**)

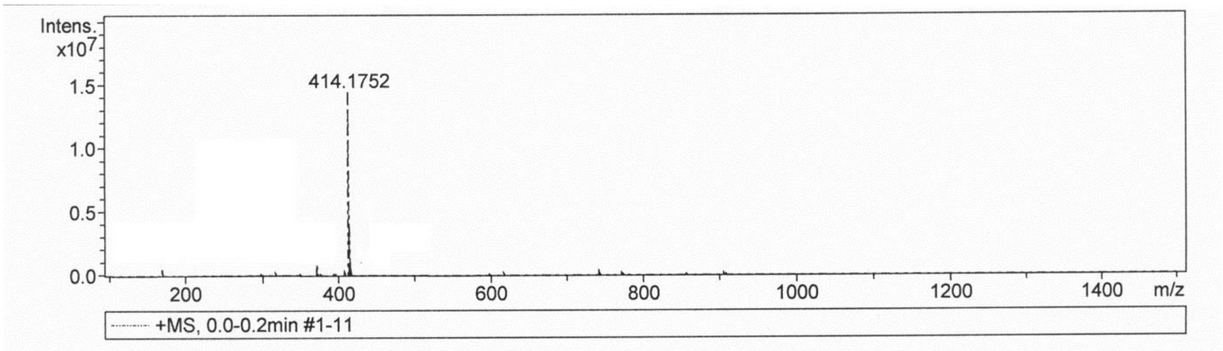

#	m/z	Res.	S/N	I	I %	FWHM
1	414.1752	44214	28855.6	14404244	100.0	0.0094

HR MS of 14-(piperazinyloethyl)dichinotiazyny (**2e**) (calcd m/z = 414.1752)

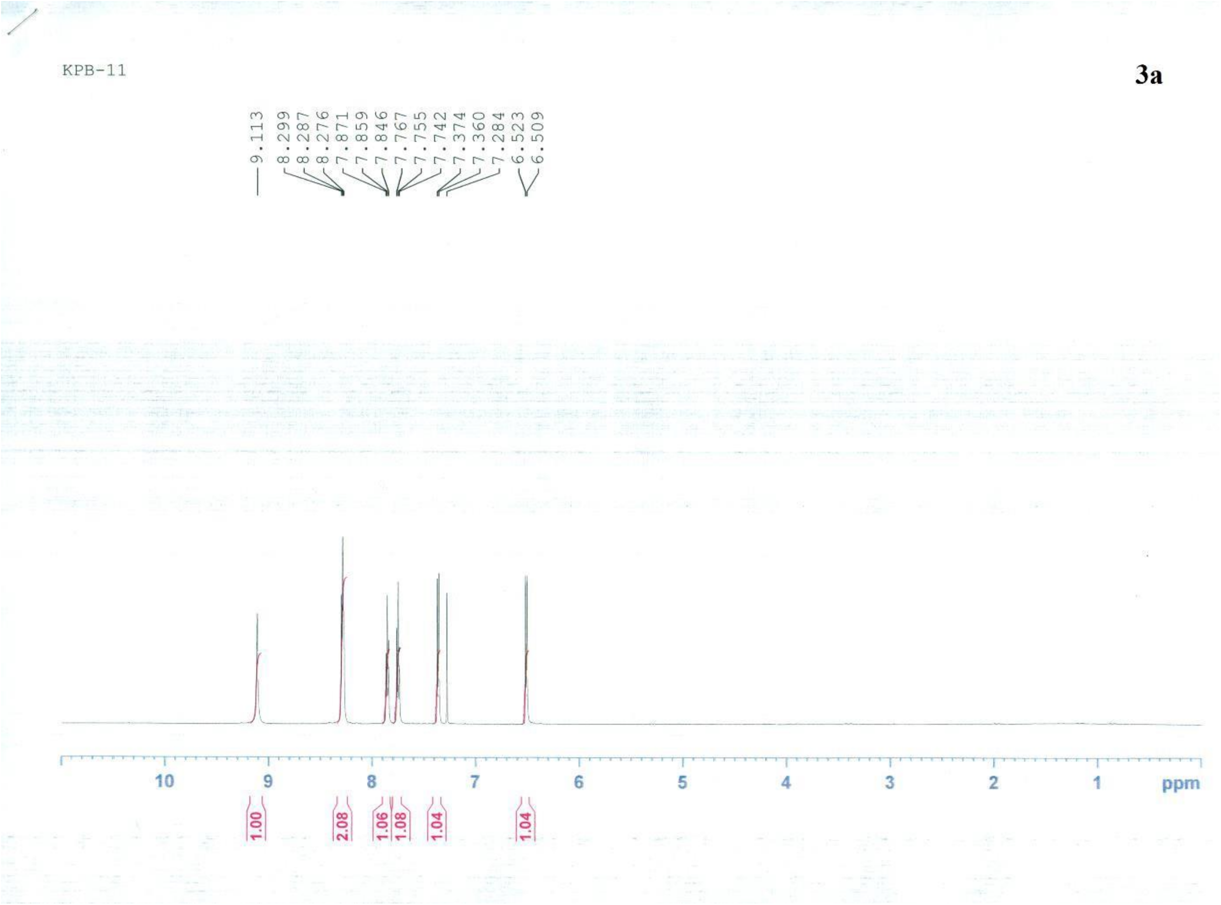

^{1}H NMR of 14-(4-cyanophenyl)diquinothiazine (**3a**)

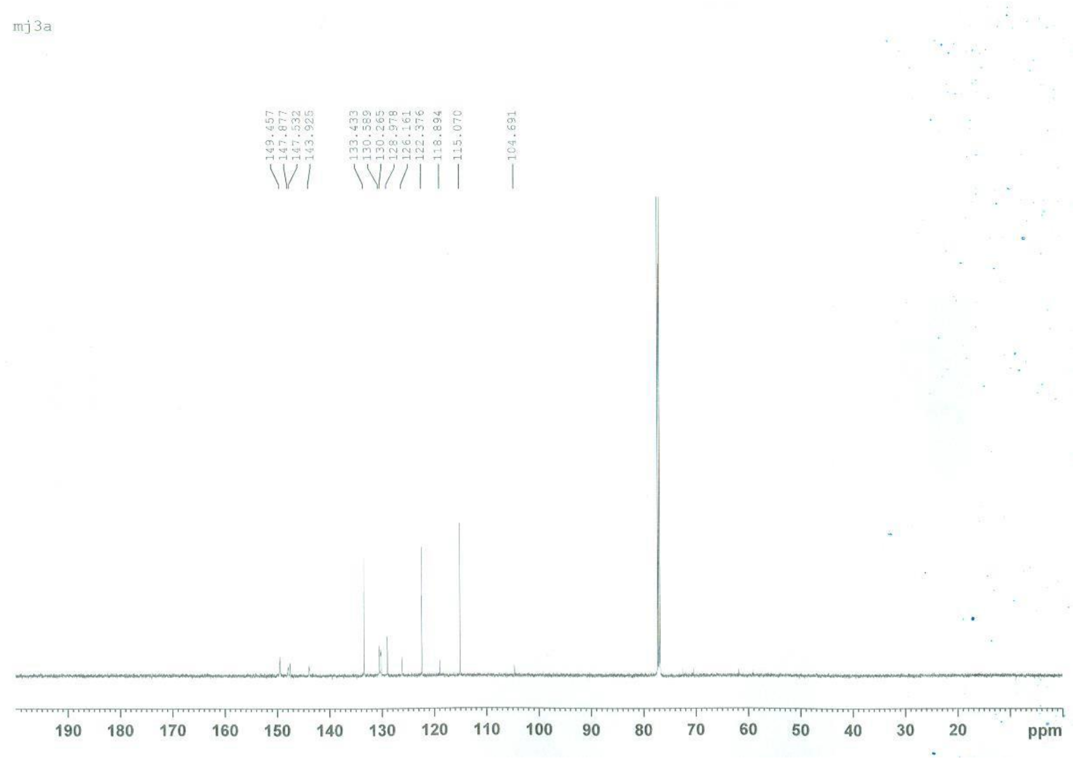

^{13}C NMR of 14-(4-cyanophenyl)diquinothiazine (**3a**)

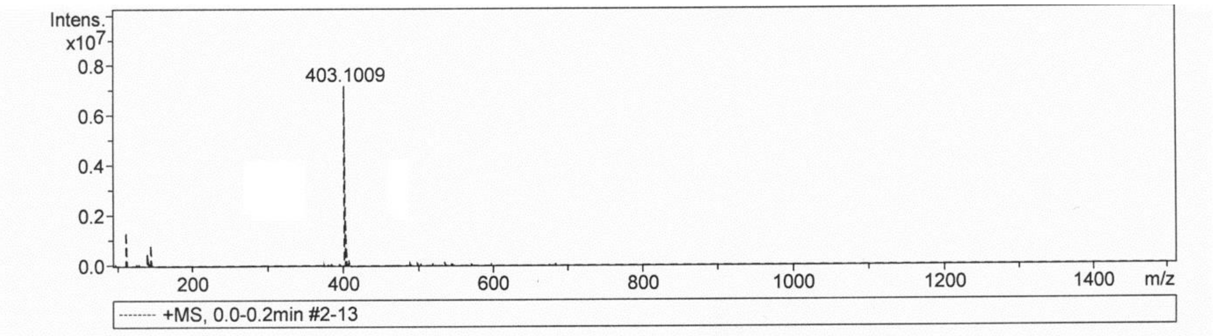

#	m/z	Res.	S/N	I	I %	FWHM
1	403.1009	43385	13067.4	7174089	100.0	0.0093

HR MS of 14-(4-cyanophenyl)diquinothiazine (**3a**) (calcd m/z = 403.1007)

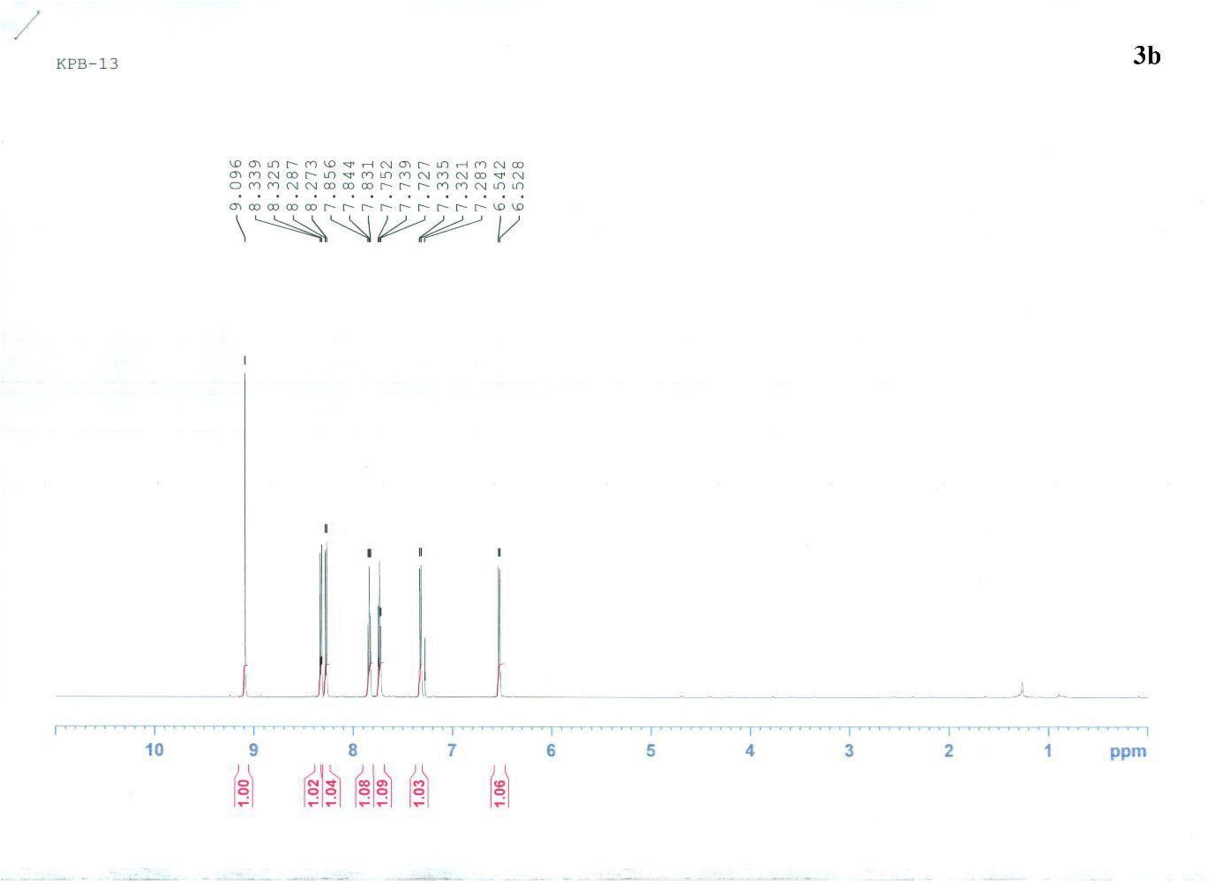

3b

^{1}H NMR of 14-(4-trifluoromethylphenyl)diquinothiazine (**3b**)

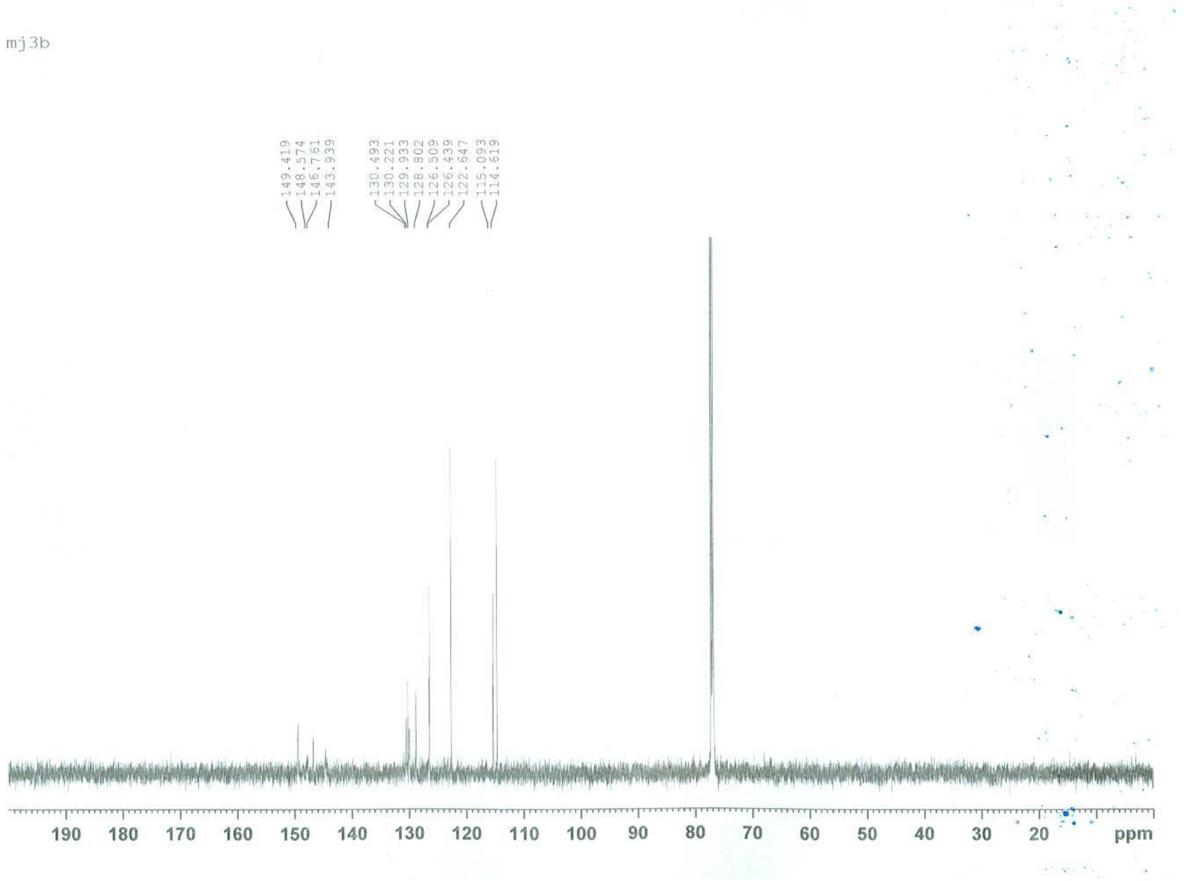

^{13}C NMR of 14-(4-trifluoromethylphenyl)diquinothiazine (**3b**)

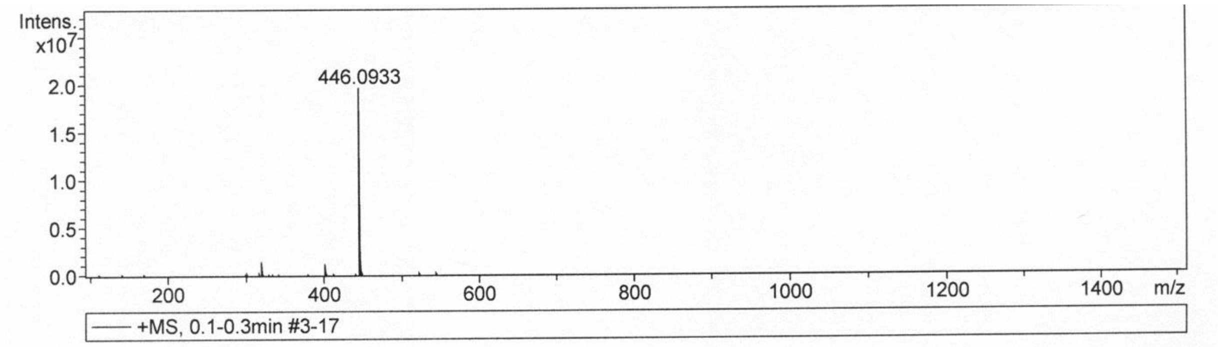

#	m/z	Res.	S/N	I	I %	FWHM
1	446.0933	18366	74183.1	19566588	100.0	0.0243

HR MS of 14-(4-trifluoromethylphenyl)diquinothiazine (**3b**) (calcd m/z = 446.0939)

3c

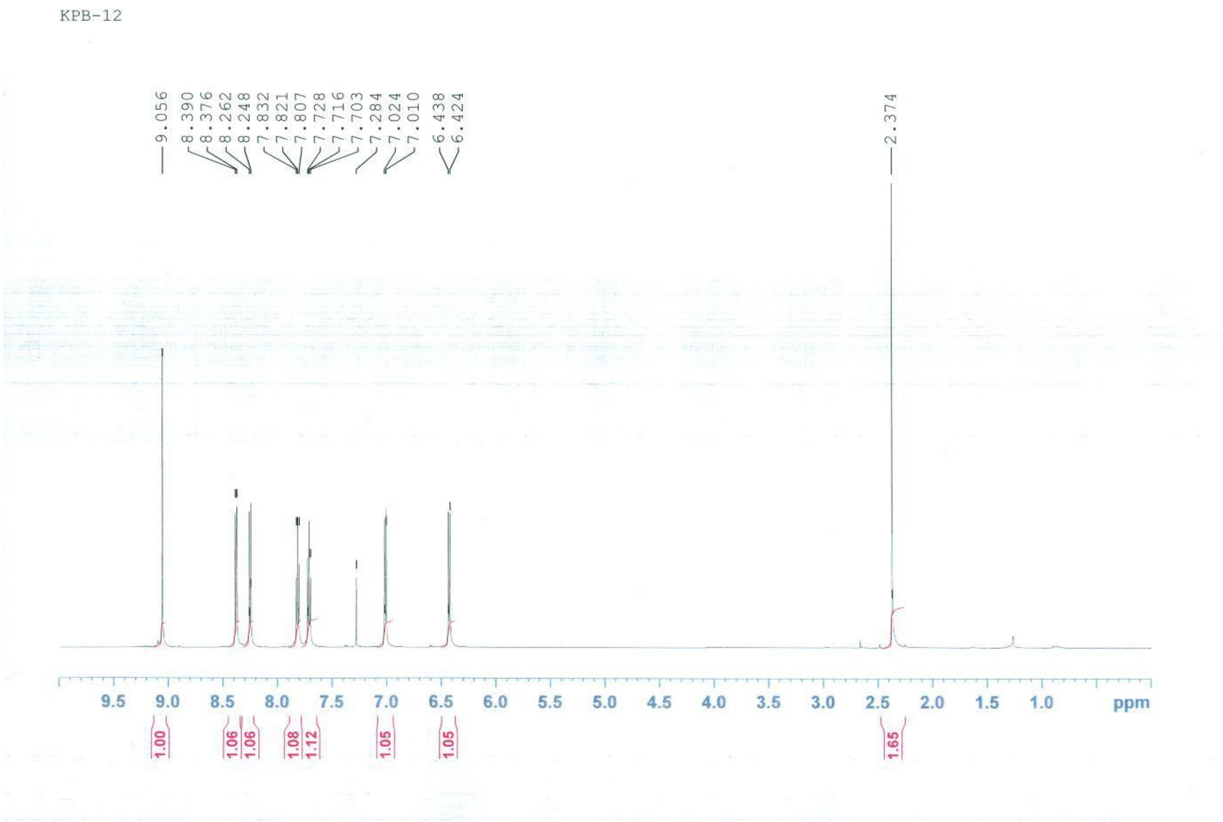

^{1}H NMR of 14-(methylthiophenyl)diquinothiazine (**3c**)

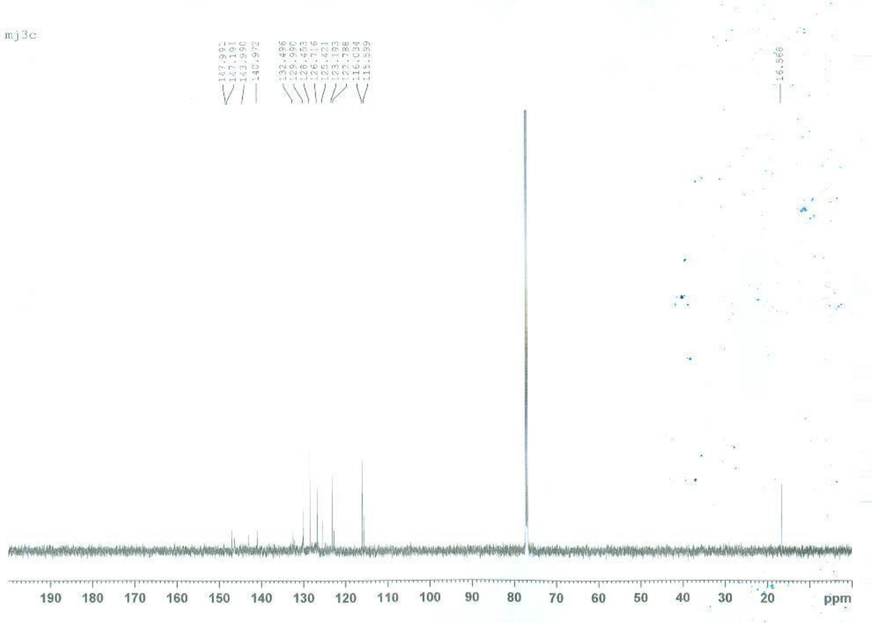

^{13}C NMR of 14-(methylthiophenyl)diquinothiazine (**3c**)

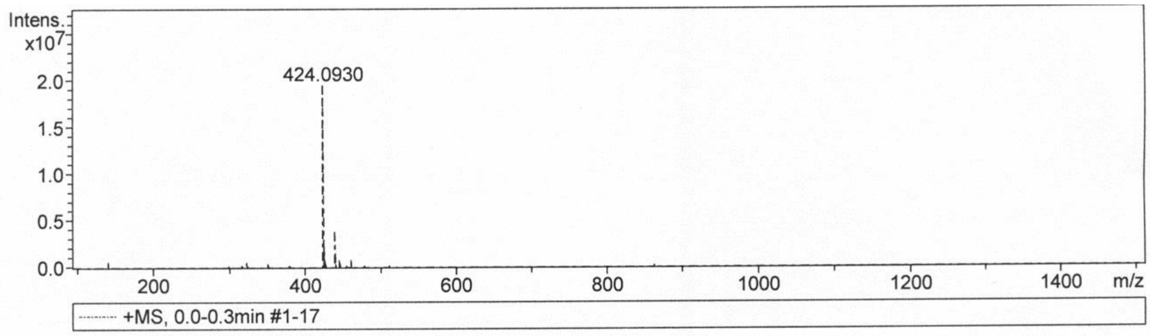

#	m/z	Res.	S/N	I	I %	FWHM
1	424.0930	26963	112446.6	19423724	100.0	0.0157

HR MS of 14-(methylthiophenyl)diquinothiazine (**3c**) (calcd m/z = 424.0942)

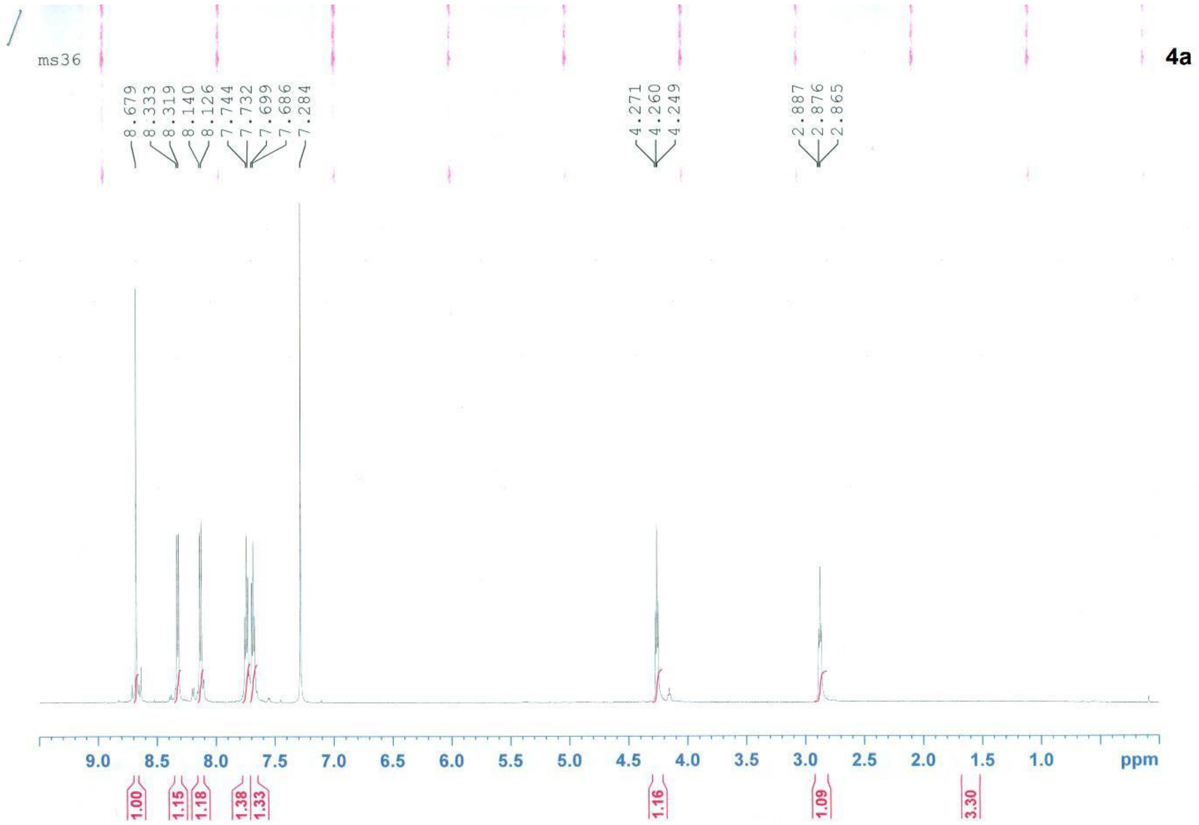

^{1}H NMR of 14-aminoethyldiquinothiazine (**4a**)

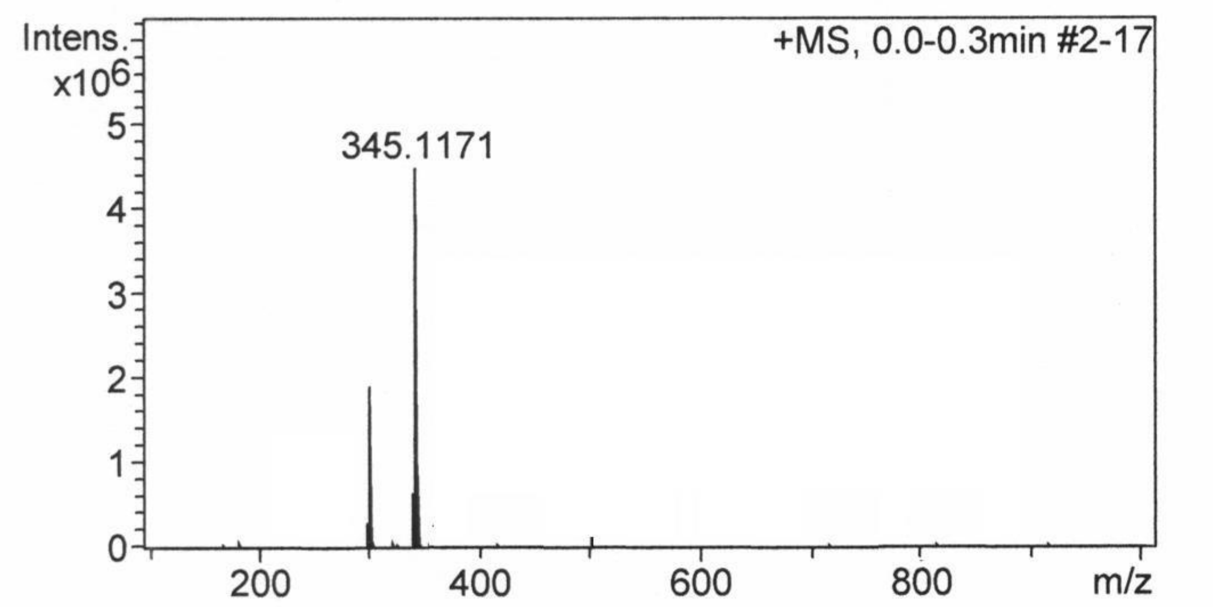

#	m/z	Res.	S/N	I	I %	FWHM
1	302.0748	35795	26541.8	1916610	42.7	0.0084
2	345.1171	40659	50348.4	4490263	100.0	0.0085

HR MS of 14-aminoethyldiquinothiazine (**4a**) (calcd m/z = 345.1174)

4b

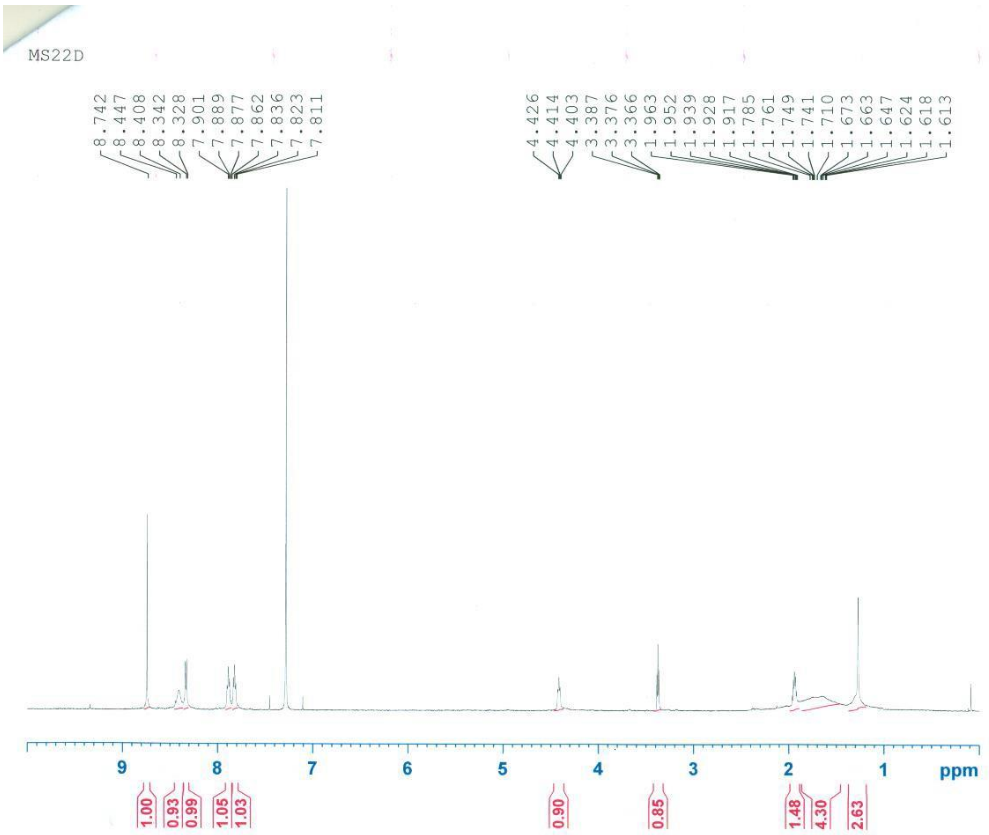

^{1}H NMR of 14-aminoprophyldiquinothiazine (**4b**)

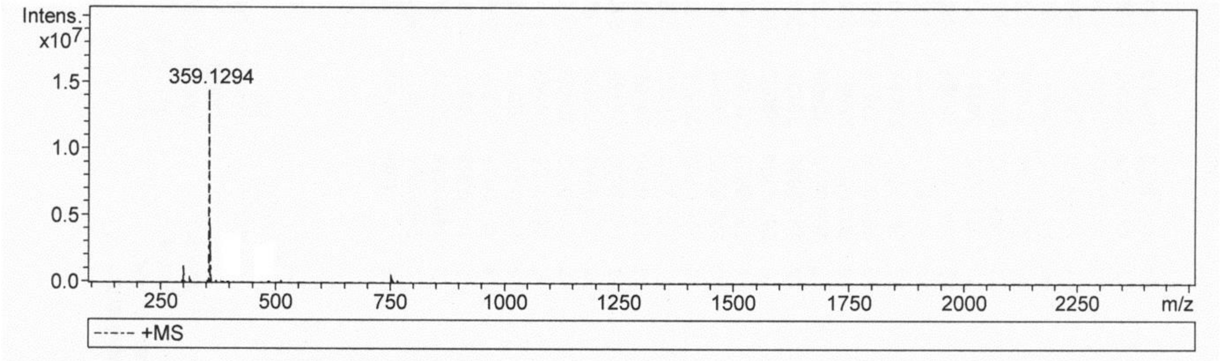

#	m/z	Res.	S/N	I	I %	FWHM
1	359.1294	31209	59662.2	14536520	100.0	0.0115

HR MS of 14-aminoprophyldiquinothiazine (**4b**) (calcd m/z = 359.1330)

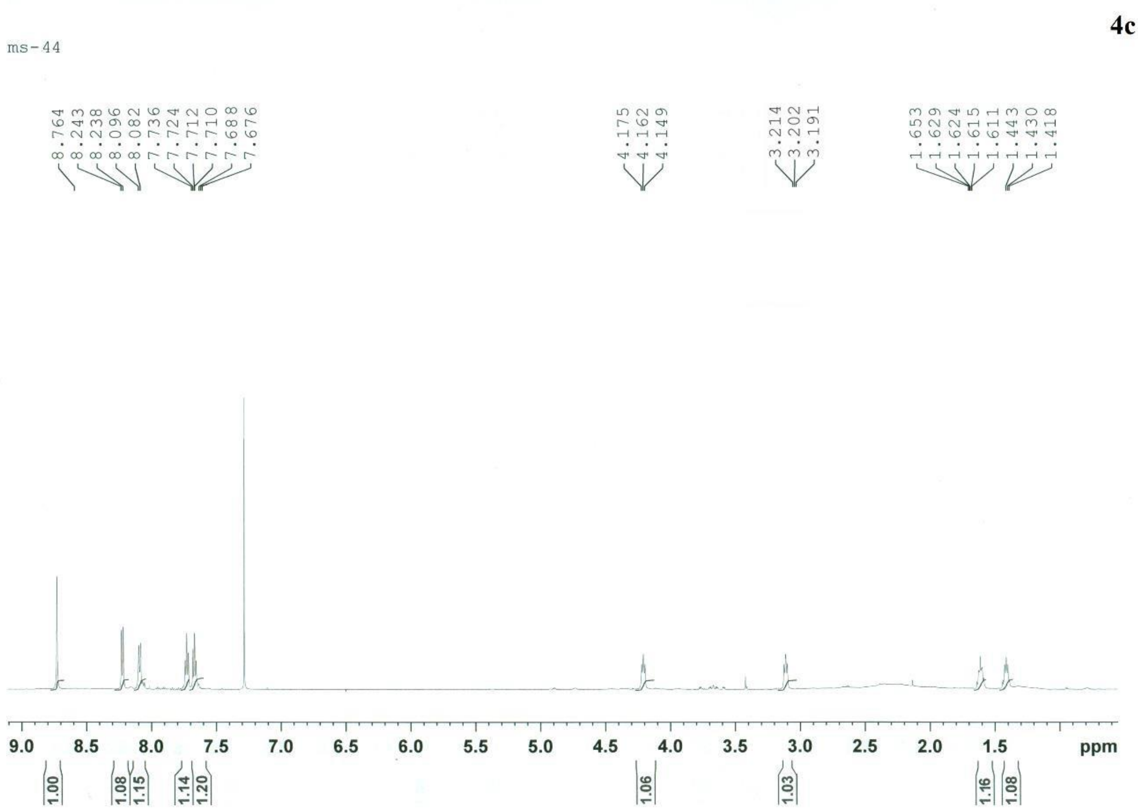

^{1}H NMR of 14-aminobuthyldiquinothiazine (**4c**)

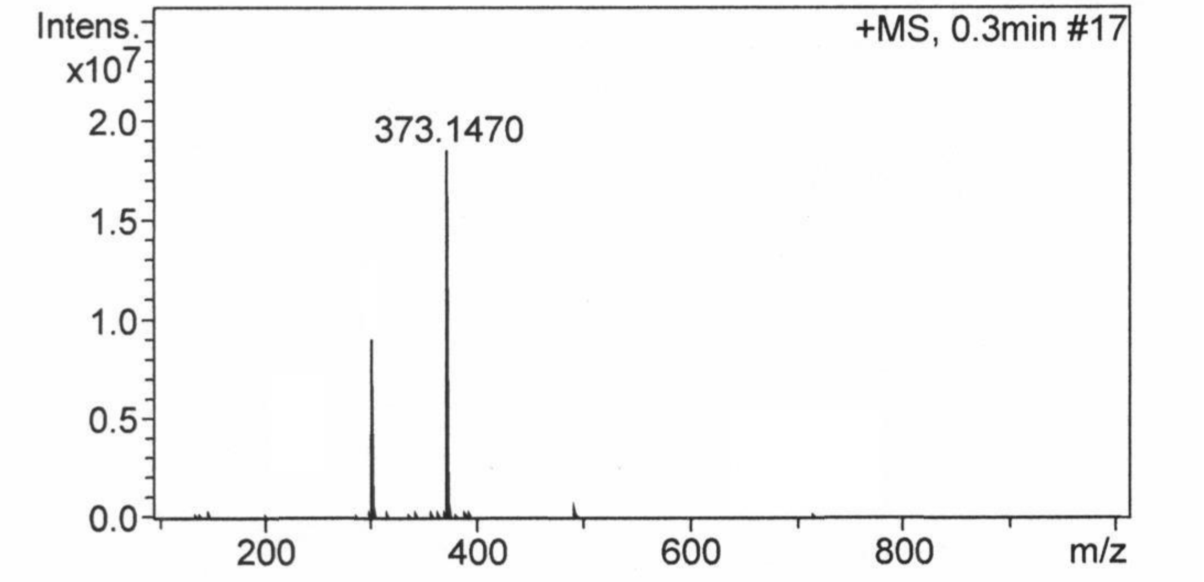

#	m/z	Res.	S/N	I	I %	FWHM
1	302.0736	40175	46209.3	12777708	49.2	0.0075
2	373.1470	40073	51771.1	18462910	100.0	0.0093

HR MS of 14-aminobuthyldiquinothiazine (**4c**) (calcd m/z = 373.1487)

5a

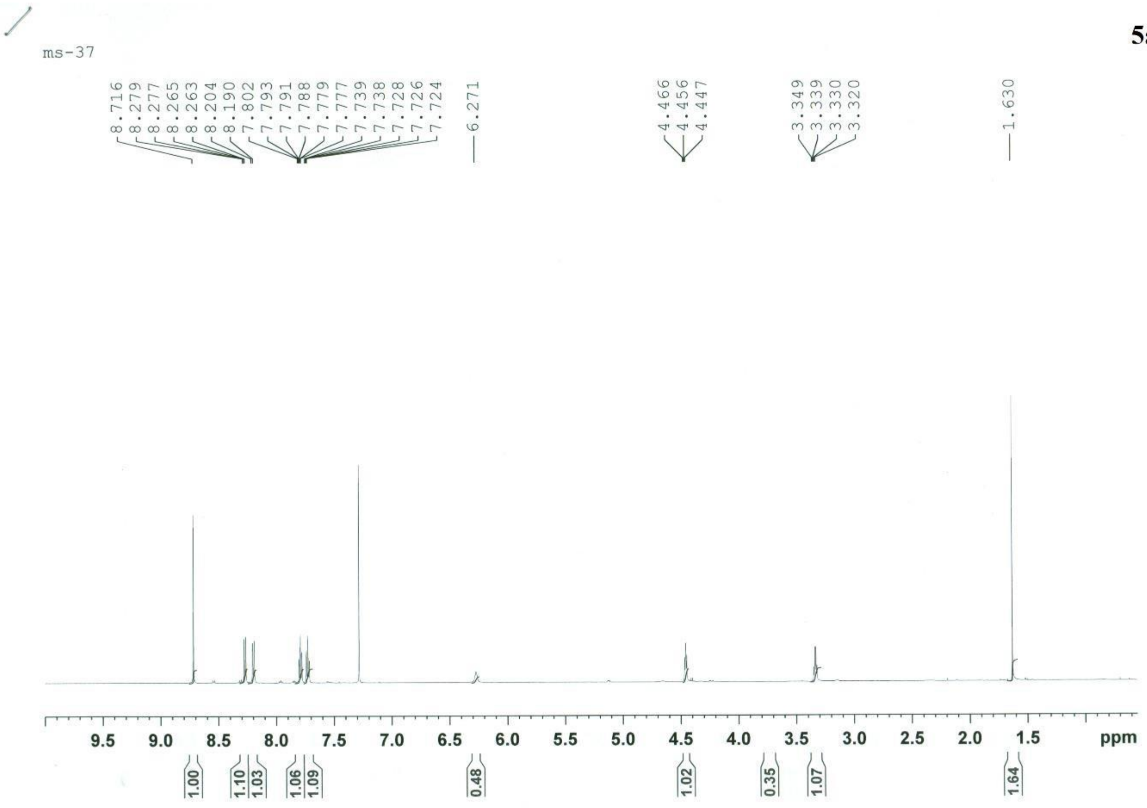

^{1}H NMR of 14-acetylaminoethyldiquinothiazine (**5a**)

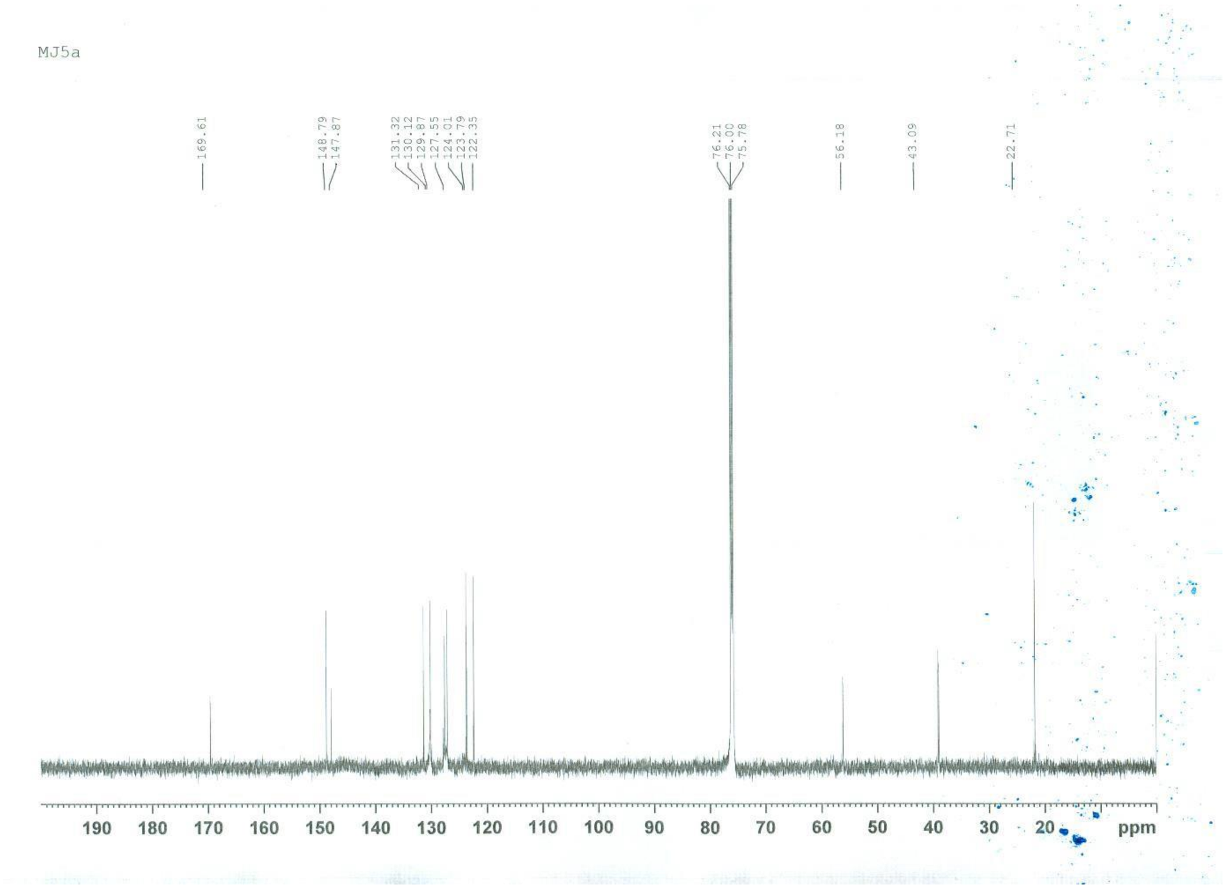

^{13}C NMR of 14-acetylaminoethyldiquinothiazine (**5a**)

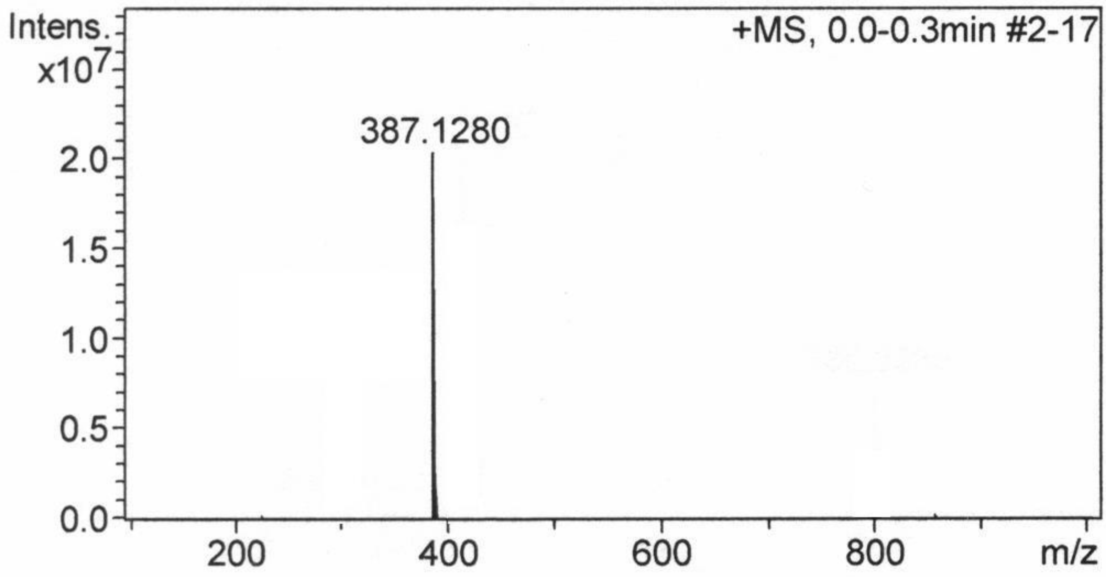

#	m/z	Res.	S/N	I	I %	FWHM
1	387.1280	19027	47882.4	20377582	100.0	0.0203

HR MS of 14-acetylaminoethyldiquinothiazine (**5a**) (calcd m/z = 387.1280)

5b

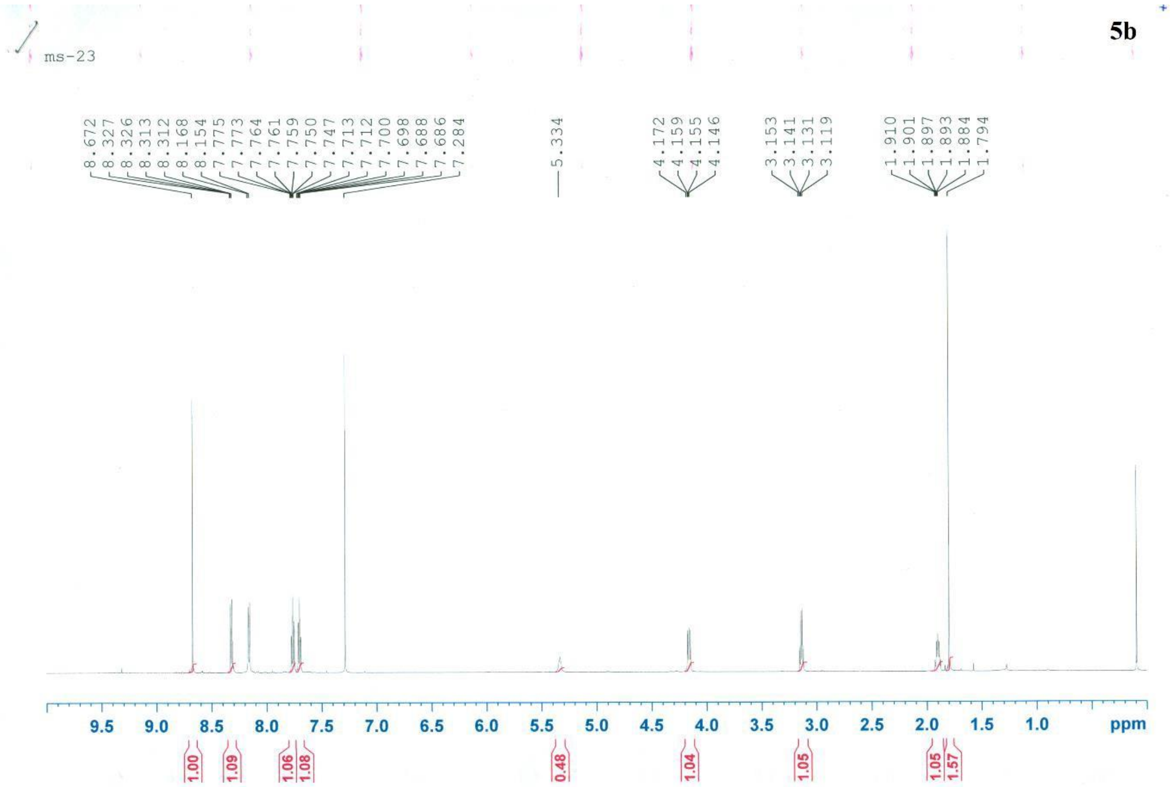

^{1}H NMR of 14-acetylaminopropyldiquinothiazine (**5b**)

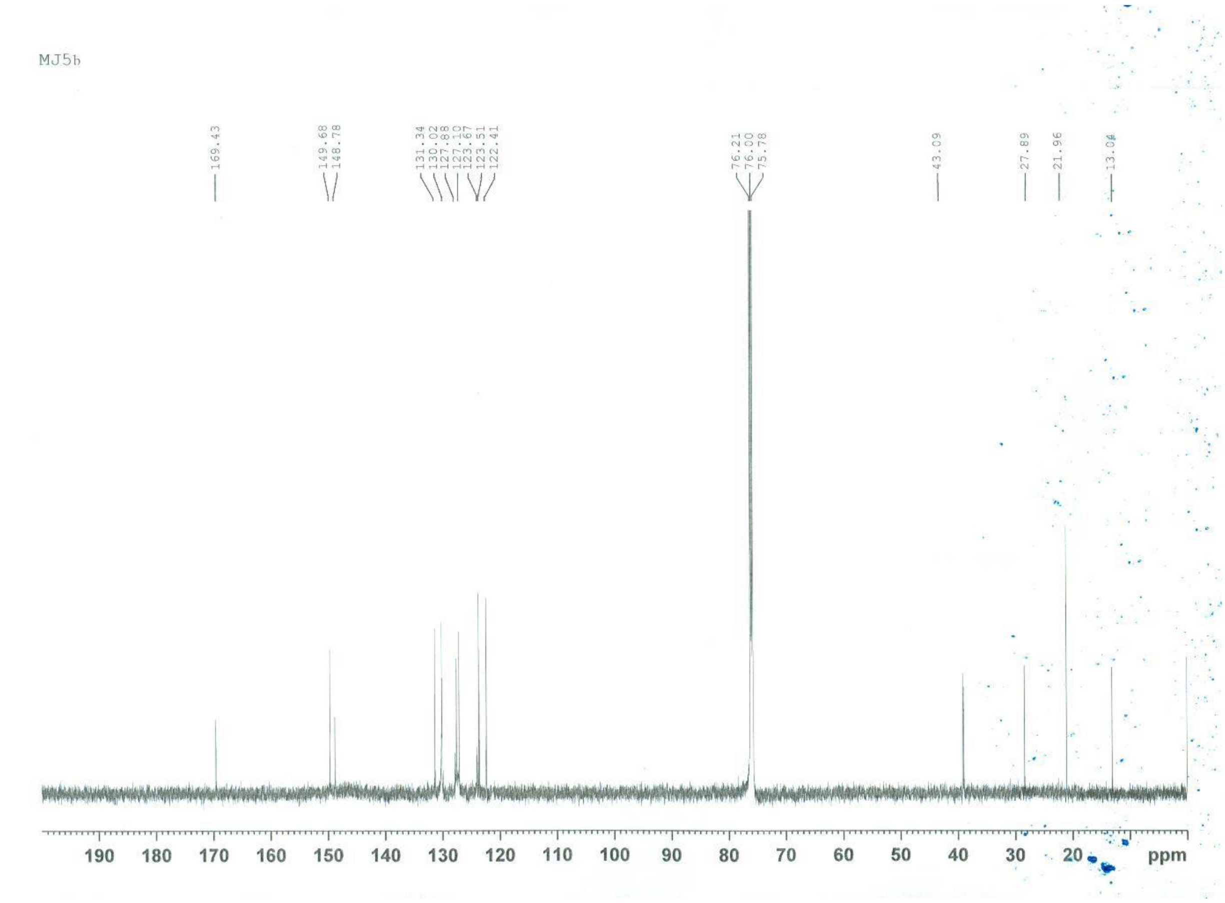

^{13}C NMR of 14-acetylaminopropyldiquinothiazine (**5b**)

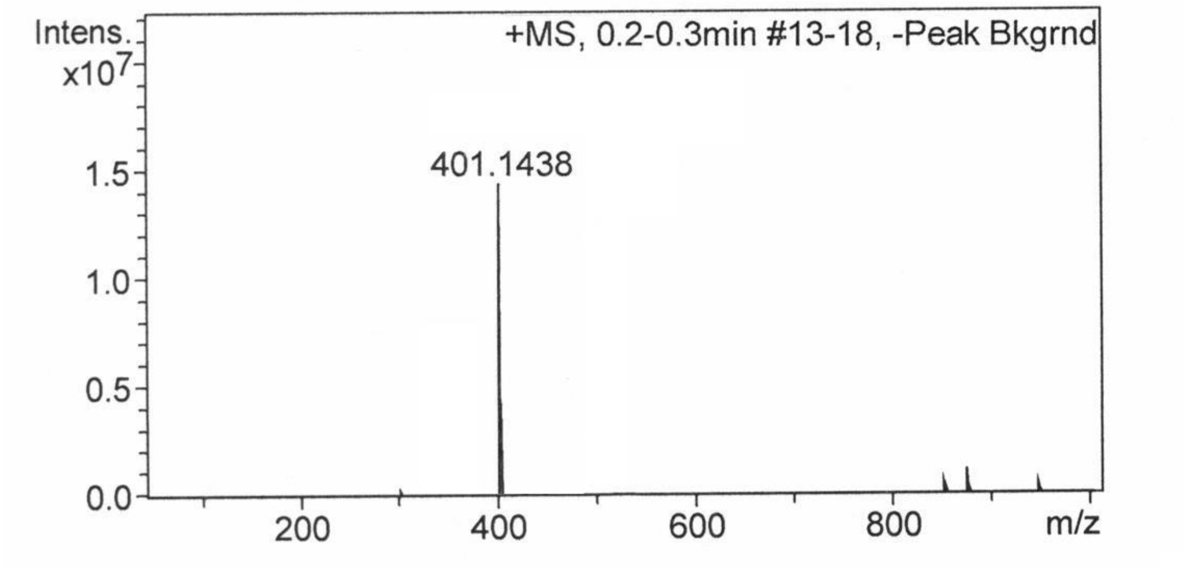

#	m/z	Res.	S/N	I	I %	FWHM
1	401.1438	40276	12.3	123	99.3	0.0100

HR MS of 14-acetylaminopropyldiquinothiazine (**5b**) (calcd m/z = 401.1436)

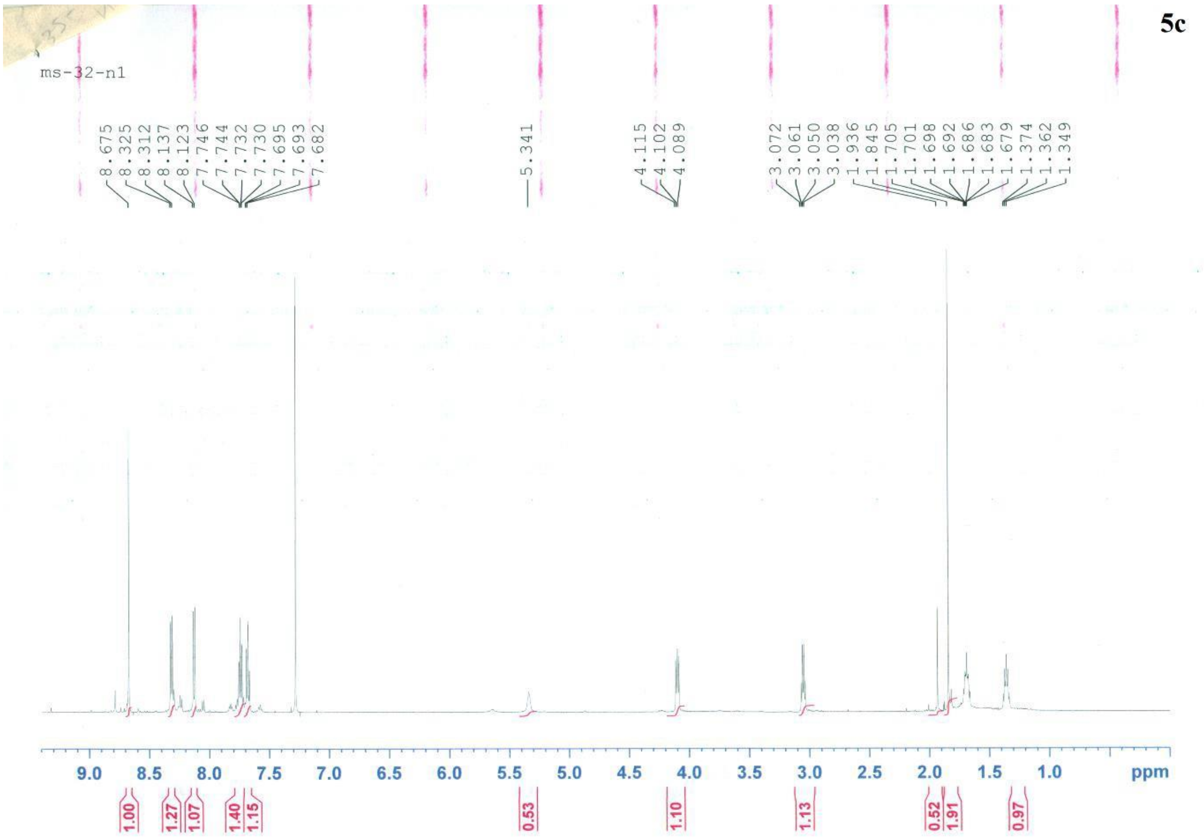

^{1}H NMR of 14-acetylaminobutyldiquinothiazine (**5c**)

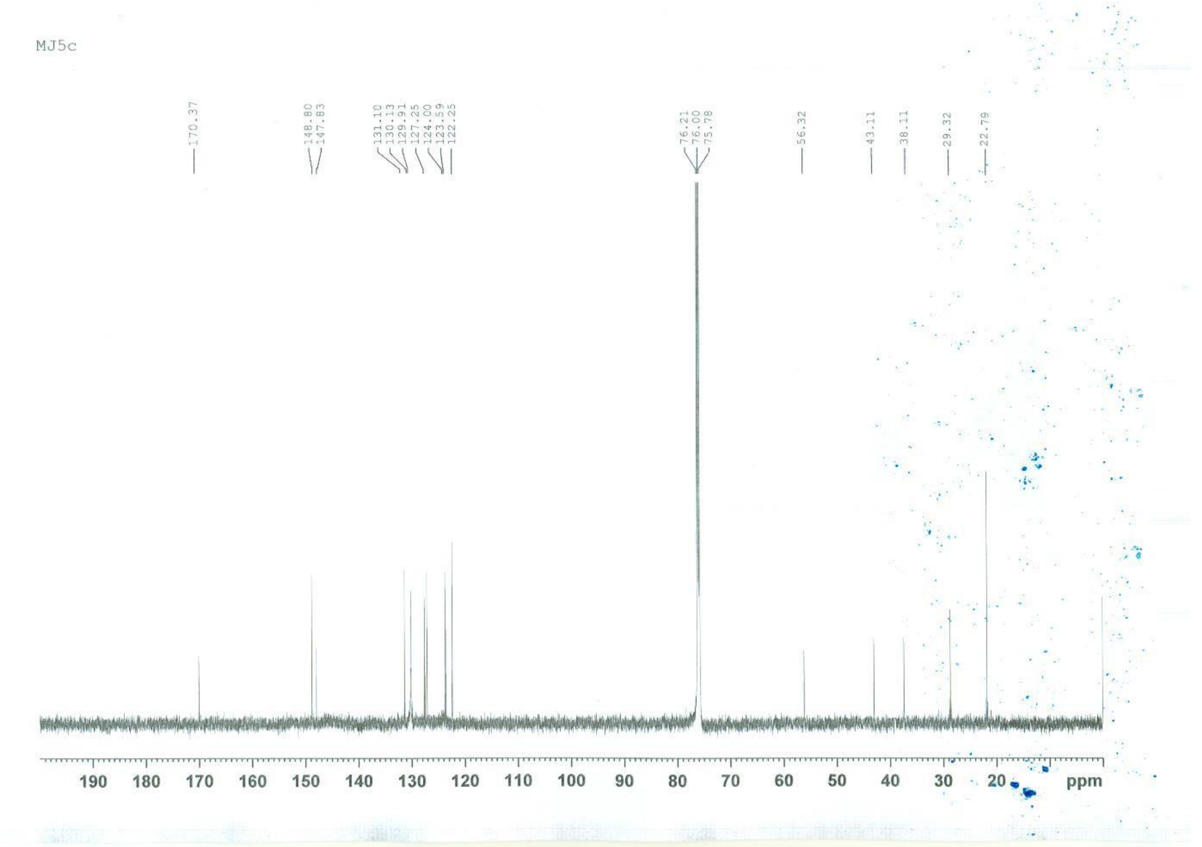

^{13}C NMR of 14-acetylaminobutyldiquinothiazine (**5c**)

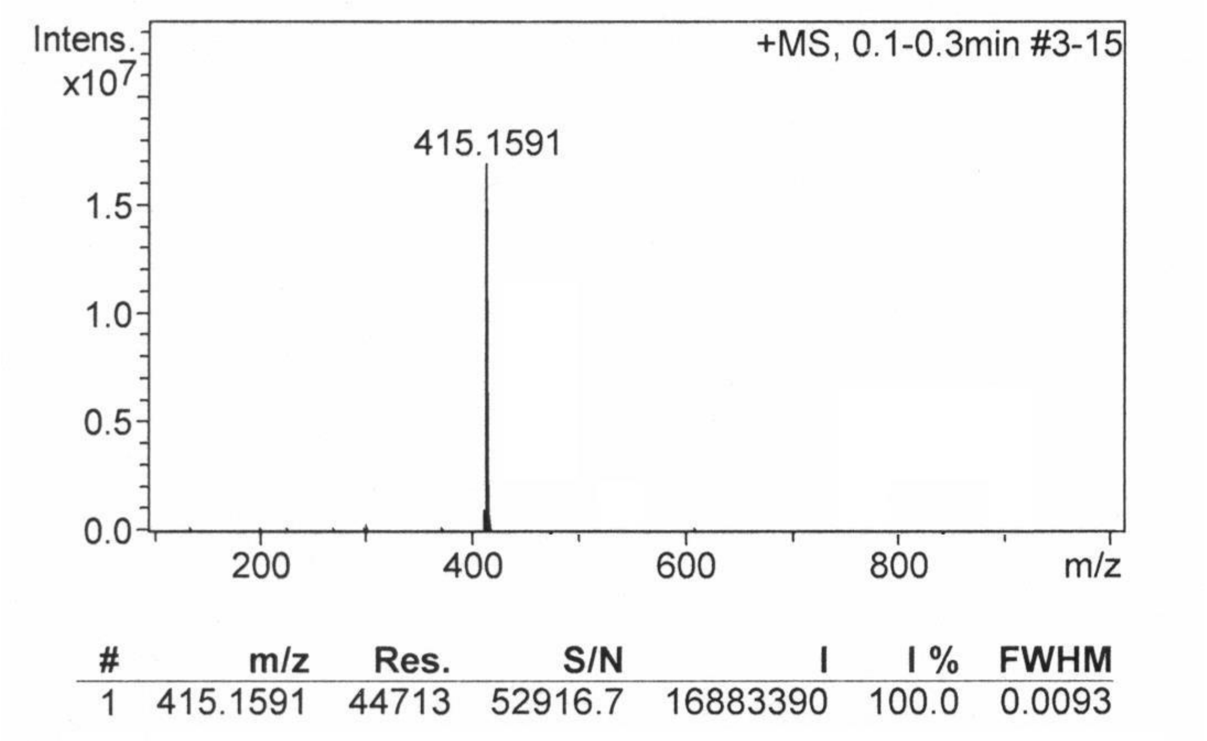

HR MS of 14-acetylaminobutyldiquinothiazine (**5c**) (calcd m/z = 415.1593)

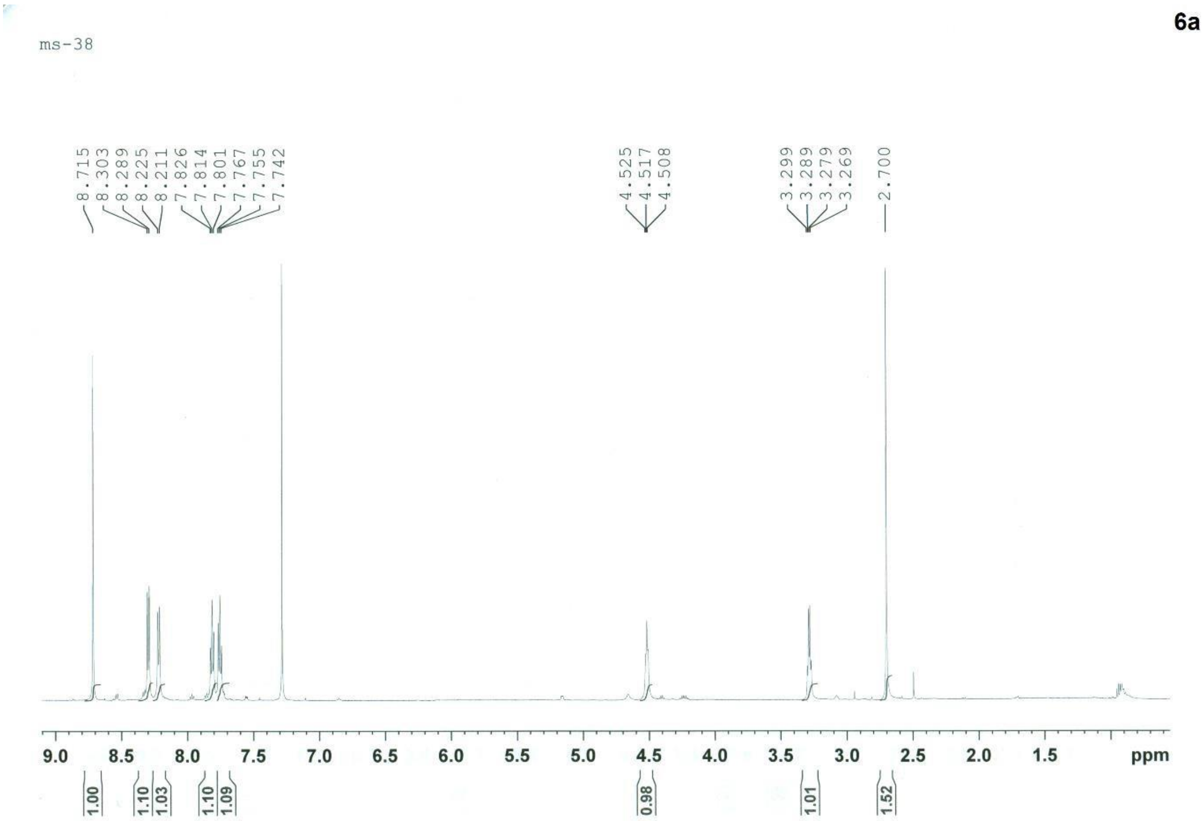

^{1}H NMR of 14-methanesulfonylaminoethyldiquinothiazine (**6a**)

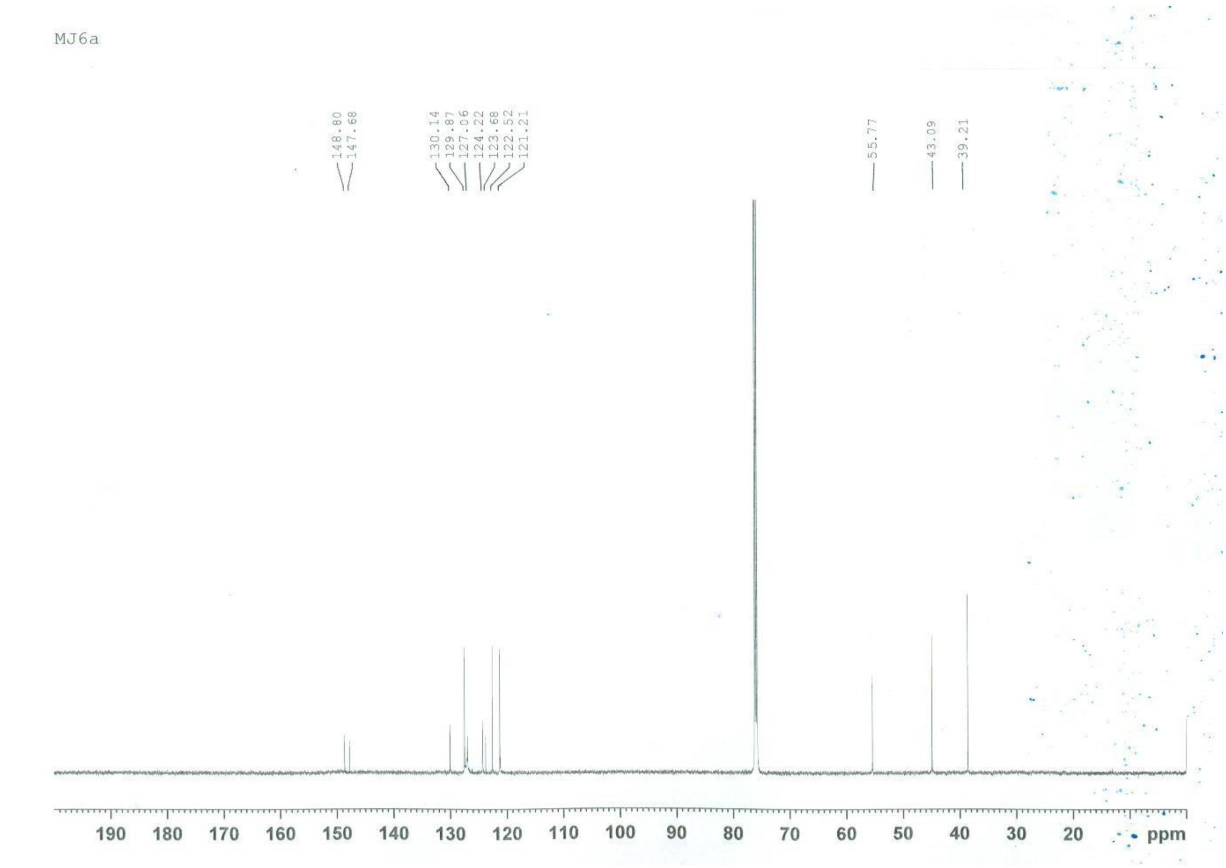

^{13}C NMR of 14-methanesulfonylaminoethyldiquinothiazine (**6a**)

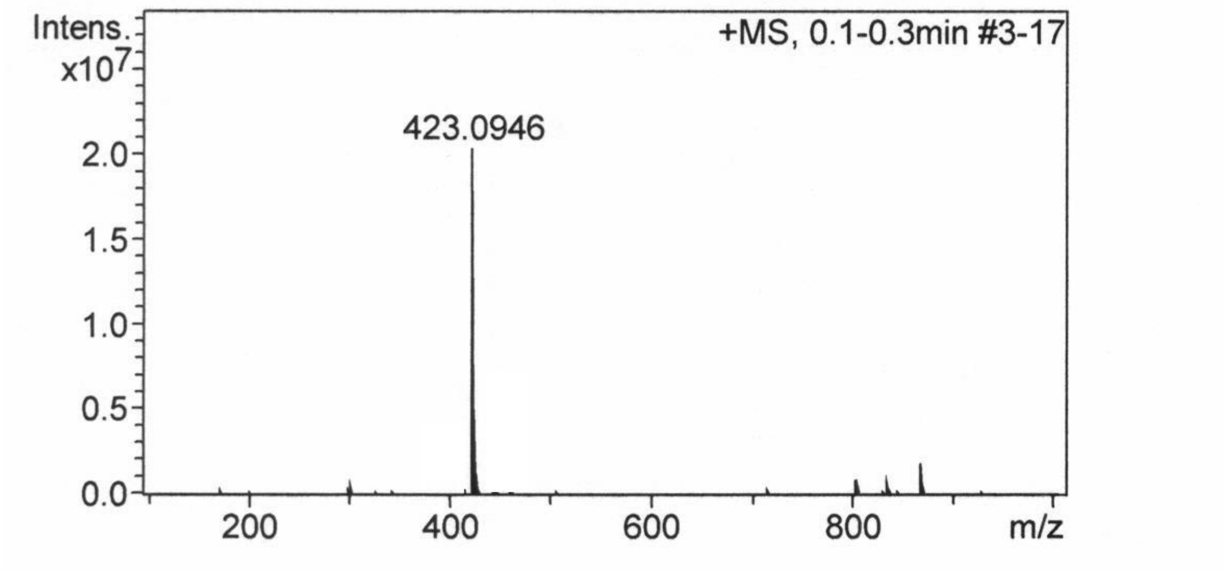

#	m/z	Res.	S/N	I	I %	FWHM
1	423.0946	16772	37580.9	20377596	100.0	0.0252

HR MS of 14-methanesulfonylaminoethyldiquinothiazine (**6a**) (calcd m/z = 423.0949)

6b

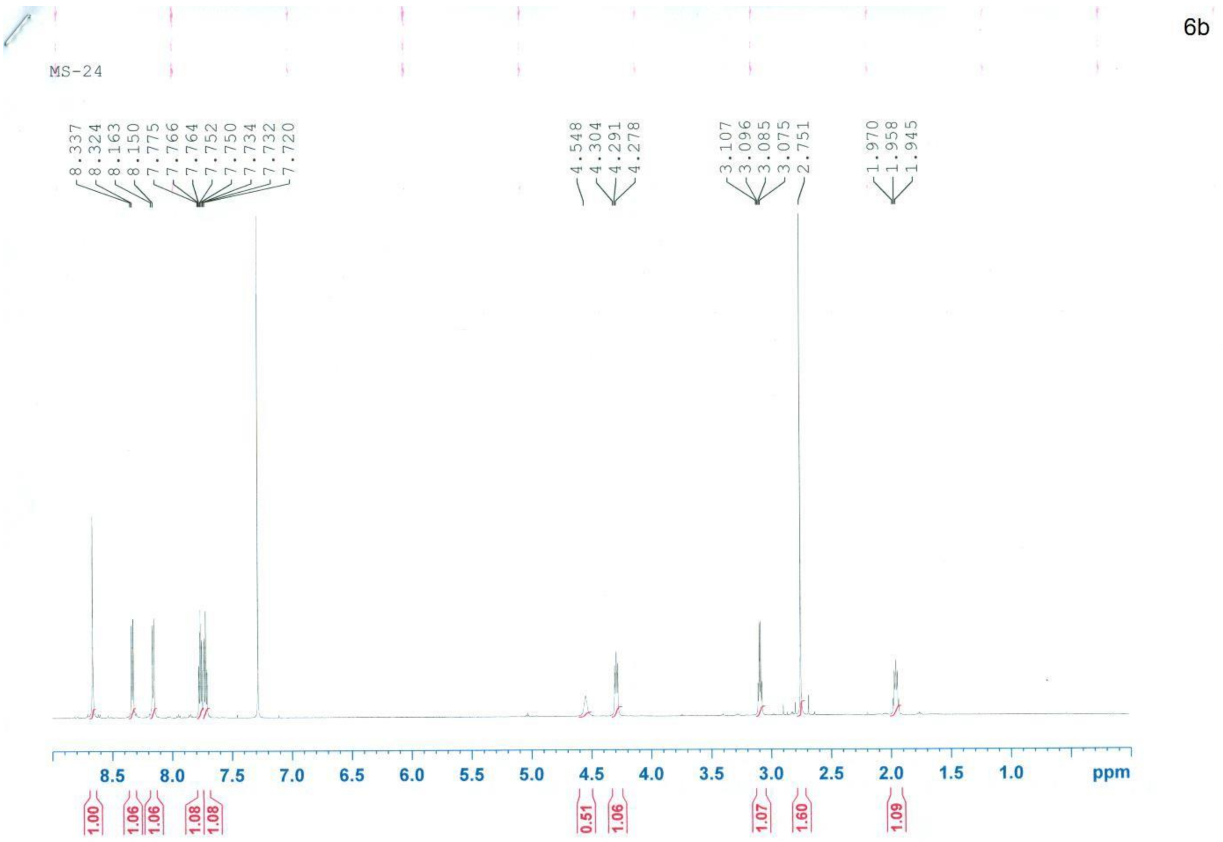

^{1}H NMR of 14-methanesulfonylaminopropyldiquinothiazine (**6b**)

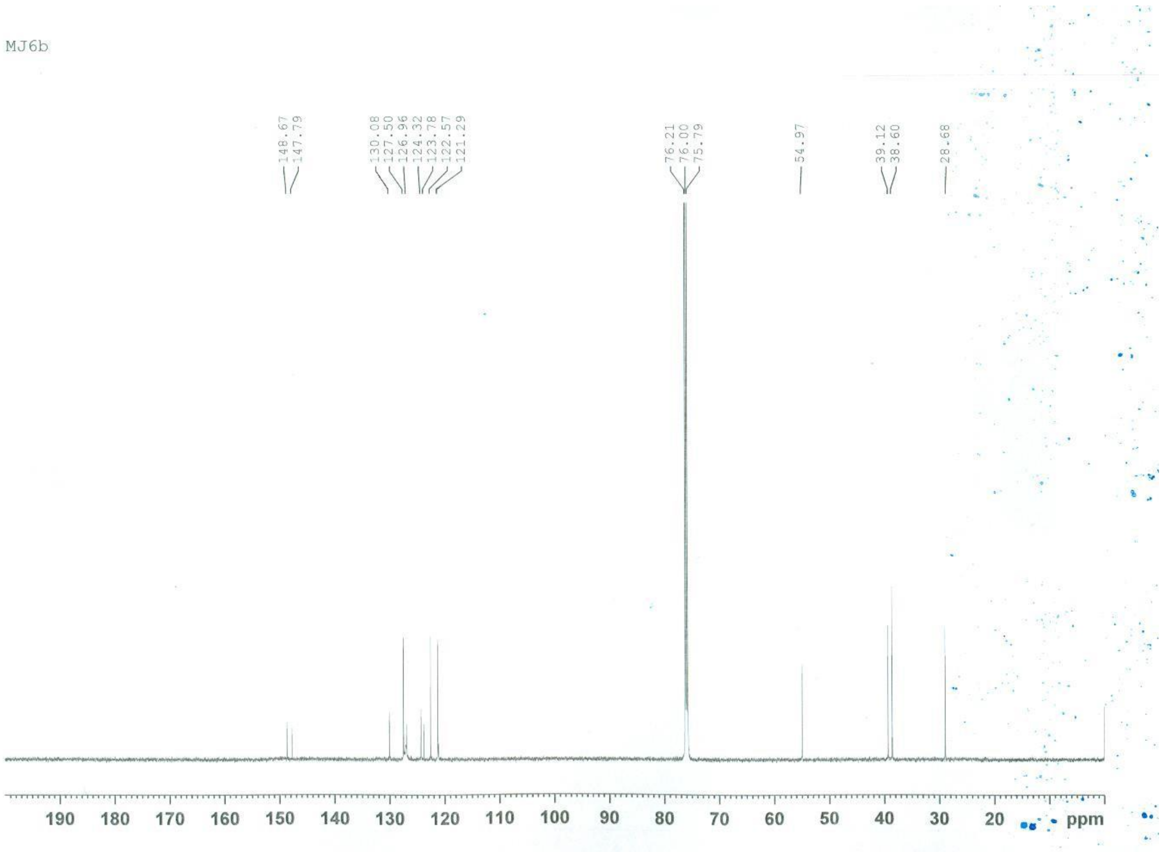

^{13}C NMR of 14-methanesulfonylaminopropyldiquinothiazine (**6b**)

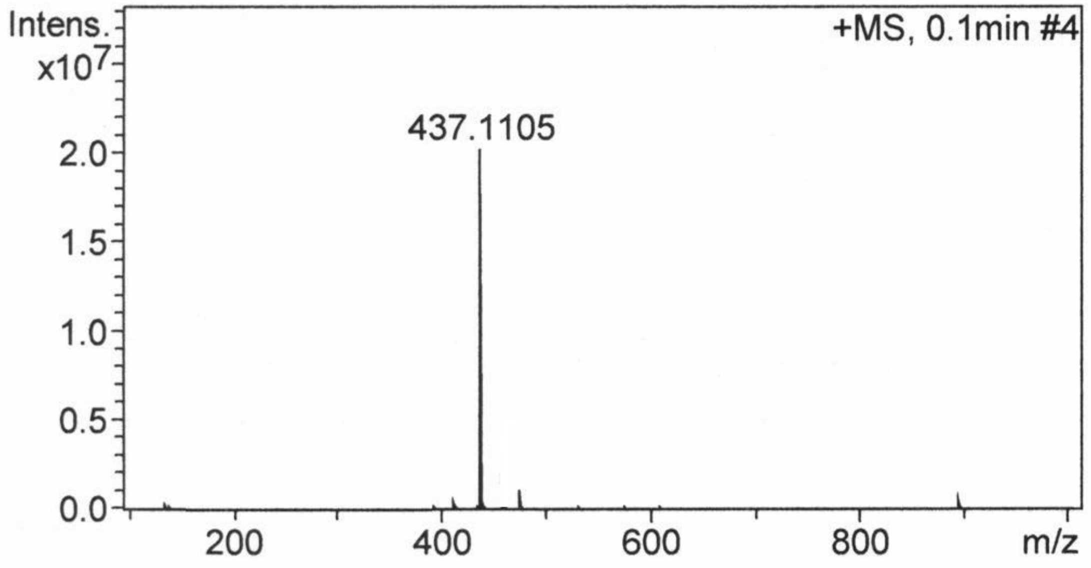

#	m/z	Res.	S/N	I	I %	FWHM
1	437.1105	30430	53754.7	20217262	100.0	0.0144

HR MS of 14-methanesulfonylaminopropyldiquinothiazine (**6b**) (calcd m/z = 437.1106)

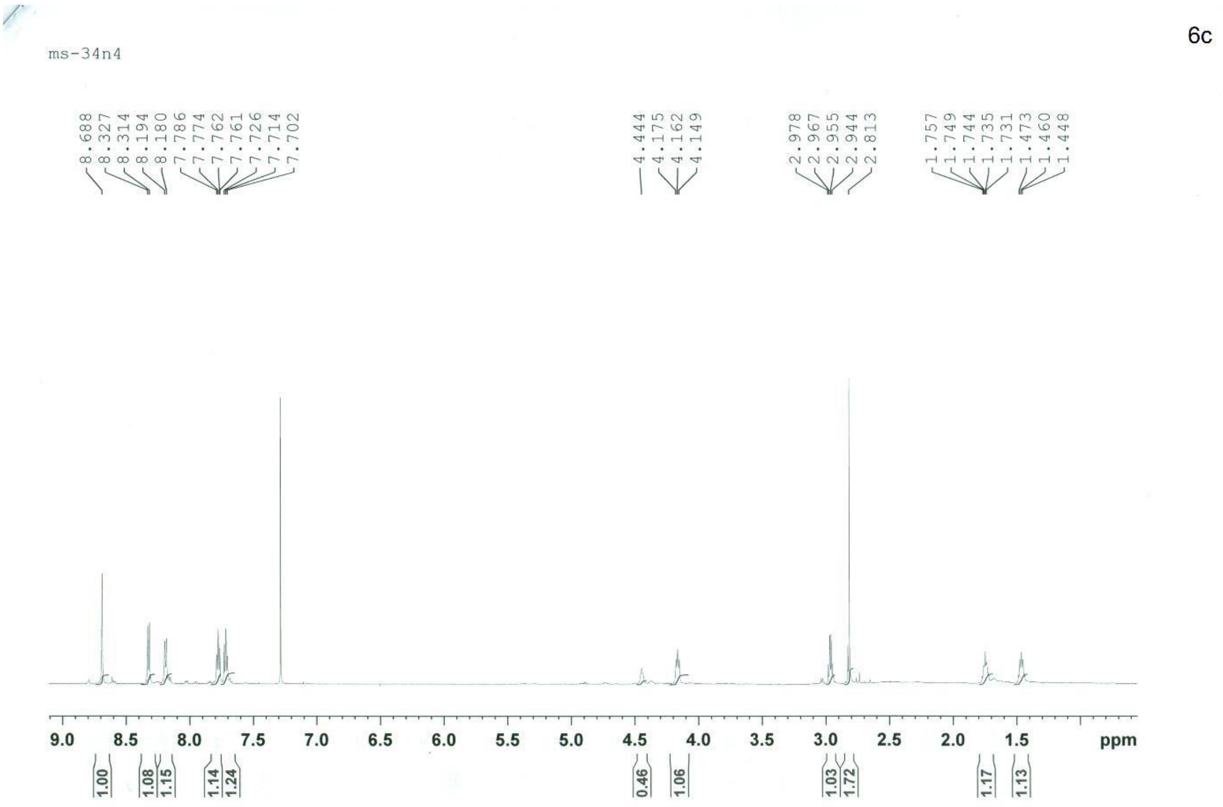

^{1}H NMR of 14-methanesulfonylaminobutyldiquinothiazine (**6c**)

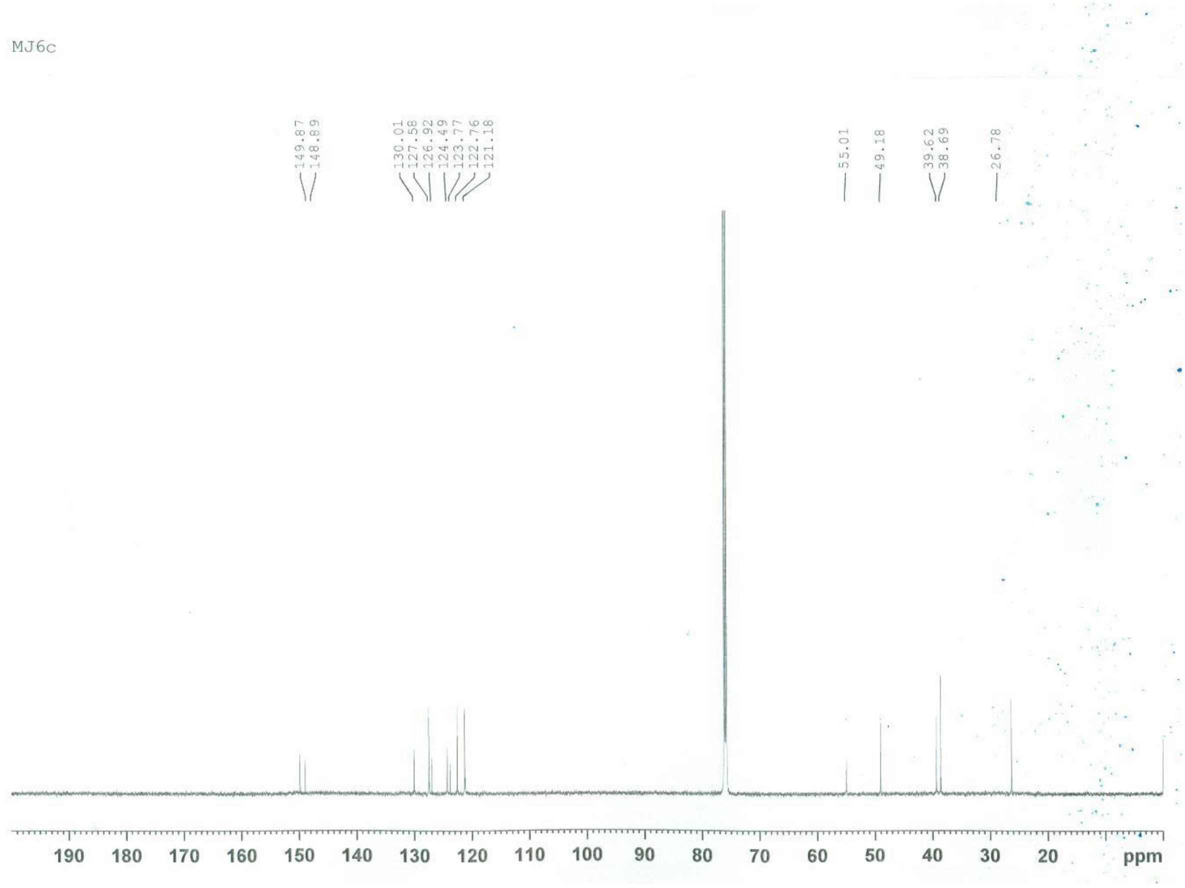

^{13}C NMR of 14-methanesulfonylaminobutyldiquinothiazine (**6c**)

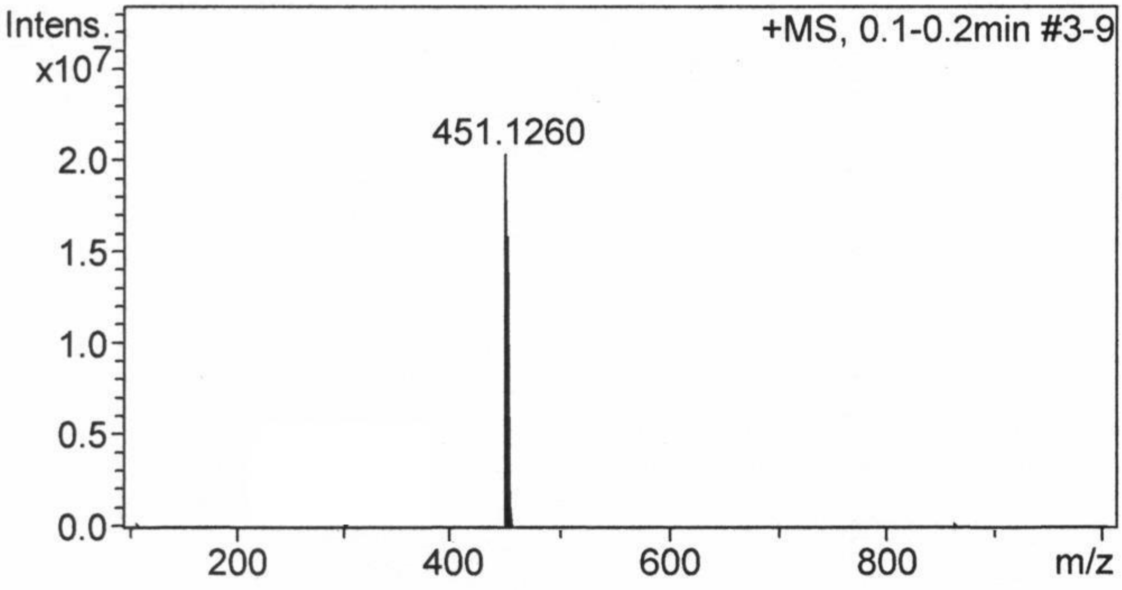

#	m/z	Res.	S/N	I	I %	FWHM
1	451.1260	16151	37072.7	20377596	100.0	0.0279

HR MS of 14-methanesulfonylaminobutyldiquinothiazine (**6c**) (calcd m/z = 451.1262)
